# Sequential glycosylations at the multibasic cleavage site of SARS-CoV-2 spike protein regulate viral activity

**DOI:** 10.1038/s41467-024-48503-x

**Published:** 2024-05-16

**Authors:** Shengjun Wang, Wei Ran, Lingyu Sun, Qingchi Fan, Yuanqi Zhao, Bowen Wang, Jinghong Yang, Yuqi He, Ying Wu, Yuanyuan Wang, Luoyi Chen, Arpaporn Chuchuay, Yuyu You, Xinhai Zhu, Xiaojuan Wang, Ye Chen, Yanqun Wang, Yao-Qing Chen, Yanqiu Yuan, Jincun Zhao, Yang Mao

**Affiliations:** 1 School of Pharmaceutical Sciences, Sun Yat-sen University, Guangzhou, China; 2 State Key Laboratory of Respiratory Disease, National Clinical Research Center for Respiratory Disease, Guangzhou Institute of Respiratory Health, The First Affiliated Hospital of Guangzhou Medical University, Guangzhou, China; 3 College of Life Science, Northwest University, Xi’an, China; 4 School of Public Health (Shenzhen), Shenzhen Campus of Sun Yat-sen University, Shenzhen, China; 5 Instrumental Analysis & Research Center, Sun Yat-sen University, Guangzhou, China; 6 Sun Yat-sen Memorial Hospital, Sun Yat-sen University, Guangzhou, China; 7 Key Laboratory of Fujian-Taiwan Animal Pathogen Biology, College of Animal Sciences, Fujian Agriculture and Forestry University, Fuzhou, China; 8 State Key Laboratory of Anti-Infective Drug Discovery and Development, School of Pharmaceutical Sciences, Sun Yat-sen University, Guangzhou, China; 9 Institute of Infectious Disease, Guangzhou Eighth People’s Hospital of Guangzhou Medical University, Guangzhou, China; 10 Guangzhou Laboratory, Bio-island, Guangzhou, China; 11 The Second Affiliated Hospital, School of Medicine, Southern University of Science and Technology, Shenzhen, China; 12 Shanghai Institute for Advanced Immunochemical Studies, School of Life Science and Technology, ShanghaiTech University, Shanghai, China; 13 Institute for Hepatology, National Clinical Research Center for Infectious Disease, Shenzhen Third People’s Hospital, Shenzhen, China; 14 Guangdong Provincial Key Laboratory of Drug Non-Clinical Evaluation and Research, Guangzhou, China; 15 Present Address: School of Health and Life Sciences, University of Health and Rehabilitation Sciences, Qingdao, China; 16 Present Address: Foshan Institute for Food and Drug Control, Foshan, China

**Keywords:** SARS-CoV-2, Glycosylation, Viral proteins

## Abstract

The multibasic furin cleavage site at the S1/S2 boundary of the spike protein is a hallmark of SARS-CoV-2 and plays a crucial role in viral infection. However, the mechanism underlying furin activation and its regulation remain poorly understood. Here, we show that GalNAc-T3 and T7 jointly initiate clustered *O*-glycosylations in the furin cleavage site of the SARS-CoV-2 spike protein, which inhibit furin processing, suppress the incorporation of the spike protein into virus-like-particles and affect viral infection. Mechanistic analysis reveals that the assembly of the spike protein into virus-like particles relies on interactions between the furin-cleaved spike protein and the membrane protein of SARS-CoV-2, suggesting a possible mechanism for furin activation. Interestingly, mutations in the spike protein of the alpha and delta variants of the virus confer resistance against glycosylation by GalNAc-T3 and T7. In the omicron variant, additional mutations reverse this resistance, making the spike protein susceptible to glycosylation in vitro and sensitive to GalNAc-T3 and T7 expression in human lung cells. Our findings highlight the role of glycosylation as a defense mechanism employed by host cells against SARS-CoV-2 and shed light on the evolutionary interplay between the host and the virus.

## Abstract (web summary)

Here, the authors show that GalNAc-T3 and T7 regulate furin cleavage of the SARS-CoV-2 spike protein via *O*-glycosylation. This influences viral assembly and infection, highlighting glycosylation as a host defense mechanism.

## Introduction

**Fig. 1:**
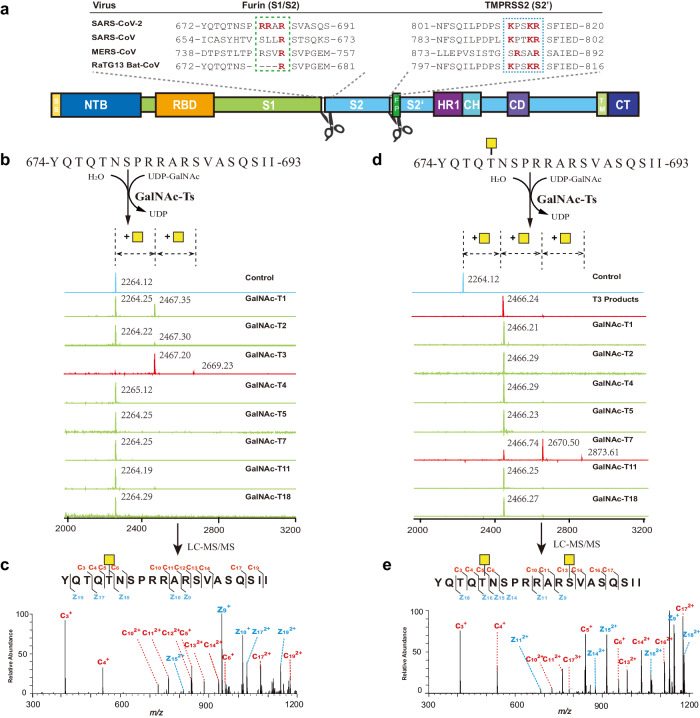
The multibasic cleavage site of SARS-CoV-2 spike protein is glycosylated sequentially by GalNAc-T3 and T7 in vitro. **a** The domain structure of SARS-CoV-2 spike protein and the sequence alignment of listed coronaviruses around SARS-CoV-2 furin site and TMPRSS2 site. **b** MALDI-TOF analysis of GalNAcylation reactions catalyzed by purified GalNAc-Ts on the synthetic multibasic peptide of SARS-CoV-2 spike protein (674-693). Reactions were performed and analyzed as described in the Methods. An increase of 203 Da corresponds to the addition of one GalNAc residue. **c** ETD-MS^2^ spectrum of the *O-*GalNAcylated peptide from the GalNAc-T3 reaction. The mass of *c*- and *z*- fragment ions (e.g. the delta mass between c4 and c5) unambiguously assigned the GalNAc modification to T678. **d** MALDI-TOF analysis of GalNAcylation reactions catalyzed by purified GalNAc-Ts on T678-*O*-GalNAcylated peptide. An increase of 203 Da, corresponding to the modification with an additional GalNAc residue, was only observed with GalNAc-T7. **e** ETD-MS^2^ spectrum of the doubly *O-*GalNAcylated peptide from the GalNAc-T7 reaction. The mass of *c-* and *z*- fragment ions (e.g. the delta mass between c11 and c13) indicated the second GalNAc residue is located at S686. The GalNAc residues are denoted as yellow squares according to Consortium for Functional Glycomics (CFG) standard. Source data are provided as a Source Data file.

SARS-CoV-2 is the causative pathogen of COVID-19, a global pandemic that has already claimed over 6.8 million lives worldwide. Unfortunately, there is still no effective strategy to eradicate the virus. It remains crucial to understand the infectious mechanism of the virus and to explore potential strategies to fight viral infection. Compared to the previous epidemic SARS-CoV, a distinct feature of SARS-CoV-2 is the acquisition of a multibasic furin cleavage site at the S1/S2 boundary of its spike protein (S protein), in addition to a shared TMPRSS2 cleavage site at the S2’ position of the fusion peptide^1–4^ (Fig. 1a). Cleavage by host cell proteases at the S1/S2 boundary and/or at the S2’ position has been previously shown to activate MERS-CoV and SARS-CoV by exposing the fusion peptide of the spike protein and induce membrane fusion^5–8^. Therefore, it has been proposed that the enhanced infectivity and pathogenesis of SARS-CoV-2 may be attributed to the dual protease activation mechanism^3,9–13^. While both furin and TMPRSS2 are host cell proteases that cleave the S protein, they work at different stages of SARS-CoV-2’s life cycle^1,13–15^. Furin belongs to the subtilisin-like proprotein convertases (PC) residing in the secretory pathway^16^. The presence of the furin cleavage site in S protein allows SARS-CoV-2 to be pre-activated during virion assembly, while other proteases, such as TMPRSS2 and Cathepsin L, provide additional cleavage and further activation during virion entry^13^. Because human lung tissue has low expression of TMPRSS2 and cathepsin L, it has been demonstrated that furin cleavage is necessary for efficient replication of SARS-CoV-2 in human lung^17^ and the mutant devoid of the furin site has reduced pathogenesis in transgenic mouse models^9^.

Protein *O*-GalNAc type glycosylation (*O*-glycosylation) is a prevalent post-translational modification on membrane and secretory proteins^18^. Unlike *N*-linked glycosylation, which participates in protein folding and quality control in endoplasmic reticulum (ER), *O*-glycosylation is carried out by a consortium of glycosyltransferases located in the Golgi network and fine-tunes a variety of cellular functions, including proteolytic processing^19,20^, ligand binding^21^ and signal transduction^22,23^. As enveloped viruses hijack host cell’s secretory machinery to synthsize viral proteins and, in the case of coronaviruses, to assemble virions, they are inevitably modified by host cell’s glycosylation enzymes. Previous studies have characterized the *O*-glycosylation sites on various viruses, including human immunodeficiency virus (HIV)^24^, Varicella-zoster virus (VZV)^25^, Human Cytomegalovirus (HCMV)^25^, and Epstein-Barr virus (EBV)^25^. Since the outbreak of COVID-19, considerable efforts have been dedicated to profiling both *N*- and *O*-glycosylation of the SARS-CoV-2 spike protein^26–36^. These investigations have revealed a number of *O*-glycosylation sites near the furin cleavage site of SARS-CoV-2 spike proteins. Because *O*-glycosylation is a known mechanism for the cell to regulate protease processing of secretory proteins, an intriguing hypothesis is that host cells could exploit those glycosylation sites to regulate spike protein cleavage by furin^20,37–40^.

Towards this hypothesis, Ten Hagen and colleagues reported that *O*-glycosylation at T678 of S protein, initiated by polypeptide GalNAc-transferase family members (GalNAc-Ts), caused decreased furin cleavage and syncytia formation^41^. A recent study using a chemical probe strategy demonstrated that furin processing is affected by sialylation of the extended *O*-glycan at T678^42^. Therefore, the host cells’ glycosylation machinery could potentially play an important regulatory role in S protein processing and hence viral activation. However, T678 is seven amino acids away from the actual furin cleavage site (after R685), it is still unclear how *O*-glycan at T678 could exert such a long-range effect. The question remains whether other glycosylation sites, rather than T678, are more directly responsible for inhibiting furin processing^34^. Additionally, it is necessary to explore whether the in vitro studies on glycosylation can be extrapolated to understand the biology of authentic SARS-CoV-2 in human lung cells.

In this report, we confirmed a cluster of *O*-glycosylation sites right next to the furin cleavage sequence in SARS-CoV-2 spike protein and discovered that they were catalyzed in tandem by two GalNAc-transferases. We showed the biological impact of the multiple *O*-glycosylations at the furin cleavage site, including regulating the furin processing of the spike protein, the assembly of the S protein into virus-like-particles (VLPs), and the infectivity of authentic SARS-CoV-2 viruses in human lung cells. We also revealed a mechanism by which S protein assembly into VLPs is dependent on furin processing. Overall, our study strongly supports the essentiality of furin cleavage in viral life cycles, proposes a potential mechanism for the assembly of the spike protein into virions, and explains the regulatory role of glycosylation in viral activation and infection. Additionally, we elucidated the impact of SARS-CoV-2 evolution, particularly mutations in the spike protein, on their regulation by host cell glycosylation. Consequently, our findings present a plausible mechanism for the attenuated pathology of Omicron in human lungs. Importantly, the discovery instills optimism for the potential of controlling coronavirus infections by manipulating innate defense mechanisms involving host cell glycosylation.

## Results

### GalNAc-T3 and T7 initiates clustered *O*-glycosylation covering the multibasic cleavage site in S protein of SARS-CoV-2

Previous studies have identified several *O*-glycosylation sites near the multibasic furin site of the spike protein of SARS-CoV-2 using proteins from various sources^26,27,34,43–45^. Among these sites, T676 and T678 have been most frequently identified (Supplementary Data 1). Additionally, S686, located adjacent to R685, where furin cleaves S protein into S1 and S2 subunits, has once been reported to undergo *O*-glycosylation^29^. To gain a comprehensive understanding of the *O*-glycosylation events near the furin cleavage site, we employed the SimpleCell strategy, which simplifies complex *O*-glycan structures to a single GalNAc residue for efficient enrichment by Vicia villosa lectin (VVA) and easier identification by mass spectrometry^25,46–48^. We expressed the extracellular domains of the spike protein with an R685A mutation in 293-F SimpleCell, digested them with chymotrypsin, and enriched the peptide fragments on a 20 cm VVA agarose column (Supplementary Fig. 1a). Using an electron transfer dissociation (ETD)-based LC-MS analysis we identified T678, S686 and S689 as potentially occupied glycosites near the furin site of S protein of SARS-CoV-2 (Supplementary Fig. 1 and 2).

The confirmation of S686 *O*-glycosylation was intriguing due to its proximity to furin cleavage site R685, suggesting that an extended *O*-glycan at this site could potentially impact furin cleavage in the cell. Whereas the identification of multiple *O*-glycosylation events near the furin sites raises the possibility that densely clustered *O*-glycans may influence the efficiency or specificity of furin cleavage during SARS-CoV-2 infection. To investigate the functions of *O*-glycosylations near the furin cleavage site of the spike protein and their biological relevance in viral infection, our initial aim was to identify the specific GalNAc-Ts that are responsible for these modifications. There are in total 20 mammalian GalNAc-T isozymes which are classified into several subfamilies^49^, so we examined the in vitro activity of representative GlaNac-Ts from different subfamilies^49^, including GalNAc-T1, T2, T3, T4, T5, T7, T11, and T18 (Supplementary Fig. 3 and 4), on a synthetic peptide encompassing the multibasic cleavage site sequence of S protein from the original Wuhan strain (Wuhan-Hu-1). Among all the tested GalNAc-Ts, we found that GalNAc-T3 demonstrated the most substantial activity on the synthetic multibasic substrate, adding a single GalNAc to the peptide with almost 100% conversion at the test condition (Fig. 1b). Using ETD-based LC-MS analysis, we determined that GalNAc-T3 specifically modified the residue corresponding to T678 of the spike protein (Fig. 1c). In line with previous studies, our results showed that GalNAc-T1, a “house-keeping” GalNAc transferase, could also modify the furin site-containing peptide in vitro^41,42^, albeit with less efficiency (40.9% at the same test condition).

All GalNAc-Ts, except for GalNAc-T20, possess a distinct lectin domain in addition to their catalytic domain. This characteristic enables them to work in a coordinated manner by recognizing previously glycosylated substrates and creating additional glycosylation sites in close proximity^20,50^. Therefore, we investigated which GalNAc-T might potentially interact with the GalNAc-T3 modified peptide to generate additional glycosylations near the furin site. Interestingly, among all the tested GalNAc-Ts, only GalNAc-T7 readily took the prior glycosylated spike peptide as its substrate, catalyzing the addition of up to two more GalNAc residues to the peptide (Fig. 1d). Using the same LC-MS method, we discovered that GalNAc-T7 mostly modified the residue corresponding to S686 of the spike protein (Fig. 1e), with a minor activity towards the residue corresponding to S689 (Supplementary Fig. 5).

Collectively, in vitro glycosylation results suggested that the activities of GalNAc-T3 and T7 could be responsible for the *O*-glycosylation sites we identified on recombinant spike protein, which were T678, S686 and S689. The two enzymes work in tandem to potentiate clustered *O*-glycan modifications near the furin cleavage site of SARS-CoV-2 S protein. Considering that the glycosylation by GalNAc-T7 at S686 occurs right next to the furin cleavage site and *O*-glycans can undergo further extension by other glycosyltransferases, it has the potential to substantially impede protease processing in the cell. Moreover, by determining the specific GalNAc-Ts and the order of *O*-glycan cluster modifications, we were able to assess the impact of host cell *O*-glycosylation on SARS-CoV-2 S protein in vitro and on authentic viruses in human lung cells, as shown below.

### GalNAc-T3 and T7 inhibit the furin processing of the spike protein

**Fig. 2:**
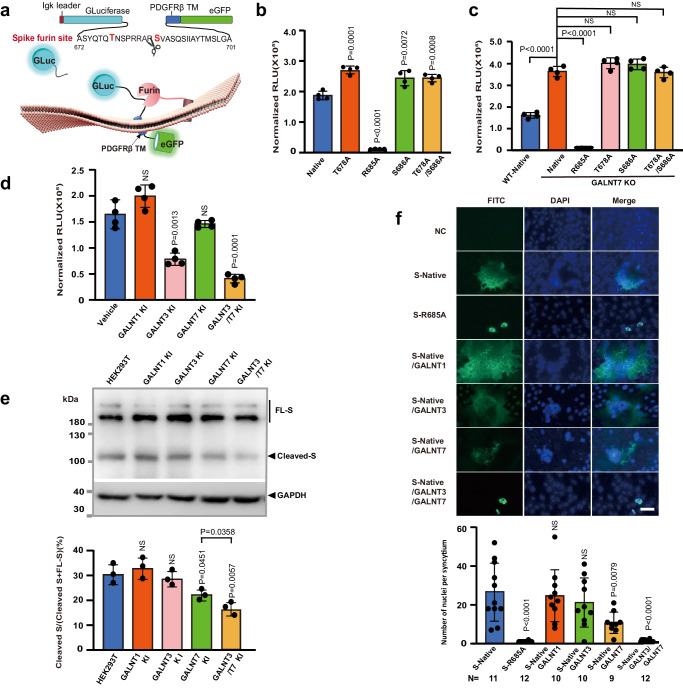
GalNAc-T3 and 7 inhibit furin processing of the spike protein by specific glycosylation at T678 and S686. **a** A schematic diagram of the luciferase-based biosensor assay for furin cleavage. **b** Bioluminescence analysis of furin cleavage in HEK293T cells by biosensors containing the native S1/S2 boundary sequence or glycosite mutations. **c** Bioluminescence analysis of furin cleavage in HEK293T *GALNT7* KO cells by biosensors containing the native S1/S2 boundary sequence or glycosite mutations. **d** Suppression of furin cleavage by GalNAc-T3 and T7 in the biosensor assay. HEK293T cells were co-transfected with the luciferase-based biosensor containing the native S1/S2 boundary sequence and GalNAc-Ts as indicated at the bottom. For (**b**–**d**), Data are presented as mean values ± SD (*n* = 4 independent experiments). **e** Western blot and quantitative analysis of overexpressed spike protein in HEK293T WT and *GALNTs* KI cells. Total protein concentrations in cell lysates were measured by BCA assay and normalized for sample loading. GAPDH was used as a loading control. The results here are representative blots from three independent experiments. Quantitative analysis of Spike processing was performed by measuring the densitometry ratio between Cleaved-S and all forms of full-length S using Image J. Data are presented as mean values ± SD (*n* = 3 independent experiments). **f** GalNAc-T3 and 7 overexpression decreased syncytia formation in Vero E6 cells. Here shows representative views of spike protein-mediated syncytium formation in Vero E6 cells transfected with vector (NC), S-Native, S-R685A, S-Native/GALNT1, S-Native/GALNT3, S-Native/GALNT7 or S-Native/GALNT3/T7 plasmids. Cells were stained with DAPI (blue) and immuno-stained with anti-Flag antibody (green). Syncytia formation was quantified by the number of nuclei per syncytium (FITC^+^ cells containing multiple nuclei). The N values indicate the number of syncytium counted. Data are presented as mean ± SD. *n* = 5 independent experiments. Scale bar, 50 μm. For statistical comparisons between means in data (**b**–**f**), two-tailed *P* values are calculated by unpaired Student’s *t* test. Unless otherwise labeled, the displayed *P* values are the significance between the experimental group and the control group (Native, Vehicle or HEK293T). NS: not significant. Source data are provided as a Source Data file.

In order to test whether *O*-glycosylation initiated by GalNAc-T3 and T7 could inhibit the protease processing at the multibasic cleavage site of S protein in the cell, we first constructed a luciferase-based biosensor, which encodes the S1/S2 boundary sequence sandwiched between a Gaussia luciferase and a membrane-anchored eGFP. Cleavage of the multibasic sequence by furin in HEK293T releases luciferase into the medium, while the membrane-bound eGFP allows normalization of the luminescence signals due to variations in transfection and expression levels (Fig. 2a, and Supplementary Fig. 6). Similar biosensors were used to detect isoform-specific activity of GalNAc-T2 and T3 in previous studies^51^. As demonstrated in Fig. 2b, the biosensor containing the native sequence of the furin cleavage site produced high luminescence signals. However, a single mutation of the residue corresponding to R685 of the spike protein to alanine (R685A), which renders the peptide unrecognizable by furin, significantly reduced the luminescence signal to almost background, confirming that the constructed biosensor is a valid reporter of the protease processing activity in the sequence containing Spike multibasic cleavage site.

Furthermore, the mutation of either T678 or S686 or both to alanine led to an approximately 50% increase in the luminescence signal (Fig. 2b). This suggests that these potential glycosylation sites provide protection for the biosensor from protease cleavage in HEK293T, mostly likely through *O*-glycosylation. Notably, the luminescence increment observed in the double mutant was not significantly different from that of the S686A or T678A mutant. This finding aligns with the dependence of glycosylation at site S686 on prior glycosylation at site T678 as demonstrated in our in vitro enzymatic assays. To confirm the potential role of endogenous T7 in protecting the biosensor from protease cleavage, we measured the luminescence signals from the biosensor and its mutants, specifically T678A and/or S686A, in HEK293T cells with genetic knock-out (KO) of *GALNT7* (Fig. 2c and Supplementary Fig. 7), the gene encoding GalNAc-T7. Indeed, the knock-out of *GALNT7* resulted in a 50% increase in luminescence signal, providing further evidence for the crucial role of T7 in preventing cleavage of the biosensor. Mutations at either T678 or S686 or both glycosylation sites did not further increase the signal, indicating that these two sites protect the biosensor from protease cleavage in WT HEK293T through T7-catalyzed glycosylation. Consistently, co-expression of the biosensor with GalNAc-T3 and T7 resulted in a 78% reduction in luciferase signals in the medium, whereas the suppression observed with GalNAc-T3 or T7 alone is not as significant (Fig. 2d). We speculate that although GalNAc-T3 and T7 are endogenously expressed in HEK293T (Supplementary Fig. 8)^52^, their levels may not be sufficient to fully glycosylate the overexpressed substrate. Together with the in vitro enzymatic assay, the results from the biosensor experiments suggest that sequential glycosylations at the multibasic cleavage site of SARS-CoV-2 S protein initiated by GalNAc-T3 and T7 could inhibit furin cleavage in the cell.

To determine if these findings can be extended to full-length spike protein, we subsequently examined the protease processing of spike protein in HEK293T cells and compared it with single or double mutants in the glycosylation sites. To our surprise, the single mutation of T678A or S686A did not significantly affect the production of S2 fragment from the overexpressed spike protein. However, the double mutation appeared to cause a slight increase in S2 production (Supplementary Fig. 9). As the biosensor results suggested that endogenous expression of GalNAc-T7 may not be sufficient to fully glycosylate the overexpressed furin site sequence, the same could be true for overexpressed full-length S protein. Therefore, we examined the processing of S protein in HEK293T cells with site-specific knock-in (KI) of *GALNT3*, the gene encoding GalNAc-T3, and/or *GALNT7*, aiming to enhance *O*-glycan occupancy (Supplementary Fig. 10). As displayed in Fig. 2e and Supplementary Fig. 11, the knock-in of *GALNT7* alone resulted in about 20% less S2 produced from spike protein processing. And, when both *GALNT3* and *GALNT7* were knocked-in simultaneously, the production of the S2 fragment from the overexpressed spike protein was inhibited by more than 40%. In contrast, the knock-in of either *GALNT3* or *GALNT1* alone did not show any obvious impact on spike protein processing^42^, suggesting that T7-catalyzed glycosylation is likely the rate-limiting step in the multi-glycosylation events and the product of this step conveys the protective function from protease processing.

Because furin cleavage is necessary for the fusion activity of the spike protein of SARS-CoV-2^2,12^, we next tested whether the glycosylation by GalNAc-T3 and T7 could suppress the spike protein-mediated syncytium formation in Vero E6 cells, which has a functional ACE2 receptor. As expected, co-expression of the spike protein with GalNAc-T3 and T7 significantly suppressed the syncytium formation, displaying a phenotype resembling annihilation of the furin site (R685A mutation) (Fig. 2f and Supplementary Fig. 12).

In summary, our biosensor assay and western blot analysis of overexpressed S protein support our in vitro findings that GalNAc-T3 and T7 collaborate to facilitate tandem glycosylations near the multibasic cleavage site of the spike protein. Specifically, the glycosylation at S686 by T7 plays a critical role in preventing protein processing by cellular proteases in HEK293T and inhibiting its fusion activity in the syncytium formation assay. These findings have potential implications for the activation of the spike protein of SARS-CoV-2.

### GalNAc-T3 and T7 inhibit furin-dependent assembly of the virus like particles (VLPs)

Since most enzymes involved in *O*-glycosylation pathway are located in the Golgi apparatus and the coronaviruses assemble in the ER-Golgi intermediate compartment (ERGIC)^53,54^, we were curious about the potential impact of GalNAc-T3 and T7 glycosylation activity on furin cleavage of the spike protein in assembled virions. Therefore, we attempted to assemble virus-like particles (VLPs) of SARS-CoV-2 in HEK293T cells with or without KI of *GALNT3* and/or *GALNT7*. Our VLPs consisted of co-expressed spike protein (S), membrane protein (M), envelop protein (E), and nucleocapsid protein (N). Similar VLPs systems have been previously established to model SARS-CoV^5,55^, MERS-CoV^56^, Ebola^57^, and the recent SARS-CoV-2 virus^58,59^. The utilization of VLPs would allow us to investigate the impact of GalNAc-T3 and T7 mediated furin site *O*-glycosylation on spike protein assembled into virions.

**Fig. 3:**
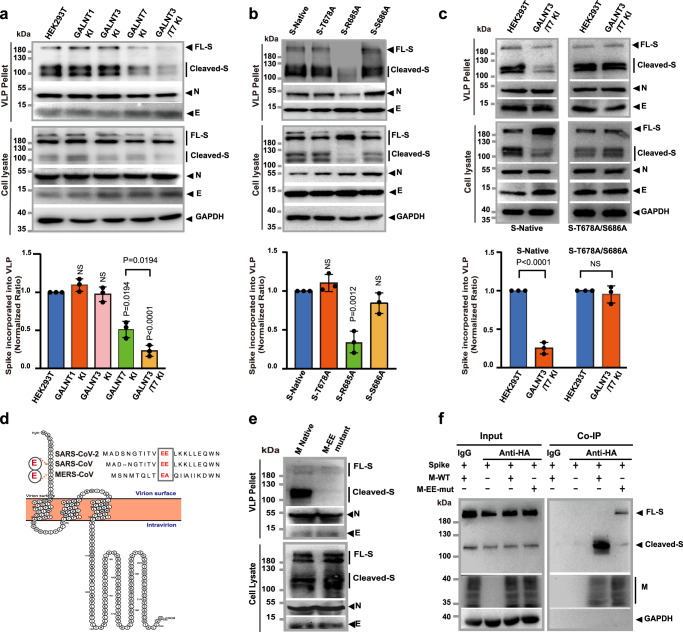
GalNAc-T3 and T7 inhibit furin-dependent assembly of the Virus Like Particles (VLPs). **a** Western blot and quantitative analysis of the assembly and release of SARS-CoV-2 VLPs in HEK293T WT and *GALNTs* KI cells. Different HEK293T cells were co-transfected with native S, together with HA-tagged M, E and N. VLPs and cell lysates were pelleted and analyzed separately. **b** Western blot and quantitative analysis of the assembly of SARS-CoV-2 VLPs with native S protein and mutants in HEK293T WT. The S protein mutant lacking furin site (S-R665A) failed to be incorporated into VLPs. **c** Western blot and quantitative analysis of the assembly of SARS-CoV-2 VLPs with native S protein and T678A/S686A mutant S protein in HEK293T WT and *GALNTs* KI cells. For (**a**, **b**, **c**), results here are representative blots from three independent experiments. Quantitative analysis of S protein incorporation into VLP was performed by calculating the densitometry ratio of S protein (cleaved-S plus FL-S) to N protein, and then normalizing it against the ratio calculated for HEK293T WT (**a**, **c**) or native S (**b**). Data are presented as mean values ± SD (*n* = 3 independent experiments). For statistical comparisons between means, two-tailed *P* values are calculated by unpaired Student’s *t* test. Unless otherwise labeled, the displayed *P* values are the significance between the experimental group and the control group (HEK293T or S-Native). NS not significant. **d** The topology diagram of SARS-CoV-2 M protein. The N-terminal amino acid sequences of M proteins from SARS-CoV-2, SARS-CoV and MERS-CoV are aligned. The EE motif is highlighted by arrowheads. The diagram was generated by Protter^76^. **e** Western blot analysis of the assembly of SARS-CoV-2 VLPs with native M protein and its EE motif mutant in HEK293T WT. **f** Co-immunoprecipitation of S protein with native M protein and its EE motif mutant. Co-immunoprecipitation was performed using anti-HA mAb and examined by western blotting analyses using anti-S1 mAb. IgG were used as a control. For (**e**) and (**f**), the results here are representative blots from three independent experiments. Source data are provided as a Source Data file.

Unexpectedly, double KI of *GALNT3* and *GALNT7* caused an approximately 80% reduction in the amount of spike protein packaged into the VLP pellets, despite no obvious change in the expression level of the spike protein in total cell lysates (Fig. 3a). In contrast, single KI of *GALNT3* or the ubiquitously expressed *GALNT1* did not significantly impact the assembly of spike proteins into VLP, whereas single KI of *GALNT7* also noticeably decreased the amount of incorporated spike protein (by 53%). We noticed that the spike protein incorporated into VLPs was predominantly cleaved, despite the presence of abundant uncleaved spike protein in the cell lysate (Fig. 3a). This finding suggests that the cleaved S is more efficiently incorporated into VLP and furin processing facilitates the assembly of the spike protein into the VLPs of SARS-CoV-2. This hypothesis is supported by the observation that the R685A spike mutant, which lacks the furin cleavage site, was not adequately incorporated into VLPs (Fig. 3b). The results also substantiate the role of GalNacT-3 and T7 in regulating furin cleavage. To further validate the impact of site-specific *O*-glycosylation on furin processing of S protein and its incorporation into VLP, we conducted a similar analysis on the T678A/S686A double mutant of S protein. As depicted in Fig. 3c, the incorporation of the T678A/S686A mutant into VLPs was no longer influenced by the overexpression of GalNAc-T3 and T7. This confirms that the suppression of the spike protein incorporation into VLP by GalNAc-T3 and T7 is potentially through glycosylation at T678 and S686.

Since the outbreak of COVID-19, it has been demonstrated in several studies that furin activation is essential for SARS-CoV-2 infection in the lung^2,9,13,17,60^, however, it is still unclear the exact role of furin-mediated spike protein priming in SARS-CoV-2 infection^9,17,40^. Pseudotyped virions have been routinely used to simulate the protease-dependent activation process of SARS-CoV-2. However, earlier research has indicated that the assembly of SARS-CoV virions relies on the interaction of spike proteins and membrane proteins^55,61^. It is conceivable that similar mechanisms may also be at play in SARS-CoV-2, and pseudotyped virions may not accurately reflect the assembly process of SARS-CoV-2. Indeed, as shown in Supplementary Fig. 13, neither furin site mutation (R685A) nor the double KI of *GALNT3* and *GALNT7* had any impact on the packaging of S protein into the HIV pseudovirus in HEK293T.

We next investigated the possible mechanism underlying the furin-cleavage dependent assembly of the spike protein into SARS-CoV-2 virions. It has been reported that furin cleavage of the spike protein releases multiple arginine residues (RRAR) from the multibasic cleavage site. These positively charged residues are exposed at the loose end of S1 fragment, which triggers an interaction with the host cell receptor neurophilin-1, following the C-end rule^15,62,63^. Previous studies have shown that the interaction between SARS-CoV S protein and M protein is required for viral assembly^54,64^, we therefore hypothesized that the furin-cleaved, multibasic S1 terminal sequence might be involved in the interaction with M protein in the case of SARS-CoV-2. We noticed that the N-terminal, extracellular domain of the SARS-CoV-2 M protein contains an EE motif (Fig. 3d), which could potentially mediate charge-charge interactions with the cleaved S1 fragment. Hence, we introduced alanine mutations to the EE motif of M protein and found that the EE mutant of the M protein failed to incorporate spike protein into VLP (Fig. 3e), mirroring the phenotype produced by R685A mutant of the spike protein with intact M protein. The result suggested that the EE motif of M protein is essential for the assembly of S protein into VLP, possibly via charge-charge interactions with cleaved S. To confirm the specific recognition between S and M protein, we subsequently conducted co-immunoprecipitation experiments with overexpressed S and M proteins in HEK293T. As shown in Fig. 3f, cleaved S protein, but not full-length Spike, could be co-immunoprecipitated with M protein. However, the EE mutant, expressed at a comparable level as native M, could no longer pull down cleaved S (Fig. 3f). These results suggest that the M protein of SARS-CoV-2 specifically interacts with cleaved S protein via a charge-charge interaction between its EE motif and likely the exposed arginine-rich C termini of S1 fragment upon furin cleavage. This interaction is essential for the assembly of functional virions that contain the spike protein.

In addition, to confirm the role that glycosylation plays in the furin-dependant activation of Spike, we performed glyco-analysis on the spike protein isolated from VLP-producing cells. Similar to the results we obtained for overexpressed spike protein, we were able to identify multiple glycosylations on the peptide surrounding the furin cleavage site, including glycosylation on S686 (Supplementary Fig. 14).

In summary, our study using a VLP system that contains the authentic structural proteins of SARS-CoV-2 has yielded significant findings. Firstly, we have discovered that furin cleavage not only activates the S protein but also facilitates its incorporation into the virion. Additionally, we have revealed a potential mechanism for the furin-dependent assembly of virions, which involved a specific interaction between S and M protein. Consequently, the regulation of furin cleavage by GalNAc-T3 and T7 expressed in host cells could excert a substantial influence on virion assembly, and hence the infection caused by SARS-CoV-2.

### Mutations near the multibasic cleavage site alter glycosylation efficiency of GalNAc-T3 and T7

**Fig. 4:**
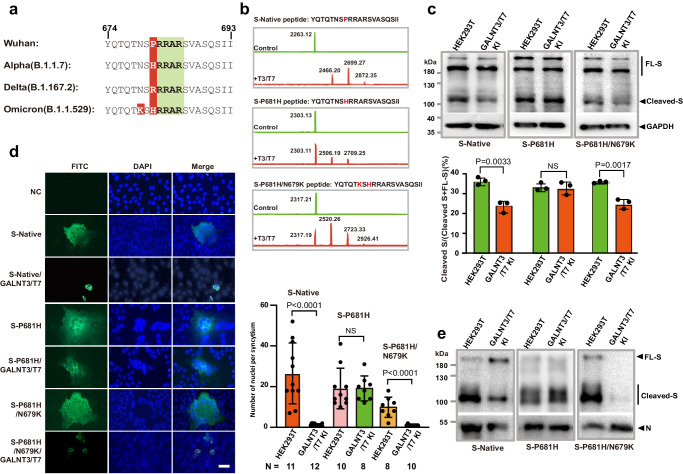
Mutations near glycosylation sites affect the suppression of furin cleavage by GalNAc-T3 and T7. **a** The sequence alignment of spike protein furin site from SARS-CoV-2 variants. **b** MALDI-TOF analysis of GalNAcylation reactions catalyzed by GalNAc-T3 and T7 on the synthetic multibasic substrate of SARS-CoV-2 spike protein (674-693) with P681H and P681H/N679K mutations. Reactions were performed and analyzed as in Fig. 1. **c** Western blot and quantitative analysis of the P681H and P681H/N679K spike protein processing in HEK293T WT and *GALNTs* KI cells. Plasmids encoding native, P681H or P681H/ N679K spike protein were transfected into HEK293T WT and *GALNT3/T7* KI cells as indicated on top of the blot and spike proteins were detected with anti-S2 antibody. The results here are representative blots from three independent experiments. Quantitative analysis of Spike processing was performed by measuring the densitometry ratio between Cleaved-S and all forms of full-length S using Image J. Data are presented as mean values ± SD (*n* = 3 independent experiments). **d** Syncytium formation results of the P681H and P681H/N679K spike protein in Vero E6 cells. Vero E6 cells transfected with vector (NC), S-P681H, S-P681H/GALNT3/T7, S-P681H/N679K or, S-P681H/N679K/GALNT3/T7 plasmids were stained with DAPI (blue) and immuno-stained with anti-Flag antibody (green). The wild-type spike (S-Native) was included as a positive control. Syncytia formation was quantified by the number of nuclei per syncytium (FITC^+^ cells containing multiple nuclei). The *N* values indicate the number of syncytium counted. Data are presented as mean ± SD. *n* = 4 independent experiments. For statistical comparisons between means in data (**c**, **d**), two-tailed *P* values are calculated by unpaired Student’s *t* test. Unless otherwise labeled, the displayed *P* values are the significance between the experimental group and the control group (HEK293T or S-Native). NS: not significant. **e** Western blot analysis of the assembly of SARS-CoV-2 VLPs with P681H or P681H/N679K spike protein in HEK293T WT and *GALNT3/T7* KI cells. VLPs with the native sequence of spike protein were produced in parallel as a control. The results here are representative blots from three independent experiments. Source data are provided as a Source Data file.

As demonstrated in Fig. 4a, three out of the five WHO recognized Variants of Concern (VOC) carry a mutation at proline 681 (P681), three amino acids away from the glycosylation site T678. Among them, the alpha and omicron variants have a histidine (P681H) while the delta variant has an arginine (P681R) at this position. In addition, the omicron variant carries N679K mutation right next to the *O*-glycosylation site T678.

Due to its close proximity, we speculate that P681 mutation could affect the enzymatic activities of GalNAc-T3 and T7 on S protein in vitro. Indeed, as we swapped the P681 residue in our synthetic peptide substrate with histidine (P681H), GalNAc-T3 and T7 could not efficiently glycosylate the peptide, compared to the original peptide with sequence from Wuhan-Hu-1 strain, producing little glycosylated products (Fig. 4b).

However, when the P681H mutation was stacked with N679K mutation, which occurs in Omicron, we surprisingly discovered that GalNAc-T3 and T7 regained significant activity toward the synthetic peptide in the enzymatic assay, producing both mono- and di-GalNAcylated products (Fig. 4b). This result is interesting because together with our findings regarding the glycosylation at the multibasic sequence by GalNAc-T3 and T7 and their inhibition of furin processing of the spike protein from the Wuhan-Hu-1 strain, it suggests that the currently dominant Omicron variant, characterized by both P681H and N679K mutations, could potentially exhibit sensitivity to host cell glycosylation. This same regulatory mechanism may be successfully avoided by previous variants.

To test this hypothesis, we proceeded to evaluate the impact of P681H and N679K mutations on the furin processing of S protein in HEK293T. As demonstrated in Fig. 4c and Supplementary Fig. 15, while expression of GalNAc-T3 and T7 inhibited the production of S2 from overexpressed spike protein carrying the Wuhan-Hu-1 sequence by 35%, the processing of the P681H mutant was not significantly affected. In contrast, the presence of N679K mutation in addition to P681H rendered the processing of the S protein susceptible once again to the inhibitory effects of GalNAc-T3 and T7 (26% reduction). The same trend was observed in the spike protein-mediated syncytium formation assay and the incorporation of S protein into VLP. With the P681H mutant, overexpression of GalNAc-T3 and T7 could neither suppress the spike protein-mediated syncytium formation in Vero E6 (Fig. 4d), nor affect the furin cleavage-facilitated incorporation of the S protein into VLP (Fig. 4e). However, when both P681H and N679K mutations were present, GalNAc-T3 and T7 were able to regain control over the furin processing-dependent functions of S protein (Fig. 4d, e, and Supplementary Fig. 16). These observations are in accordance with the efficiency of the S-P681H/N679K peptide being glycosylated by GalNAc-T3 and T7 as shown in the bottom panel of Fig. 4b.

Taken together, our data suggested that mutations carried by SARS-CoV-2 variants near the multibasic cleavage site have a dramatic impact on the glycosylation efficiency of GalNAc-T3 and T7. Single mutation at P681 carried by earlier variants (alpha and delta) of SARS-CoV-2 could have enabled the virus to resist host cell glycosylation, but the introduction of an additional N679K mutation in the most recent omicron variant could potentially make the virus susceptible to the regulation of host cell glycosylation once more.

### Overexpression of GalNAc-T3 and T7 suppresses omicron in human lung cells

**Fig. 5:**
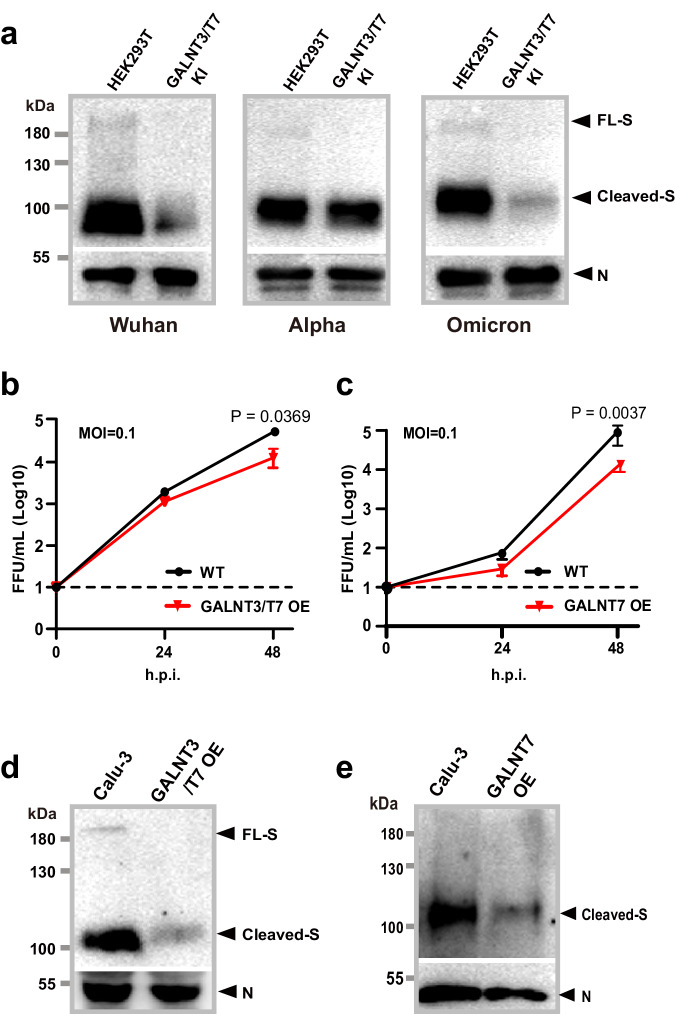
GalNAc-T3 and T7 inhibit replication of the omicron variant of SARS-CoV-2 in Calu-3 cells. **a** Western blot analysis of the assembly of SARS-CoV-2 VLPs with alpha or omicron spike protein in HEK293T WT and *GALNT3/T7* KI cells. VLPs were assembled and detected as in Fig. 3. VLPs with Wuhan spike protein were produced in parallel as a control. **b** Viral titers from Calu-3 cells infected with SARS-CoV-2 omicron variant (BA.1) at an MOI of 0.1, with or without co-expression of GalNAc-T3 and T7. **c** Viral titers from Calu-3 cells infected with SARS-CoV-2 omicron variant (BA.1) at an MOI of 0.1 with or without overexpression of GalNAc-T7. For (**b**, **c**) data are presented as mean values ± SEM (*n* = 3 biologically independent experiments), and two-tailed *P* values are calculated by unpaired Student’s *t* test and displayed above the compared means. **d** Western blot analysis of S protein and N protein of the omicron virions from Calu-3 cells 48 hours post infection (HPI), with or without co-expression of GalNAc-T3 and T7. **e** Western blot analysis of S protein and N protein of the omicron virions from Calu-3 cells 48 h post infection (HPI), with or without overexpression of GalNAc-T7. For data (**a**, **d**, **e**), the results here are representative blots from three independent experiments. Source data are provided as a Source Data file.

In order to investigate whether the evolution of this regulatory mechanism of glycosylation applies to spike proteins from SARS-CoV-2 variants, which contain additional mutations beyond P681 and N679, we first analyzed S protein incorporation into VLP using spike proteins of Wuhan-Hu-1, alpha and omicron variants. Similar to what we showed in the previous section, double KI of *GALNT3* and *GALNT7* significantly decreased the amount of incorporated spike protein from Wuhan-Hu-1 into VLP virions (Figs. 3a, 5a). Likewise, the overexpression of both GalNAc-Ts also resulted in a substantial reduction in the incorporation of omicron S protein into VLP. There was a much lesser extent of reduction observed for alpha S protein. These findings align with our observation that the P681H mutation, present in the alpha S protein, confers resistance to glycosylation at the furin site. Conversely, the omicron variant reverses this phenomenon.

Encouraged by the VLP results and because the omicron variant of SARS-CoV-2 has become one of the dominant strains globally, we were motivated to investigate the susceptibility of the authentic omicron variant of SARS-CoV-2 to the suppression exerted by GalNAc-T3 and GalNAc-T7 in human lung cells. In order to evaluate the impact of GalNAc-Ts on viral replication, we infected Calu-3 cells, a human lung cell line, with an early omicron subvariant BA.1, the original Wuhan-Hu-1 and an alpha subvariant B1.1.7, and measured the viral titer both with or without the overexpression of GalNAc-T3 and T7, either alone or in combination (Supplementary Fig. 17). As shown in Fig. 5b, the replication of the omicron subvariant BA.1 was considerably inhibited by the co-expression of GalNAc-T3 and T7 at a multiplicity of infection (MOI) of 0.1 within the first 24 hours. At 48 hours post-infection (h.p.i.), the viral titer of the omicron virus was reduced by approximately 76% in Calu-3 cells overexpressing GalNAc-T3 and T7, compared to wild-type cells (WT). Remarkably, the expression of GalNAc-T7 alone exhibited a similar degree of reduction in the viral titer, as shown in Fig. 5c. This finding is consistent with the prominent role of GalNAc-T7 in inhibiting the assembly of the spike protein into viral-like particles (VLPs) (Fig. 3a).

To investigate the inhibitory effect of GalNAc-T3 and T7 on S protein processing and subsequent virion assembly during viral infection, we collected the pellet of the omicron virions and analyzed the level of both S and N proteins by western blot. Importantly, we observed that both the co-overexpression of GalNAc-T3 and T7 and the overexpression of GalNAc-T7 alone led to a significant reduction in the amount of S protein incorporated into Omicron virions (Fig. 5d, e). This result mirrored the suppression of the assembly of S protein into VLP by GalNAc-T3 and T7 (Figs. 3a, e). Together with our in vitro glycosylation studies, these results strongly suggest that the inhibition of the omicron virus by GalNAc-T3 and T7 may occur through glycosylation at the furin processing site of the spike protein.

Surprisingly, the replication of the original Wuhan-Hu-1 virus appeared to be less affected by the overexpression of GalNAc-T3 and T7, whereas that of the alpha variant (B1.1.7), which contains P681H mutation, was comparably inhibited by GalNAc-T3 and T7 as that of the omicron variant (Supplementary Fig. 18). Because GalNAc-T3 and T7 demonstrated high enzymatic activity towards the peptide sequence from Spike of the Wuhan-Hu-1 origin and much lower activity towards the sequence harboring P681H only mutation in vitro (Fig. 4b), the results with authentic Wuhan-Hu-1 virus and alpha variant may indicate that the Wuhan-Hu-1 virus was less dependent on furin activation than the newer variants. Nevertheless, it is evident that the glycosylation mediated by GalNAc-T3 and T7 plays an important role in the regulation of viral infection and the current prevalent variant of SARS-CoV-2, Omicron, remains responsive to this regulation. Glycosylation at the multibasic site of the spike protein could be the exploited for future therapeutic interventions.

## Discussion

The spike protein in the envelope of SARS-CoV-2 is densely coated with *N*- and *O*-glycans^26–36^. The roles of *N*-glycan in viral infection have been intensively studied^65,66^, however, the relevance of spike *O*-glycosylation in viral infection is still not clear. Potential *O*-glycosylation sites located near the furin cleavage site of SARS-CoV-2 spike proteins can theoretically confer host cells an inhibitory strategy against furin-based activation of the virus^41^. However, without identifying specific GalNAc transferases involved in modifying the furin site, such a hypothesis cannot be substantiated. In addition, the exact mechanism for furin activation of the spike protein is not entirely known either.

In this report, we show that GalNAc-T3 and T7 together are sufficient to initiate multiple *O*-glycosylations near the furin cleavage site of SARS-CoV-2 spike protein, which inhibit furin processing of the spike protein, suppress the assembly of S protein into VLPs and inhibit the infectivity of the latest omicron variant of SARS-CoV-2 in human lung cells, providing strong evidence for the regulatory role of *O*-glycosylation in viral activation and infection. Importantly, mechanistic studies revealed that S protein incorporation into VLPs is dependent on furin processing and the specific interactions between the furin-cleaved S protein and the M protein of SARS-CoV-2 play an important role in mediating the assembly. Our findings offer a further insight into how furin activates Spike and facilitates the incorporation of the S protein into virions through cleavage. This supports the critical role of furin cleavage in the life cycle of SARS-CoV-2. In addition, our data suggest the virus variants, including B.1.1.7 (alpha) and B.1.167.2 (delta) could have evolved to resist host cell *O*-glycosylation by introducing a mutation at proline 681 (P681) near the furin cleavage site. Interestingly, the omicron variant, which harbors an additional mutation at asparagine 679 (N679), surprisingly reverted this resistance by allowing efficient host cell glycosylation near the furin site of S protein as we show in vitro. Hence, the innate defense mechanism involving host cell glycosylation could provide an explanation for the attenuated pathology of Omicron. It also presents an opportunity for an alternative approach to combat SARS-CoV-2 by manipulating the host glycosylation machinery.

In our study, we demonstrated that two GalNAc transferases, GalNAc-T3 and T7, worked in tandem to create clustered *O*-glycosylation near the multibasic cleavage site of the spike protein of SARS-CoV-2. GalNAc-T1 was reported to glycosylate the synthetic peptide encompassing the furin site of S protein and modulate furin cleavage of the overexpressed spike protein in Drosophila cells^41,42^. There was also evidence showing the effect of T678 *O*-glycosylation on furin processing is dependent on sialylation^42^. In our enzymatic assay, GalNAc-T3 exhibits higher enzymatic activity than GalNAc-T1 towards T678 in the peptide-based in vitro assay. However, neither GalNAc-T1 nor T3 alone is sufficient to make *O*-glycosylation clusters near the furin site of the spike protein without further modifications by GalNAc-T7. As demonstrated in functional assays with VLP and authentic viruses, the activity of GalNAc-T7 exhibits a dominant role in inhibiting furin activation of S protein and viral assembly. Nevertheless, the association between GalNAc-T3 and furin sites is worth further investigation because the modification in SARS-CoV-2 spike protein (**T**_**678**_NSPRRAR**S**) is reminiscent of the natural activity of GalNAc-T3 near the furin proprotein processing site of FGF23 (PRRH**T**_**178**_RS)^20,50^ (Supplementary Fig. 19). It should be pointed out here that GalNAc-Ts are the enzymes that initiate protein *O*-glycosylations, and further extension of *O*-glycans in the cell likely renders the modification better at covering the site from furin processing. Moreover, since furin prefers highly positively charged arginines near the cleavage site, the negative charges from sialylation, which most *O*-glycans in human cells contain^67^, could have an additional impact on furin activity at the site. It is also worth noting that we have observed the major product of GalNAc-T7 on the synthetic peptide to be S686-glycosylated, however, the minor product, S689-glycosylated peptide, was also present. In the glyco-analysis of the overexpressed spike protein and spike purified from VLP-producing cells, S689 was also found to be glycosylated. So it’s likely that GalNAc-T7 can glycosylate both S686 and S689, generating dense *O*-glycans near the furin site.

Surprisingly, we discover that co-expression of GalNAc-T3 and T7 is sufficient to inhibit the assembly of the spike protein into VLPs, similar to abolishing the furin cleavage site. The observation indicates the potential involvement of furin cleavage in virion assembly. It is generally accepted that furin cleavage in the S1/S2 site of the spike protein is essential for viral production, infection, and cytopathic effects of SARS-CoV-2^2,12,14^, and the essentiality has been consistently demonstrated in lung tissue and lung epithelial cell line Calu-3^2,9,13,17,60^. However, furin dependence does not apply to certain cell types including Vero E6^2,9,17,60^ and it remains unclear what determines the reliance on furin activation during SARS-CoV-2 infection in various cell types. Moreover, the intricate mechanism underlying the evolutionary advantage of possessing a furin site remains poorly understood, despite the proposal of several mechanisms. These include the furin cleavage-dependent conformational change of the spike protein to accommodate the ACE2 host cell receptors^10,68^ and the interaction of furin-released S1 C-terminal with host cell receptor neurophilin-1^62,63^. Our discovery of the involvement of furin cleavage in the assembly of spike proteins into VLPs offers an additional perspective on how SARS-CoV-2 takes advantage of the acquired furin site to facilitate virion assembly. Interestingly, previous studies have shown that furin cleavage is partially dispensable for the particle incorporation of SARS-CoV-2 Spike in Calu-3 and does not impact the quantity of Spike incorporated in Vero cells^9,60^. The disparity between our VLP result and the authentic virus may stem from the ability of the authentic virus to replicate. In other words, live viruses might be able to compensate for the inefficienct particle incorporation of uncleaved S protein by upregulating protein expression and other factors.

**Fig. 6:**
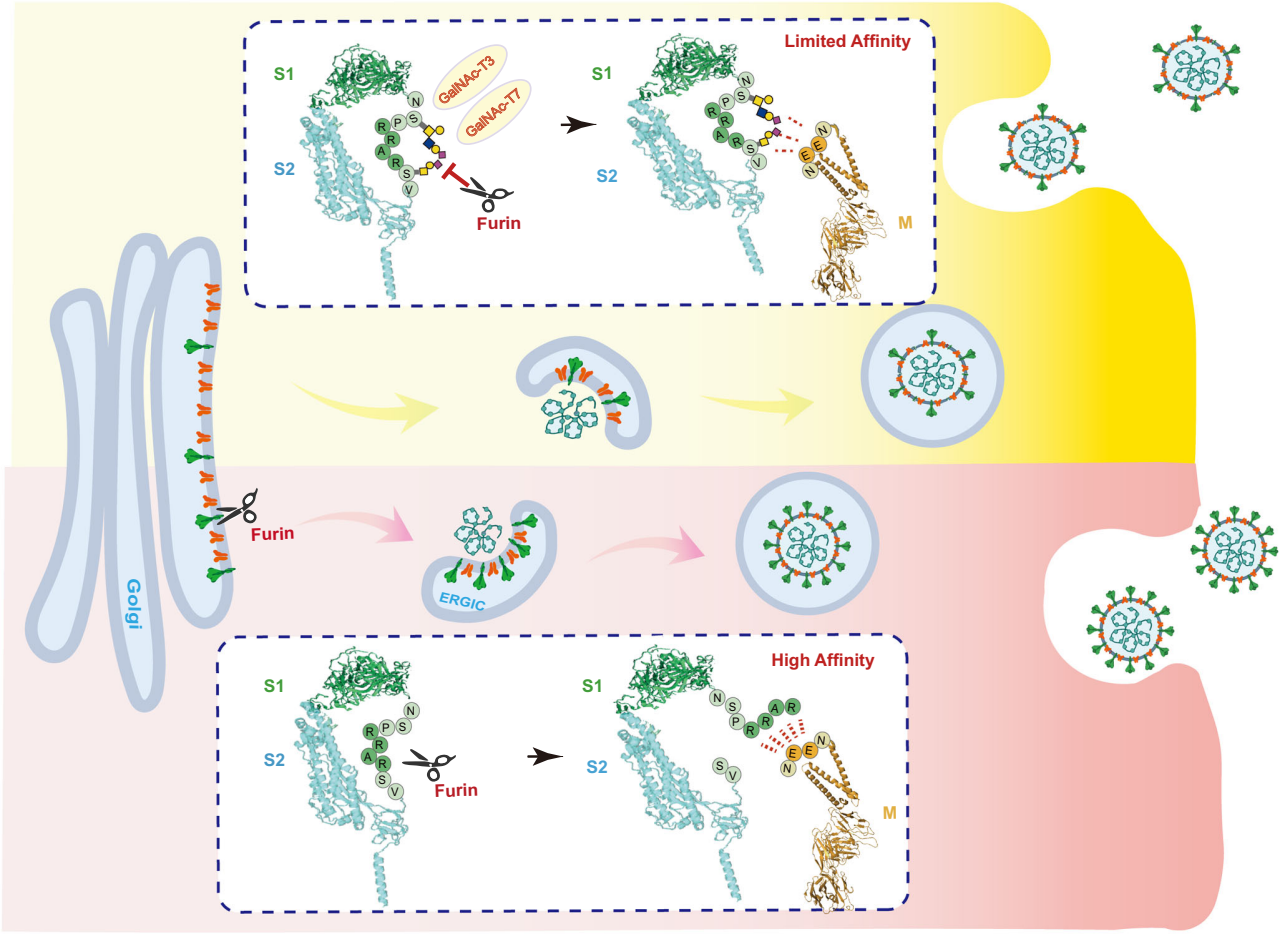
A proposed model for SARS-CoV-2 virion assembly. The assembly of SARS-CoV-2 virion is facilitated by the charge-charge interactions between S1 and Membrane protein, which is mediated by furin cleavage of the spike protein (bottom schematic) and suppressed by O-glycosylation through GalNAc-T3 and T7 (top schematic). The structural models of SARS-CoV-2 Spike and Membrane protein in the schematics were generated using Pymol 2.5 (PDB code 7DDD^77^ and 7VGR^78^, respectively). The Spike is shown in the cartoon representation with S1 colored in green and S2 colored in cyan. The residues encompassing the furin cleavage site in Spike are depicted as single-letter labeled circles with O-glycan modifications denoted according to CFG standards. The Membrane protein of SARS-CoV-2 is also shown in the cartoon representation and colored in brown with residues in the EE motif depicted as single-letter labeled circles. In the assembled virus particles, the Spike and Membrane protein are colored in green and orange, respectively.

VLP represents a simplified system, isolating viral assembly from other steps in the virus life cycle. Nevertheless, it remains valuable for elucidating mechanistic details during virion assembly. Our finding of furin-cleavage facilitated incorporaton of S protein into virion particle is supported by further discovery of an EE motif in the extracellular domain of the M protein, whose absence disrupts the incorporation of cleaved Spike into VLPs. Prior studies have identified M protein as the most important component for virion assembly of a variety of coronavirus (CoVs)^61^ and the interaction of SARS-CoV S protein and M protein is required for the localization of the spike protein to the virus assembly site in ERGIC^54,64^. Therefore, we propose a model (Fig. 6) in which the assembly of SARS-CoV-2 virion is facilitated by the charge-charge interactions between S1 and M protein, which is further mediated by furin cleavage of the spike protein and suppressed by *O*-glycosylation through GalNAc-T3 and T7.

Considering the significance of furin cleavage in the spike protein, we hypothesize that SARS-CoV-2 may undergo natural evolutionary changes to evade suppression by host cell O-glycosylation through the accumulation of mutations in the surrounding region. Indeed, our data suggest that the spike protein containing P681H substitution resists glycosylation by GalNAc-T3 and T7, therefore the variants containing mutations at this position (P681H for alpha and P681R for delta) could have evolved it to resist host cell glycosylation. Surprisingly, however, we have discovered that an additional mutation next to the glycosylation sites, N679K, in the spike protein, as seen in the currently prevalent omicron variant, restores its susceptibility to GalNAc-T3 and T7 glycosylation. Overexpression of GalNAc-T3 and T7 can significantly suppress the furin processing of the spike protein and its incorporation into Omicron virions, leading to 76% reduction in viral titer in human lung cell Calu-3. We have reached the following conclusions based on our findings: 1. The glycosylation of the furin site and the suppression of furin processing by GalNAc-T3 and T7 in vitro also apply to the authentic Omicron virus; 2. The currently prevalent omicron variant seems to revert the resistance to host cell glycosylation that was developed by previous variants. A caveat of this conclusion is that the in vitro activities of GalNAc-T3 and T7 cannot be directly translated to the susceptibility of the original Wuhan virus and the alpha variant to glycosylation. This may be due to the compound effect of many other mutations in spike proteins of these viruses^69^. Due to local legal regulations, we are unable to perform reverse engineering through point mutations on SARS-CoV-2 to study the impact of site-specific glycosylations. As a result, we could not rule out the possibility that the inhibition of authentic SARS-CoV-2 viruses by GalNAc-T3 and T7 is partly influenced by glycosylation sites other than those adjacent to furin processing sites. Nevertheless, recent studies have provided sufficient evidence to suggest that Omicron exhibits lower replication efficiency in the human lung^70^, which could explain its attenuated pathology. Because GalNAc-T3 and T7 are highly expressed in human lung (Supplementary Fig. 20), our discovery provides a plausible mechanism for the regulation of viral infection by host cell glycosylation and establishes a theoretical foundation for the development of potential therapeutic intervention strategies against SARS-CoV-2.

## Methods

### Cell cultures

HEK293T (human, kidney, Cat# CC4003) and Calu-3 (human, lung, Cat# CC0213) cells were purchased from Cellcook Biotech Co., Ltd., Vero E6 (African green monkey, kidney, ATCC: CRL1586) was obtained from the American Type Culture Collection. HEK293T and Vero E6 cells were cultivated in Dulbecco’s Modified Eagle Medium (DMEM, Gibco) supplemented with 10% fetal bovine serum (Gibco), 100 U/mL of penicillin and 0.1 mg/mL of streptomycin (Thermo Fisher Scientific). Calu-3 cells were cultivated in Minimum Essential Medium (MEM, Gibco) supplemented with 20% fetal bovine serum, 100 U/mL of penicillin, and 0.1 mg/mL of streptomycin. FreeStyle^TM^ 293-F cells were purchased from Thermo Fisher Scientific and cultivated in FreeStyle^TM^ 293 medium (Gibco) supplemented with 100 U/mL of penicillin and 0.1 mg/mL of streptomycin. All cell lines were incubated at 37 °C and 5% CO_2_ in a humidified incubator. For seeding and subculturing, cells were first washed with phosphate buffered saline (PBS) and then incubated in the presence of trypsin/EDTA solution (Sigma-Aldrich) until cells were detached. Lipofectamine 3000 (Thermo Fisher Scientific) was used for all transfection experiments according to the manufacturer’s instructions unless otherwise noted.

### Plasmids

To obtain the expression plasmids for SARS-CoV-2 spike protein, we PCR-amplified the coding sequence of a synthetic, codon-optimized SARS-CoV-2 spike DNA based on the publicly available protein sequence (RefSeq no. YP_009724390.1) and cloned into the pcDNA6/V5-His expression vector via *Xho*I and *Age*I restriction sites. Mutations in SARS-CoV-2 spike expression plasmids were generated by overlap extension PCR, using primers listed in Supplementary Data 2. The expression plasmids for Alpha and Omicron variants of SARS-CoV-2 spike protein were generated by cloning the gene sequence encoding Alpha and Omicron spike (Genebank accession no. QUV36347.1 [https://www.ncbi.nlm.nih.gov/protein/QUV36347] and UPX99225.1 respectively) into pcDNA3.1(+)/Flag-His vector via EcoRV and XbaI restriction sites.

To obtain the expression plasmids for GalNAc-Ts, the coding sequences for full length GalNAc-T1 (RefSeq no. NP_001371368.1) and GalNAc-T3 (RefSeq no. NP_004473.2) were codon optimized and synthesized by Tsingke, China. The cDNA encoding GalNAc-T7 (RefSeq no. NP_001362529.1) was purchased from Tsingke, China. Full-length sequences of *GALNT1*, *GALNT3* and *GALNT7* were PCR amplified and inserted into pcDNA3.1/myc-His vector via *BamH*I and *Xho*I restriction sites.

To obtain the expression plasmids for secreted GalNAc-Ts and Spike, we PCR-amplified each gene without the transmembrane domain and cloned it into the pSec-Tag2A expression vector via *Bam*HI and *Xho*I restriction sites. Primers for PCR amplification are listed in Supplementary Data 2.

The plasmid expressing the luciferase-based biosensor was generated by sequentially inserting the genes encoding eGFP, Gaussia luciferase, the linker sequence containing spike furin site (672-701) and PDGFRβ TM domain into pDisplay expression vector (Thermo Fisher Scientific). The gene encoding eGFP was first inserted to pDisplay vector via *Xho*I restriction site. Then the synthesized DNA fragment consisting Gaussia luciferase, the linker sequence and PDGFRβ TM domain was inserted before eGFP via *Sma*I and *Not*I restriction site, resulting in the final plasmid designated as pDisplay-gLuc. The biosensor plasmids with mutations in the linker sequence were obtained by site-directed mutagenesis from pDisplay-gLuc. The primers to generate T678A, P681H, R685A, or S686A mutation in the linker are listed in Supplementary Data 2. Plasmids with desired mutations were confirmed by sequencing.

To obtain expression plasmids for SARS-CoV-2 VLPs, the coding sequences of SARS-CoV-2 M protein (NCBI RefSeq: YP_009724393.1), N protein (NCBI RefSeq: YP_ 009724397.2) and E protein (NCBI RefSeq: YP_009724392.1) were synthesized individually with an HA tag at C-terminal and cloned into pcDNA3.1(+) expression vector via *Bam*HI and *Eco*RI restriction sites. The EE motif mutations in M protein expression plasmids (E11A/E12A) were obtained by site-directed mutagenesis using primers listed in Supplementary Data 2.

To construct an all-in-one gRNA and Cas9 expression vector targeting the AAVS1 site of HEK293T cell, HP180^71^ was linearized with *Bbs*I digestion, and a pair of gRNA oligos (AAVS1-gRNA-F/AAVS1-gRNA-R listed in Supplementary Data 2) were annealed and cloned into linearized HP180 vector, the resulting vector was named HP180-AAVS1-gRNA.

An HMEJ donor for genomic AAVS1 loci knock-in of *GALNTs* was constructed using In-fusion cloning as described by Hui Yang, et al.^71^. In brief, the AAVS1 left (802 bp) and right (837 bp) homology arms with AAVS1-gRNA targeting sequence and an *Eco*RI restriction site were PCR amplified and ligated with AmpR DNA sequence and ori DNA sequence amplified from pCDNA3.1 vector to generated AAVS1-HA donor vector, followed by insertion of SV40 promoter-puroR-β -globin poly(A) signal cassette, 2× CHS4 insulator and GALNTs-Myc sequence in the *Eco*RI site to generate AAVS1-GALNT*s*-KI donor vectors. All newly generated vectors were validated by DNA sequencing.

### Expression and purification of GalNAc-Ts

To obtain purified GalNAc-Ts, the expression plasmids for secreted GalNAc-Ts were transfected into FreeStyle^TM^ 293-F cells using FectoPRO® transfection reagent (Polyplus). The supernatant was collected 72 hours post transfection by centrifugation at 4000 x g for 10 min and then dialyzed against buffer containing 50 mM Tris (pH 8.0) and 150 mM NaCl at 4 °C overnight. Ni-NTA chromatography was performed by first loading dialyzed sample onto Ni-NTA column (Invitrogen, Carlsbad, CA, USA), then washing with 10–20 CV of buffer containing 25 mM Tris (pH 8.0), 300 mM NaCl and 10 mM Imidazole, and eluting with 5 CV of buffer containing 25 mM Tris (pH 8.0), 300 mM NaCl and 250 mM Imidazole. Collected elutes were analyzed by SDS-PAGE (10% Tris-glycine gel) and Coomassie staining.

### The in vitro GalNAc-T activity assay

The peptides from the multibasic site of SARS-CoV-2 spike protein (674-693) and its variants were custom synthesized by GenScript and used as substrates for the in vitro GalNAc-T activity assay. The reactions were carried out at 37 °C in a final volume of 25 μL containing 25 mM sodium cacodylic acid (pH 7.4), 10 mM MnCl_2_, 0.25% Triton X-100, 2 mM UDP-GalNAc, 10 μg of substrate peptide and initiated by adding 0.1 μg of individually purified GalNAc-Ts. The reactions were quenched at 4 h by adding 100 μL 1% TFA and subjected to MALDI-TOF and ETD based LC-MS/MS analysis as described below. A Muc1-derived peptide (HGVTSAPDTRPAPGSTAPPA) was used as the positive control.

### Sample preparation and LC-MS analysis

The extracellular domains of Spike were expressed and purified the same as GalNAc-Ts except FreeStyle™ 293-F SimpleCell were used for expression^47,48^. The full-length Spike protein was obtained by immunopurification from the VLP-producing FreeStyle™ 293-F SimpleCell 72 h after co-transfection with plasmids encoding FLAG-S, HA-M, HA-E and HA-N proteins. Cells were collected and lysed with co-IP lysis buffer (50 mM Tris-HCl, pH 7.4, 150 mM NaCl, 0.5% CA-630, 1 mM PMSF, 1 mM Na_3_VO_4_, 1X protease inhibitor cocktail). Anti-FLAG Magnetic Agarose (cat# A36797, Thermo Fisher Scientific) was used for immunopurification with 50 μL Magnetic Agarose solution in 1 mL cell lysate (1 μg/μL) and the protein was eluted in 50 mM Tris-HCl, pH 7.4, 10 mM DTT, 0.5% Rapigest.

The purified protein was buffer exchanged to 50 mM ammonium bicarbonate and reduced with 10 mM DTT for 45 min at 60 °C followed by alkylation with 20 mM iodoacetamide for 45 min in the dark. The protein was digested with 1:100 chymotrypsin-protein ratio at 37 °C for 12 hours. After digestion, the peptides were treated with PNGase F at 37 °C for 8 h in 100 mM Tris HCl (pH 8) and neuraminidase at 37 °C for 12 h in 50 mM NaOAc (pH 4.5). The glycopeptides were enriched using a 20 cm-long VVA agarose column as described^72^ and desalted by a Stage-Tip.

For LC-MS analysis, one sample for extracellular domains of Spike and one sample for full-length Spike were analyzed (*n* = 1) using an Easy-nLC 1200 system coupled to an Orbitrap Fusion Tribrid Mass Spectrometer equipped with a Nanospray Flex^TM^ ion source (Thermo Fisher Scientific). Samples were separated on a single analytical column, packed in house with ReproSil-Pur-AQ C18 phase (Dr. Maisch, 1.9 μm particle size, 20 cm column length), at a flow rate of 300 nL/min. Samples were dissolved in 0.1% FA, injected onto the column and eluted in a 120 min gradient from 3% to 32% mobile phase B (mobile phase A: water with 0.1% FA. mobile phase B: 80% acetonitrile, 0.1% FA, and 19.9% water). The Nanospray ion source was operated at 2.2 kV spray voltage and 275 °C heated capillary temperature. The mass spectrometer was set to acquire full scan MS spectra (350–1700 *m/z*) for a maximum injection time of 100 ms at a mass resolution of 50,000 and an automated gain control (AGC) target value of 5e5. The MS^2^ analyses were performed in the positive ion mode using data-dependent acquisition with dynamic exclusion set to 60 s at exclusion window of 10 ppm. Top 10 most abundant multiply charged precursors from the full scan were selected for fragmentation via electron-transfer/collision-induced dissociation (ETciD) in the orbitrap at 50,000 resolution. The ETciD experiment was carried out with calibrated charge dependent ETD parameters and a SA collision energy of 30%. The AGC target was set to 5.0e4 and the maximum injection time was 75 ms.

For the site-specific glycopeptide identification, MS/MS spectra were searched against SARS-CoV-2 wuhan-1 Spike sequence (UniProt ID P0DTC2) using Proteome Discoverer 2.5 software with Sequest HT as the searching engine. Chymotrypsin was set as the specific proteolytic enzyme with up to three missed cleavages allowed. Carbamidomethylation at cysteine was used as fixed modification and oxidation at methionine, deamidation at asparagine, and HexNAc at serine/threonine were used as variable modifications. Precursor mass tolerance was set to 10 ppm and fragment ion mass tolerance was set to 0.02 Da. All spectra of interest were manually inspected for correct peptide identification and glycosite localization.

### Genetic knock-out of *GALNT7* in HEK293T

The gRNAs targeting exon 6 of *GALNT7* were designed using gUIDEbook^TM^ and cloned into the gRNA/Cas9 dual expression vector pX458 (Addgene plasmid no.48138 [https://www.addgene.org/48138/]). The gRNA containing plasmids were then transfected into HEK293T and single cell-derived knock-out clones were selected as described^73^. In brief, cells were first bulk sorted by flow cytometry 48 h post transfection and GPF-positive cells with medium fluorescence intensity were collected. After 1 week of culturing, cells were seeded as single cells in 96-well plates by limiting dilution. Clones with frameshift mutations were identified by Indel Detection by Amplicon Analysis^73^ using the following primers: GALNT7-KO-F/GALNT7-KO-R listed in Supplementary Data 2. Mutations were confirmed by Sanger sequencing at the target site.

### Site-specific knock-in of *GALNTs* in HEK293T

Single cell-derived knock-in clones were generated as described^71^. In brief, ~70% confluent HEK293T cells in 6-well plates were co-transfected with an AAVS1-GALNT-KI donor vector and HP180-AAVS1-G3 in a ratio of 3:1. Puromycin was added to a final concentration of 100 µg/mL 48 h post transfection and cells were seeded as single cells into 96 well plates after 5 ~ 7 days of puromycin selection. Positive clones were selected according to PCR results. The anti-GALNT1 antibody (cat# HPA012628, Sigma-Aldrich, 1:1000 dilution), anti-GALNT3 antibody (cat# HPA007613, Sigma-Aldrich, 1:1000 dilution) and anti-GALNT7 antibody (cat# PA064243, Sigma-Aldrich, 1:1000 dilution) were used an the the primary antibody and the goat anti-rabbit, HRP-congugated antibody (Cat# 7074, Cell Signaling Technology, 1:3000 dilution) was used as the secondary antibody for the detection of GalNAc-T1, GalNAc-T3 and GalNAc-T7, respectively. The anti-myc antibody (cat# Ab32, Abcam, 1:2000 dilution) and goat anti-mouse, HRP-congugated antibody (Cat# 31430, Invitrogen, 1:3000 dilution) were used as the primary and secondary antibody for the detection of myc-tag expression.

### The furin cleavage assay with luciferase-based biosensor

4.5 × 10^4^ HEK293T cells were seeded into 96-well plates with 200 μL growth medium. Cells were transfected with pDisplay-gLuc and expression plasmids for GalNAc-Ts at a ratio of 1:9 one day after seeding. 5 μL culture medium was collected 24 h post transfection and mixed thoroughly with 60 μL of 16 ng/mL coelenterazine. Bioluminescence signals were measured immediately on a GloMax® Microplate Luminometer. Meanwhile, HEK293T cells were trypsinized and washed twice with ice-cold PBS. Cells were resuspended and analyzed by flow cytometry, following the same gating process illustrated in Supplementary Fig. 6. Mean fluorescence intensities (MFI) of GFP were used to normalize the bioluminescence signals obtained from the medium.

### Immunofluorescence microscopy

Vero E6 cells were washed with PBS and then fixed with 4% paraformaldehyde for 10 min at room temperature. After three washes with PBS, cells were permeabilized with 0.1% Triton X-100 at 4 °C for 10 min. Next, cells were incubated in the blocking buffer (1X PBS, 5%BSA and 0.1% Triton X-100) at room temperature for 30 min. Cells were then incubated with the primary antibody (mouse anti-Flag antibody, Cat# F1804, Sigma-Aldrich, 1:1000 dilution) in the blocking buffer overnight at 4 °C. FITC-coupled anti-mouse antibody (Cat# A16079, Thermo Fisher Scientific, 1:1000 dilution) or 4’,6-diamidino-2-phenylindole (DAPI) nuclear counterstaining was applied for 2 h at room temperature. Images were acquired under a Zeiss AXIO Imager A1 microscope.

### Production of SARS-CoV-2 VLPs

HEK293T cells cultured to 70% confluence in T-75 flasks were co-transfected with plasmids encoding the SARS-CoV-2 S, M, E and N proteins in a molar ratio of 8:6:8:3 using lipofectamine 3000 and Opti-MEM reduced serum medium (Gibco). The medium and cells were collected separately 60 hours post transfection. 10 mL of medium was filtered through a 0.45 µm syringe filter and then centrifuged with 30% sucrose at 111,000 × *g* for 2 h at 4 °C. The final pellet was washed twice with ice-cold PBS, resuspended in 100 µL of 1 x SDS loading buffer (Beyotime), and sonicated in an ice-water bath for 10 min. Meanwhile, cells were washed twice with ice-cold PBS and lysed with RIPA buffer (Beyotime) with protease inhibitor cocktail (MCE), followed by centrifugation at 4000 × *g* for 10 min at 4 °C.

### Production of pseudotyped virus

HEK293T cells were co-transfected with 60 μg of plasmid encoding Env-defective, luciferase-expressing HIV-1 (pNL4-3.luc.RE, Addgene #101342 [https://www.addgene.org/101342/]) and 20 μg of plasmid encoding SARS-CoV-2 spike protein into a 15 cm cell culture dish. The supernatant was harvested 72 h post transfection and centrifuged at 4000 x g. 10 mL of medium was filtered through a 0.45 µm syringe filter and then centrifuged with 30% sucrose at 111,000 × *g* for 2 h at 4 °C. The final pellet was washed twice with ice-cold PBS, resuspended in 100 µL of 1 x SDS loading buffer, and sonicated in an ice-water bath for 10 min.

### Co-immunoprecipitation

FLAG-Spike was expressed alone or co-expressed with HA-M or HA-M -EE- mutant protein in HEK293T cells. Cells were harvested at 48 h after transfection by centrifugation at 4000 x g and lysed with co-IP lysis buffer. Total protein concentrations in cell lysates were measured by BCA assay and normalized 2 µg/µL for sample loading. Protein A MagBeads were mixed with HA tag polyclonal antibody (Cat# 51064-2-AP, Proteintech, 1:100 dilution) solution and incubated for 4 h at 4 °C. The Protein A MagBeads antibody mixture was pre-washed with co-IP lysis buffer before the addition of cell lysates and incubated overnight at 4 °C. After washing the beads with co-IP lysis buffer, the immunocomplexes were eluted using SDS-PAGE sample loading buffer and subjected to western blot analysis using SARS-CoV-2 Spike S1 antibody (Cat# GTX635654, GeneTex, 1:1000 dilution) and anti-mouse, HRP-conjugated antibody (Cat# 31430, Invitrogen,1:3000 dilution) as the primary and secondary antibody for the detection of S protein and S2 cleavage products.

### Viral infection of SARS-CoV-2 in Calu-3 cells

The SARS-CoV-2 variants, including Wuhan-Hu-1^74^, Alpha (B.1.1.7), and Omicron (BA.1) were isolated from COVID-19 patients and preserved in Guangzhou Customs District Technology Center BSL-3 Laboratory. All viruses were propagated in Vero E6 cells and purified by plaque assay. The genome sequences of the purified viruses were obtained by next-generation sequencing and were assigned as Wuhan-Hu-1 (GenBank accession no. MT123290), Alpha (B.1.1.7) and Omicron (BA.1) on the phylogenetic tree by sequencing analysis on the website https://clades.nextstrain.org/. Wild type Calu-3 cells, GalNAc-T7 overexpressing and GalNAc-T3/T7 co-expressing Calu cells were infected with SARS-CoV-2 wuhan-hu-1, alpha variant B.1.1.7 or omicron variant BA.1 at an MOI of 0.1 or 0.01. GalNAc-T3/T7 overexpressing cells were generated by transducing Calu-3 cells with an adenoviral vector containing the expression cassette for GalNAc-T3 and T7 (Ad5-GALNT3/T7 from WZ Biosciences, Jinan city, China) at a multiplicity of infection (MOI) = 1000 for 4 h at 37 °C. GalNAc-T7 overexpressing cells were generated by transducing Calu-3 cells with a lentiviral vector containing the expression cassette for GalNAc-T7, followed by selection with 8 µg/mL puromycin. Calu-3 cells transduced with empty vectors (Ad5-EV or lenti-EV) were used as negative controls. Then, SARS-CoV-2 viruses were inoculated into Calu-3 cells at 37 °C for 1 h. Inocula were then removed before adding 500 μL pre-warmed DMEM per well. Virus titer quantitation was performed by a focus forming assay (see below) at the indicated time points. Virions were pelleted from the culture after the last time point and analyzed for both S and N proteins by western blot. All work with SARS-CoV-2 virus were conducted in the Biosafety Level 3 (BSL3) Laboratories of Guangzhou Customs District Technology Center.

### Focus forming assay

Vero E6 cells were seeded in 96-well plates one day before infection. Virus culture were inoculated into Vero E6 cells in serial dilutions at 37 °C for 1 h. Inocula were then removed before adding 100 μL 1.6% carboxymethylcellulose (pre-warmed to 37 °C) per well. After 24 h, cells were fixed with 4% paraformaldehyde and permeabilized with 0.2% Triton X-100. Cells were then incubated with a rabbit anti-SARS-CoV-2 nucleocapsid protein polyclonal antibody (Cat# A02050, GenScript, 1:2000 dilution), followed by an HRP-labeled goat anti-rabbit secondary antibody (Cat# 7074, Cell Signaling Technology, 1:3000 dilution). The foci were visualized by TrueBlue Peroxidase Substrate (KPL, Gaithersburg, MD), and counted with an ELISPOT reader (Cellular Technology Ltd. Cleveland, OH). Viral titers were calculated as focus forming unit per mL (FFU/mL).

### Western blot analysis

To analyze spike protein processing in cells, HEK293T cells were transfected with the expression plasmid for SARS-CoV-2 spike protein or its mutants. Cells were trypsinized at 48 h post transfection and washed twice with PBS before being lysed with RIPA buffer and protease inhibitor cocktail. Total protein concentrations in cell lysates were measured by BCA assay and normalized for sample loading. Cell lysates were separated on 10% Tris-Glycine SDS-PAGE gels and transferred to polyvinylidene difluoride (PVDF) membranes in a BioRad Mini Trans-Blot cell. The membranes were subsequently blocked with 5% non-fat milk in 1X TBST buffer for 1 h at room temperature prior to incubation with primary antibodies at 4 °C overnight. After washing with 1X TBST for three times, membranes were incubated with HRP-conjugated secondary antibody for 2 h at room temperature. SuperSignal™ West Pico PLUS (Thermo Fisher Scientific) was used for immunoblot imaging. Anti-S2 antibody (Cat# 40590-D001, Sino Biological, 1:1000 dilution) and the rabbit anti-Human, HRP-conjugated antibody (Cat# Ab6759, Abcam, 1;2000 dilution) were used as the primary and secondary antibody, respectively, for the detection of full-length S protein and S2 cleavage products. GAPDH was detected by anti-GAPDH antibody (Cat# 10494-1-AP, Proteintech, 1:2500 dilution) and the goat anti-rabbit, HRP-conjugated antibody (Cat# 7074, Cell Signaling Technology, 1:3000 dilution) as the primary and secondary antibody, repectively.

To analyze spike protein assembly into VLPs and virions, pellet and cell lysate samples were prepared as described above. Spike proteins were detected with anti-S2 antibody (Cat# 40590-D001, Sino Biological, 1:1000 dilution) and anti-N antibody (Cat# A02050, GenScript, 1:2000 dilution) after being separated on a 10% Tris-Glycine gel. E protein in VLPs was detected with anti-HA antibody as the primary antibody (Cat# 2999 S, Cell Signaling Technology, 1:1000 dilution) and the goat anti-mouse HRP-conjugated antibody as the secondary antibody (Cat# 31430, Invitrogen,1:3000 dilution) after being separated on a 15% Tris-Glycine gel.

To analyze spike protein assembly into pseudovirus, pellet samples were prepared as described above. Anti-S2 antibody (Cat# 40590-D001, Sino Biological, 1:1000 dilution) was used as the primary antibody for full-length S protein and S2 cleavage products detection. Primary antibody for HIV1-P24 was purchased from Abcam (Cat# ab9071, Abcam, 1:1000 dilution).

### Quantification and statistical analysis

Biochemical experiments in vitro were routinely repeated at least three times. All western blot experiments with quantification were performed a minimum of three times with biologically independent samples and analyzed by Image J. Statistical analysis was performed and two-tailed *P* values were calculated using unpaired Student *t* test by GraphPad Prism 7 unless otherwise specified. All tests and *P* values are provided in the corresponding figures or figure legends.

### Reporting summary

Further information on research design is available in the Nature Portfolio Reporting Summary linked to this article.

### Supplementary information

Supplementary Information

Peer Review File

Description of Additional Supplementary Files

Supplementary Data 1

Supplementary Data 2

Reporting Summary

### Source data

Source Data

## Data Availability

All data supporting the findings of this study are available within the paper and its Supplementary Information. The mass spectrometry proteomics data have been deposited to the ProteomeXchange Consortium via the PRlDE partner repository^75^ with the dataset identifier PXD049110. The protein sequence of SARS-COV-2 Spike in the UniProt database with ID P0DTC2 was used as a reference for mass spectrometry database search. The structural models of the Spike protein and Membrane protein were downloaded from Protein Data Bank with PDB ID 7DDD and 7VGR. The data for GALNTs expression level were obtained from the Gene Expression Omnibus database with accession numbers GSE216397 for HEK293T cells, GSE176393 for Calu-3 cells and GSE165955 for Vero-E6 cells. The data for GALNTs expression level in human lung were obtained from ArrayExpress with accession number E-MTAB-513937 [http://www.ebi.ac.uk/biostudies/arrayexpress/studies/E-MTAB-513/]. The sequences of Alpha and Omicron Spike can be found with accession number QUV36347.1 and UPX99225.1. The sequences of S, M, N, E, GalNAc-T1, GalNAc-T3 and GalNAc-T7 proteins can be found with RefSeq number YP_009724390.1 for S, RefSeq number YP_009724393.1 for M, RefSeq number YP_ 009724397.2 for N, RefSeq number YP_009724392.1 for E, RefSeq number NP_001371368.1 for GalNAc-T1, RefSeq number NP_004473.2 for GalNAc-T3 and RefSeq number NP_001362529.1 for GalNAc-T7. The sequencing data for the Wuhan-hu-1 strain of SARS-CoV-2 can be found in GenBank with accession number MT123290952 [https://www.ncbi.nlm.nih.gov/nuccore/MT123290]. The sequencing data for alpha strain (B.1.1.7) and omicron strain (BA.1) used in this study are available in the Source Data file. Source data are provided with this paper.
