# Supplementary material for: Sequential glycosylations at the multibasic cleavage site of SARS-CoV-2 spike protein regulate viral activity
Source: Nat Commun. 2024 May 16;15:4162. doi: 10.1038/s41467-024-48503-x (PMC11099032; doi:10.1038/s41467-024-48503-x)
Supplement: Supplementary file 1 — Supplementary Information [file 41467_2024_48503_MOESM1_ESM.pdf]

Supplementary Information for

Sequential Glycosylations at the Multibasic Cleavage Site of SARS-CoV-2 Spike Protein Regulate Viral Activity

Shengjun Wang[1, 14, #], Wei Ran[2, #], Lingyu Sun[1, #], Qingchi Fan[1], Yuanqi Zhao[1,15], Bowen Wang[3], Jinghong Yang[2], Yuqi He[1], Ying Wu[1], Yuanyuan Wang[4], Luoyi Chen[1], Arpaporn Chuchuay[1], Yuyu You[1], Xinhai Zhu[5], Xiaojuan Wang[6], Ye Chen[7], Yanqun Wang[2], Yao-Qing Chen[4], Yanqiu Yuan[8, *], Jincun Zhao[2, 9, 10,11,12, *], Yang Mao[1, 13, *]

*Correspondence: Yanqiu Yuan, Jincun Zhao, Yang Mao

Email: yuanyq8@mail.sysu.edu.cn (Y.Yuan), zhaojincun@gird.cn (J.Z.), maoyang3@mail.sysu.edu.cn (Y.M.)

Supplementary Information Contents:

Supplementary Fig. 1: *O*-glycosites identification of recombinant ECD of Spike.

Supplementary Fig. 2: Graphic depiction of identified *O*-glycosylation sites in the SARS-CoV-2 spike protein in this study.

Supplementary Fig. 3: Purified GalNAc-Ts analyzed by SDS-PAGE and Coomassie staining.

Supplementary Fig. 4: MALDI-TOF analysis of GalNAcylation reactions catalyzed by purified GalNAc-Ts on a synthetic Muc1-derived peptide (HGVTSAPDTRPAPGSTAPPA).

Supplementary Fig. 5: LC-MS analysis of doubly-glycosylated peptide products of GalNAc-T3 and T7 reactions.

Supplementary Fig. 6: The gating strategy for the flow cytometry analysis of GFP expression in the luciferase-based biosensor assay.

Supplementary Fig. 7: CRISPR-based knockout (KO) of *GALNT7* in HEK293T cells.

Supplementary Fig. 8: *GALNTs* expression levels in HEK293T, Calu-3 and Vero-E6 cells from RNA-seq analysis.

Supplementary Fig. 9: Western blot and quantitative analysis of overexpressed spike protein with glycosite mutations in HEK293T WT and *GALNT7* KO cells.

Supplementary Fig. 10: Validation of GALNT KI in HEK293T cells.

Supplementary Fig. 11: The replicate results of western blot analysis of the processing of spike protein overexpressed in HEK293T WT and *GALNTs* KI cells.

Supplementary Fig. 12: *GALNT3/7* expression decreased syncytia formation.

Supplementary Fig. 13: Western blot analysis of S protein packaging into the HIV pseudovirus in HEK293T WT and GALNT3/T7 KI cells.

Supplementary Fig. 14: *O*-glycosites identification of full length Spike from cell lysate after VLP packaging.

Supplementary Fig. 15 The replicate results of western blot analysis of the P681H and P681H/N679K spike protein processing in HEK293T WT and *GALNTs* KI cells.

Supplementary Fig. 16: *GALNT3/7* expression decreases syncytia formation in S-P681H/N679K

Supplementary Fig. 17: Validation of *GALNT3* and/or *GALNT7* overexpression in Calu-3 cells.

Supplementary Fig. 18: GalNAc-T3 and T7 Inhibit the Replication of SARS-CoV-2 in Calu-3 Cells.

Supplementary Fig. 19: Sequential glycosylation near the furin site of FGF23 and SARS-CoV-2 spike protein.

Supplementary Fig. 20: GalNAc-Ts expression in human lung.

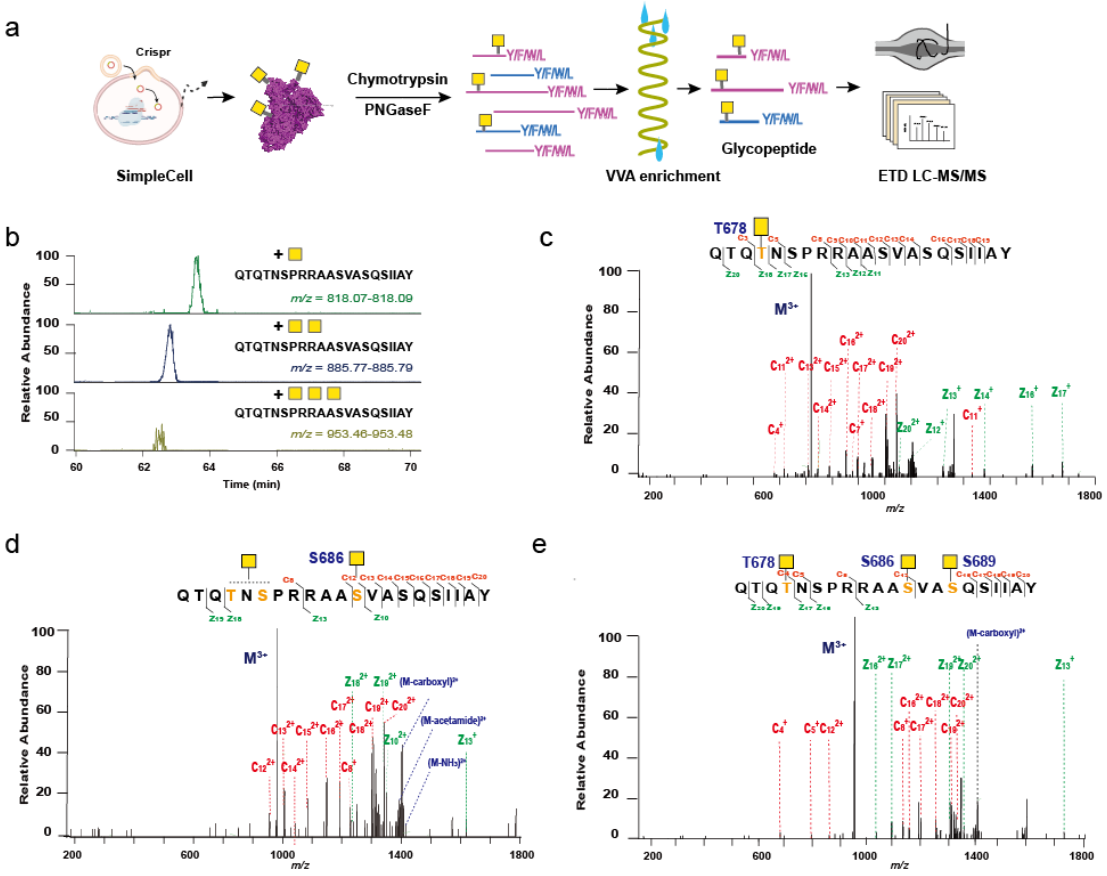

Supplementary Fig. 1 *O*-glycosites identification of recombinant ECD of Spike. a, Workflow for the bottom-up glyco-analysis of recombinant Spike extracellular domain (ECD). FreeStyle™ 293-F was engineered by *C1GALT1* gene knockout to produce truncated *O*-glycans and VVA lectin was used for glycopeptide enrichment[1-3]. MS analysis was performed as described in the method with one sample for extracellular domains of the Spike (n = 1). The structural models of SARS-CoV-2 Spike protein were generated using Pymol 2.5 (PDB code 7DDD[4] [https://doi.org/10.2210/pdb7DDD/pdb]) **b,** Extracted ions for naked and *O*-GalNAcylated peptides after VVA lectin enrichment. The GalNAc residues are denoted as yellow squares according to Consortium for Functional Glycomics (CFG) standard. **c, d** and **e,** ETD-MS^2 spectrum of *O*-GalNAcylated peptides from Spike R685A mutant. The mass of c- and z- fragment ions indicated that T678, S686, S689 were occupied by *O*-GalNAc.

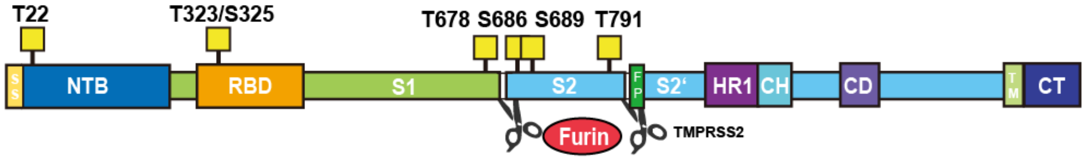

Supplementary Fig. 2 Graphic depiction of identified *O*-glycosylation sites in the SARS-CoV-2 spike protein in this study. Mutant spike protein (R685A) ECD were recombinantly expressed in FreeStyle™ 293-F SimpleCell[2, 3] and subjected to VVA lectin enrichment before being subjected to ETD-based LC-MS analysis. A total of 6 *O*-glycosylation sites were identified, including T22, T323/S325, T678, S686, S689 and T791. The GlaNAc was shown as filled yellow squares in the graph.

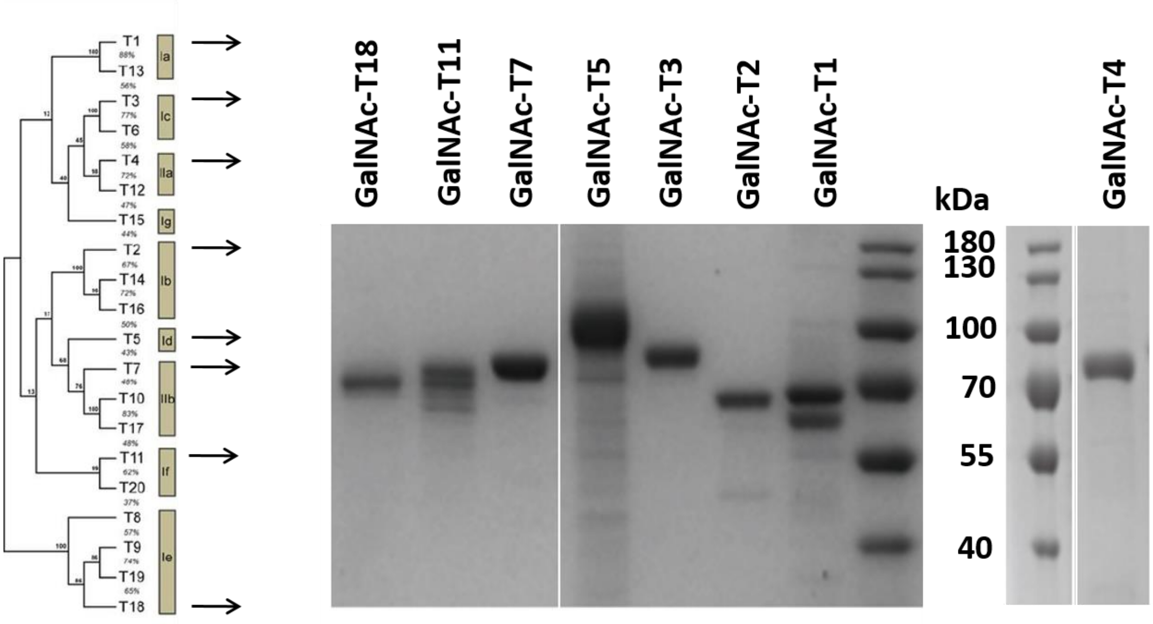

Supplementary Fig. 3 Purified GalNAc-Ts analyzed by SDS-PAGE and Coomassie staining. Soluble GalNAc-T1, T2, T3, T4, T5, T7, T11 and T18 were overexpressed in FreeStyle^{TM} 293-F cell and purified by Ni-NTA chromatography.

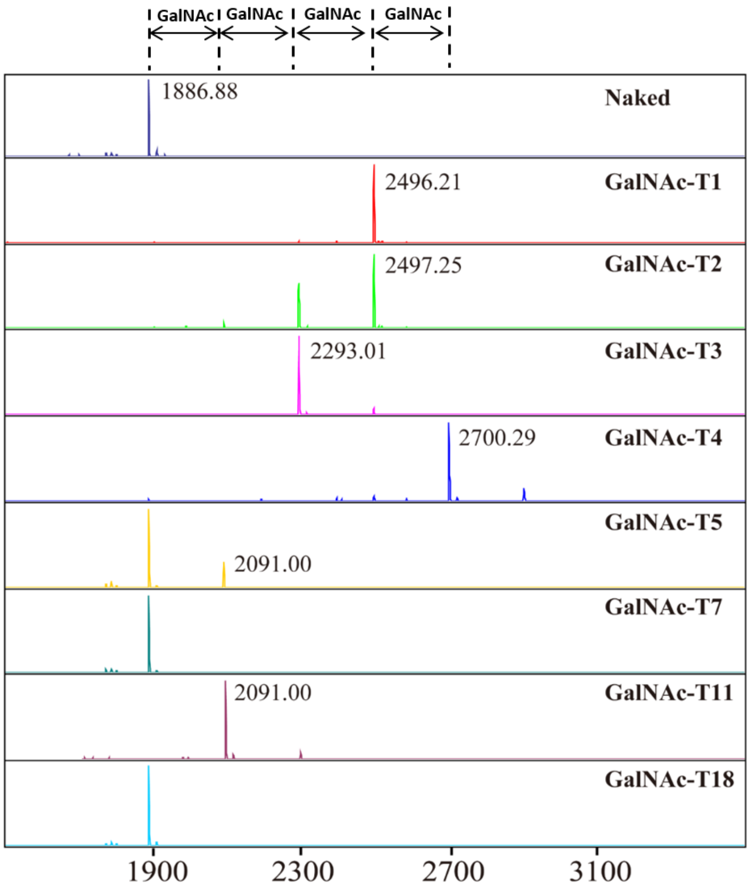

Supplementary Fig. 4 MALDI-TOF analysis of GalNAcylation reactions catalyzed by purified GalNAc-Ts on a synthetic Muc1-derived peptide (HGVTSAPDTRPAP GSTAPPA). Reactions were performed and analyzed as described in the Methods. An increase of 203 Da corresponds to the addition of one GalNAc residue to the peptide.

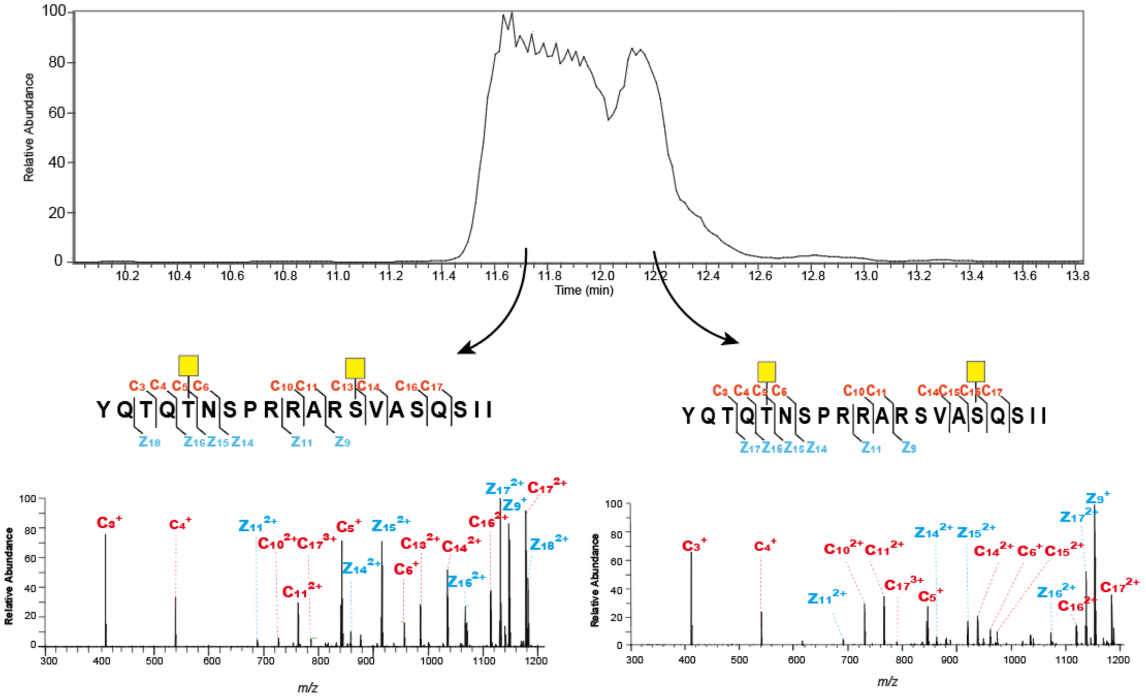

Supplementary Fig. 5 LC-MS analysis of doubly-glycosylated peptide products of GalNAc-T3 and T7 reactions. The extracted ion chromatogram (XIC) shows that the doubly-glycosylated peptide products elute as a broad peak at 11.5-12.5 min, with a pronounced shoulder at ~12.15 min (top panel). The glycosites of the peptides in the main peak and in the shoulder were separately determined from ETD spectra (bottom panel). The mass of *c*- and *z*- fragment ions in the ETD spectra indicated that the main product carries GalNAc modifications at positions corresponding to T678/Ser686 of the spike protein, and the minor product at T678/S689. The glycosylated residues are denoted as yellow squares according to Consortium for Functional Glycomics (CFG) standard.

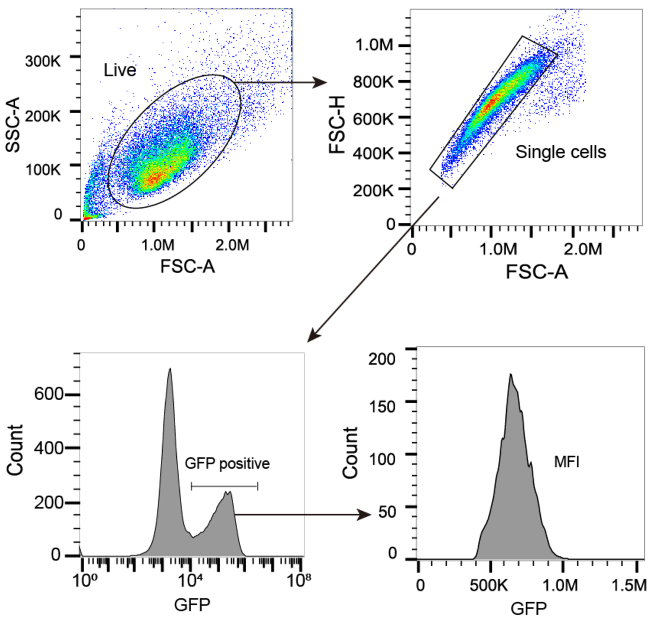

Supplementary Fig. 6 The gating strategy for the flow cytometry analysis of GFP expression in the luciferase-based biosensor assay. All luminescence signals in Fig. 2 of the main text (panel b, c, and d) have been normalized by MFI of GFP expression, following the same gating process illustrated above: Live and single cells were selected based on SSC-A/FSC-A and FSC-H/FSC-A, respectively, and the Mean Fluorescence Intensity (MFI) was obtained by selecting GPF-positive cells.

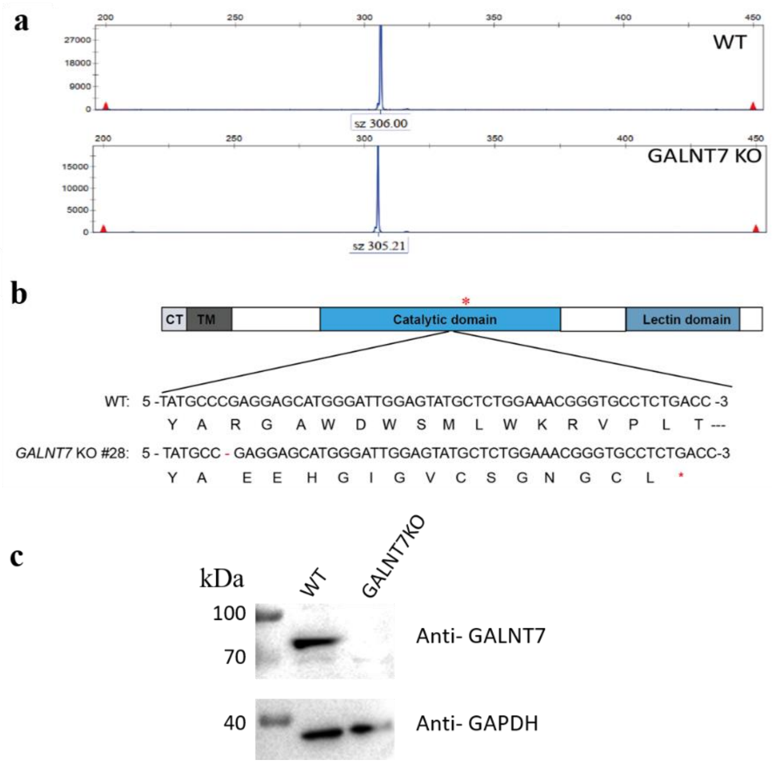

Supplementary Fig. 7 CRISPR-based knockout (KO) of GALNT7 in HEK293T cells. Analysis of the Insertion and Deletion (Indel) of the KO clone by amplicon size analysis (**a**), amplicon sequencing (**b**) and western blot (**c**). The selected KO clone demonstrated deletion of one base pair in the catalytic domain in both alleles, causing a frameshift and early termination as denoted by a red star (*). Source data are provided as a Source Data file.

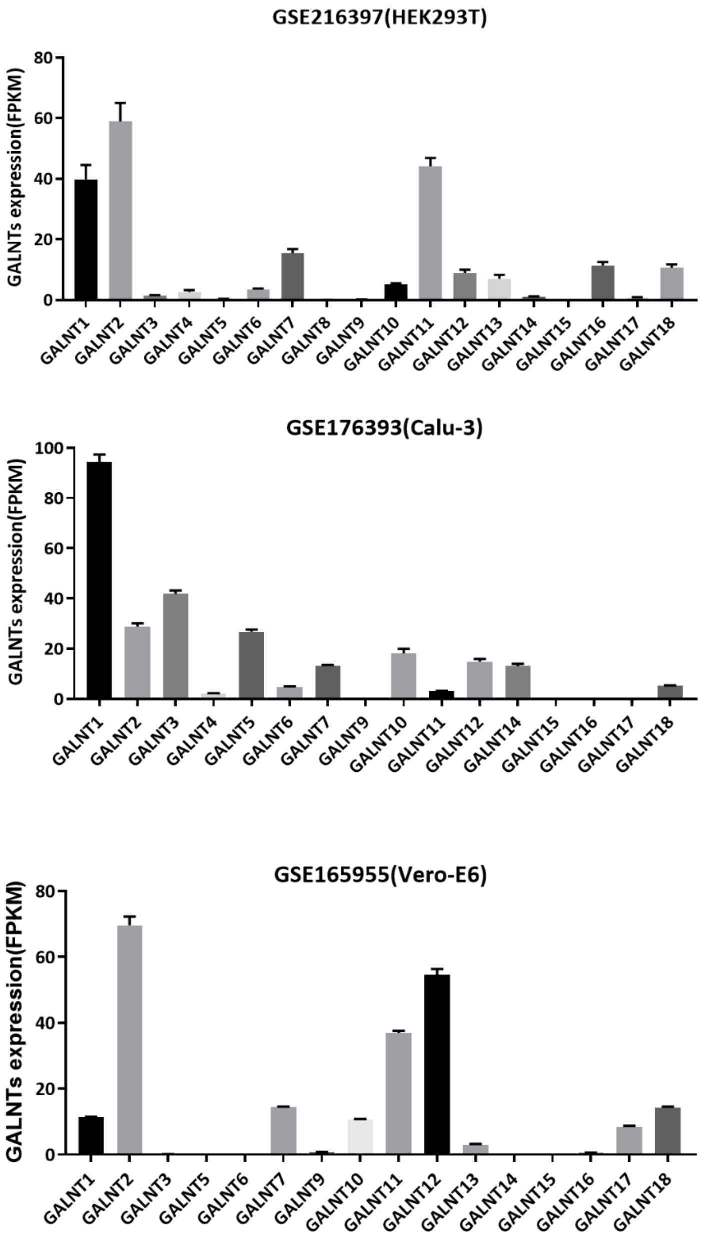

Supplementary Fig. 8 *GALNTs* expression levels in HEK293T, Calu-3 and Vero-E6 cells from RNA-seq analysis. The data for GALNTs expression level was obtained from Gene Expression Omnibus database [https://www.ncbi.nlm.nih.gov/geo/] under the accession GSE216397 [https://www.ncbi.nlm.nih.gov/geo/query/acc.cgi?acc=GSE216397]for HEK293T cell[5], GSE176393 [https://www.ncbi.nlm.nih.gov/geo/query/acc.cgi?acc=GSE176393] for Calu-3 cell[6] and GSE165955

[https://www.ncbi.nlm.nih.gov/geo/query/acc.cgi?acc=GSE165955] forVero-E6[7]. Source data are provided as a Source Data file.

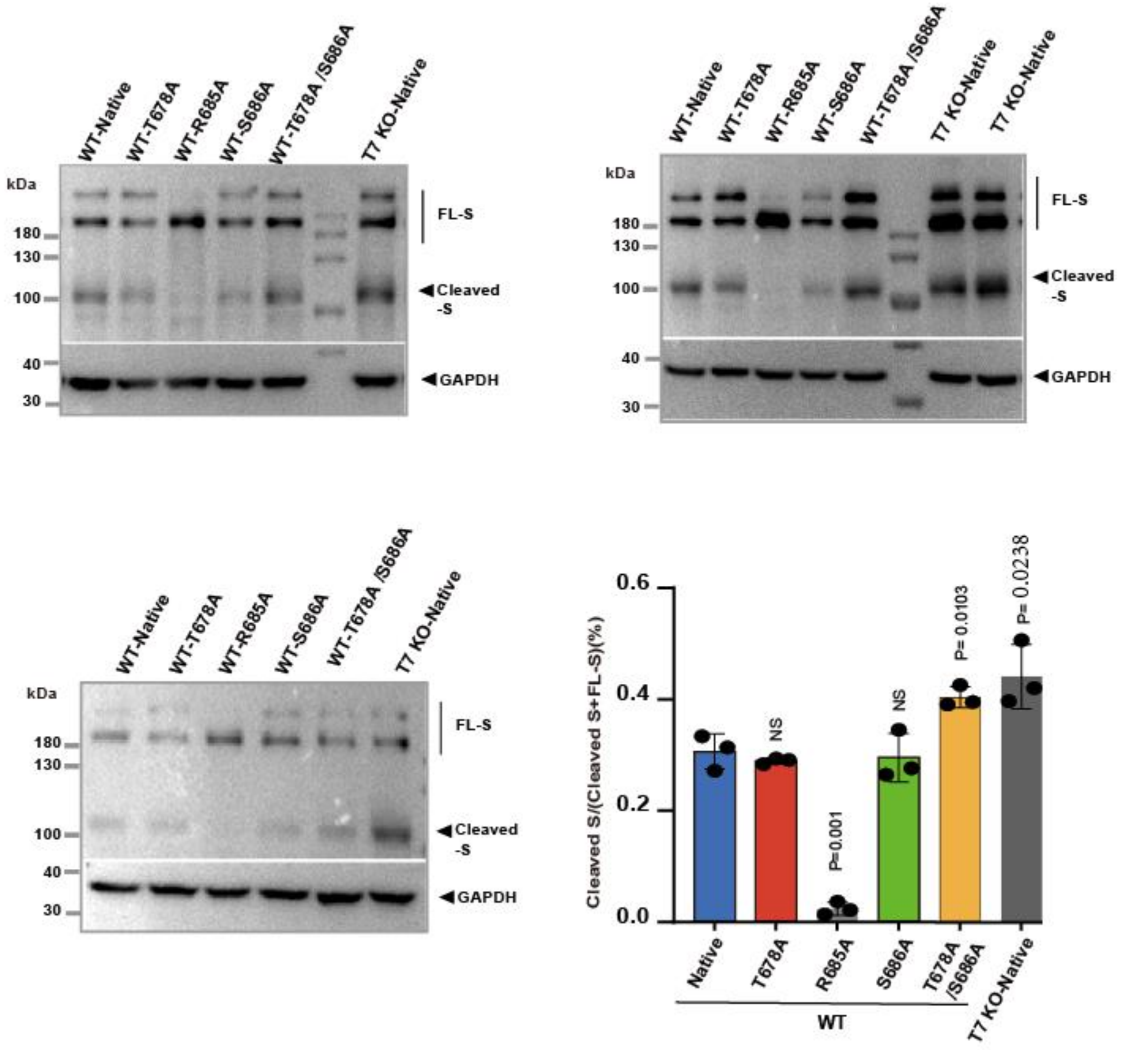

Supplementary Fig. 9 Western blot and quantitative analysis of overexpressed spike protein with glycosite mutations in HEK293T WT and *GALNT7* KO cells. Results here are blots from three independent experiments. Data are presented as mean values ± SD (n = 3 independent experiments) and two-tailed *P*-values are calculated by unpaired Student's t test. Unless otherwise labeled, the displayed *P*-values are the significance between the experimental group and the control group (Native). Source data are provided as a Source Data file.

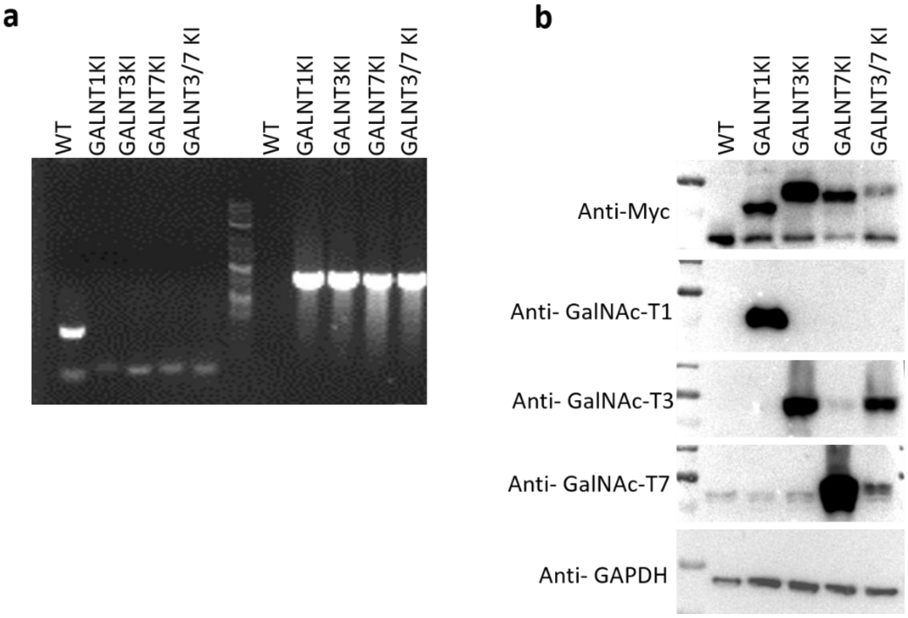

Supplementary Fig . 10 Validation of *GALNT* KIs in HEK293T cells. a, Analysis of targeted integration of *GALNT*s into the AAVS1 loci of HEK293T by WT PCR (left) and junction PCR (right). WT PCR primers are designed to amplify the unmodified AAVS1 site before integration. Junction PCR primers are designed to amplify the junction of the *GALNT* expression cassette with the AAVS1 site to confirm the site-specific integration. The results indicated that the KI clones of *GALNT1*, *GALNT3*, *GALNT7*, and *GALNT3/7* all have the gene(s) correctly integrated into the AAVS1 loci on both alleles. **b,** Western blot analysis showing GalNAc-T1, GalNAc-T3 or GalNAc-T7 expression in HEK293T KI clones. The WT and KI clones of HEK293T cells were detected with anti-myc antibody (myc-tags were fused to GalNAc-Ts in the expression cassette), or antibodies to the GalNAc-Ts. Source data are provided as a Source Data file.

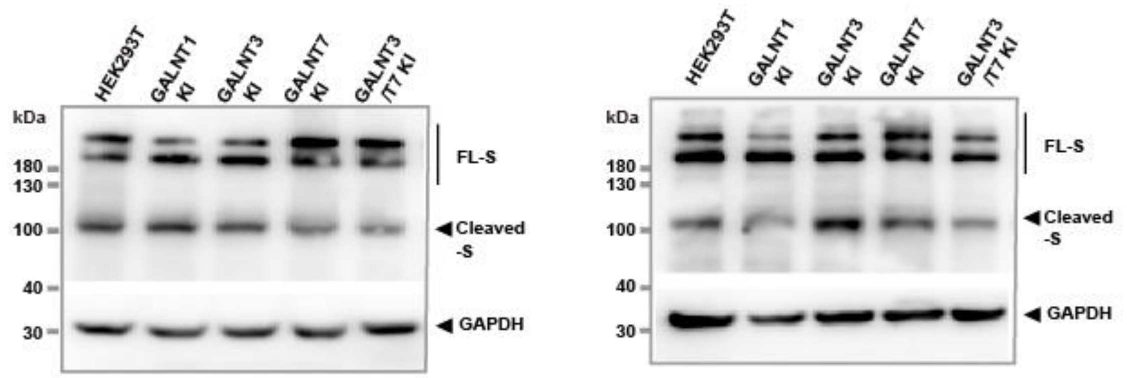

Supplementary Fig. 11 The replicate results of western blot analysis of the processing of spike protein overexpressed in HEK293T WT and *GALNT*s KI cells. The total protein concentrations in cell lysates were measured by BCA assay and normalized for sample loading. GAPDH was used as a loading control. Results here are blots from independent experiments. Source data are provided as a Source Data file.

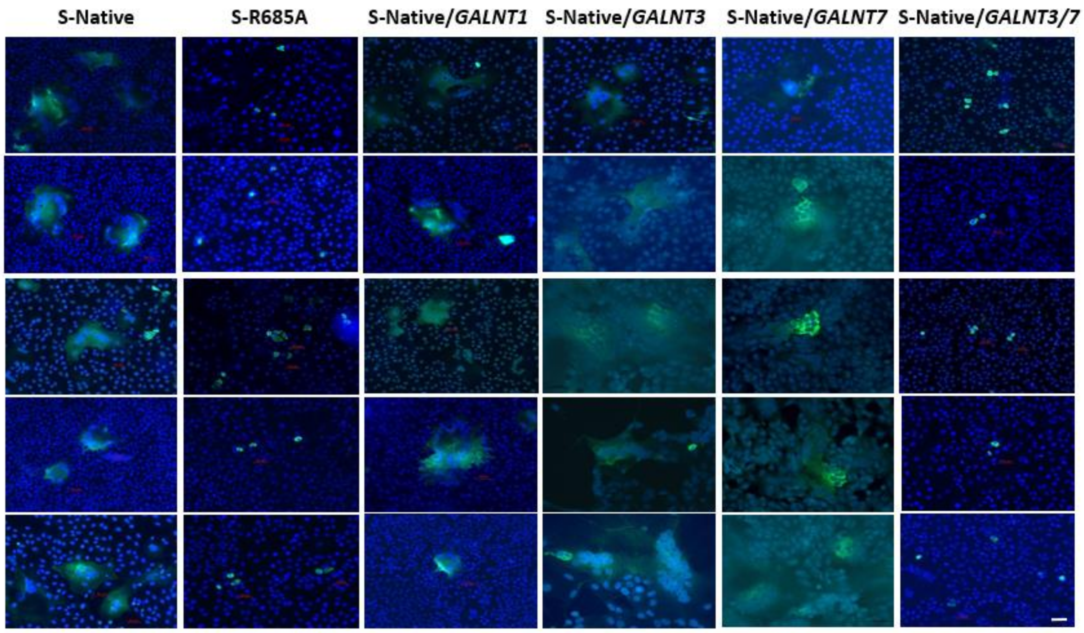

Supplementary Fig. 12 Full field view of syncytia formation assays. Syncytia were visualized as FITC positive cells containing multiple nuclei. Multiple full field-of-view images for each transfection are shown. Scale bar, 50 μm. Source data are provided as a Source Data file.

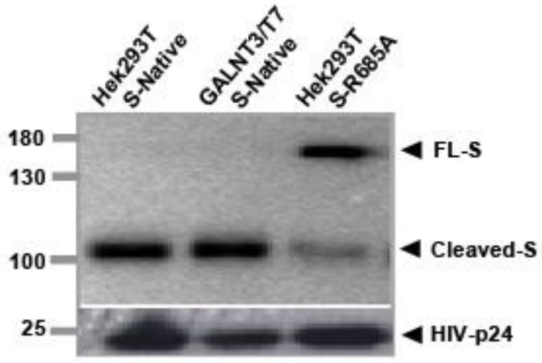

Supplementary Fig. 13 Western blot analysis of S protein packaging into the HIV pseudovirus in HEK293T WT and *GALNT3/T7* KI cells. The results here are representative blots from two independent experiments.

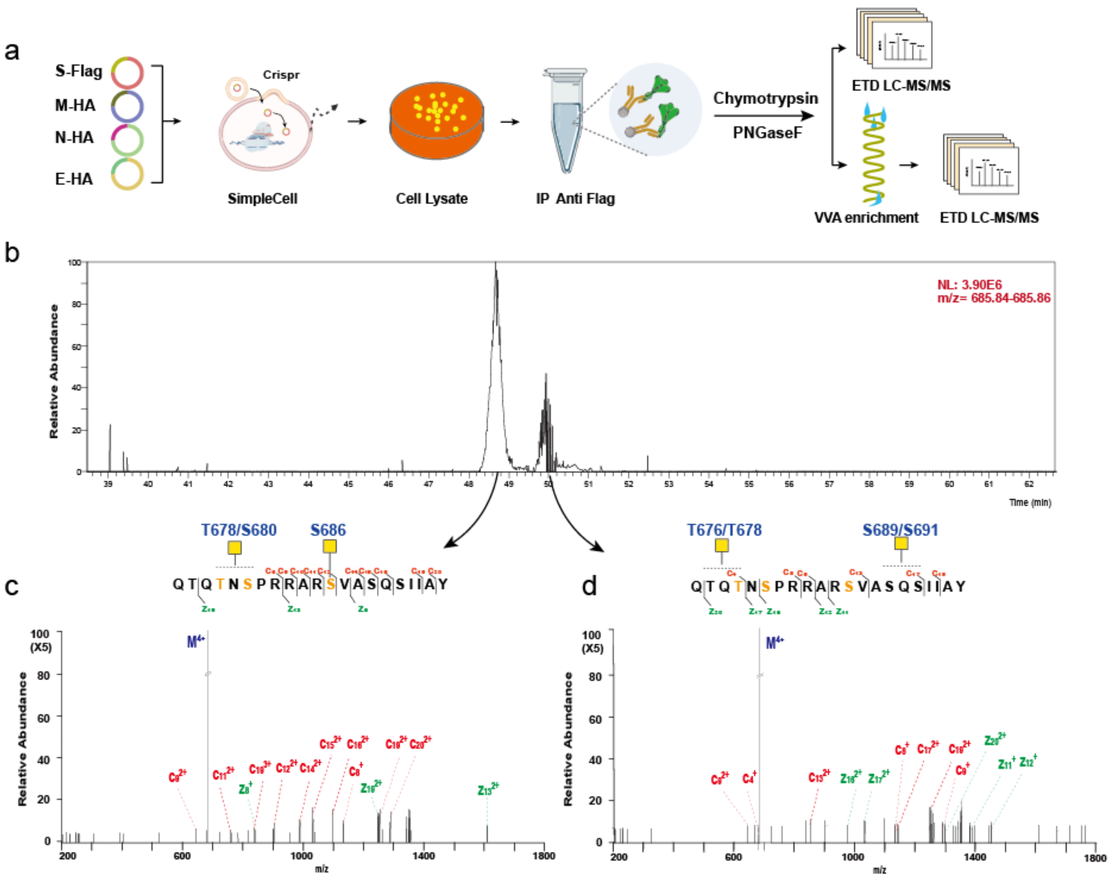

Supplementary Fig. 14 *O*-glycosites identification of full length Spike from cell lysate after VLP packaging. a, Workflow for the bottom-up glyco-analysis of full length Spike from remaining cell lysate of engineered HEK293F after VLP packaging. FreeStyle™ 293-F was engineered by *C1GALT1* gene knockout to produce truncated *O*-glycans and VVA lectin was used for glycopeptide enrichment. MS analysis was performed as described in the method with one sample of the purified full-length Spike (n = 1). The structural models of SARS-CoV-2 Spike protein were generated using Pymol 2.5 (PDB code 7DDD[4] [https://doi.org/10.2210/pdb7DDD/pdb]) **b,** Extracted ions for peptides with two glycosites being GalNAcylated after VVA lectin enrichment. The GalNAc residues are denoted as yellow squares according to Consortium for Functional Glycomics (CFG) standard. **c** and **d,** ETD-MS^2 spectrum of *O*-GalNAcylated peptides

from Spike. The mass of *c*- and *z*- fragment ions indicated that T676/T678, S686, S689/S691 were occupied by *O*-GalNAc.

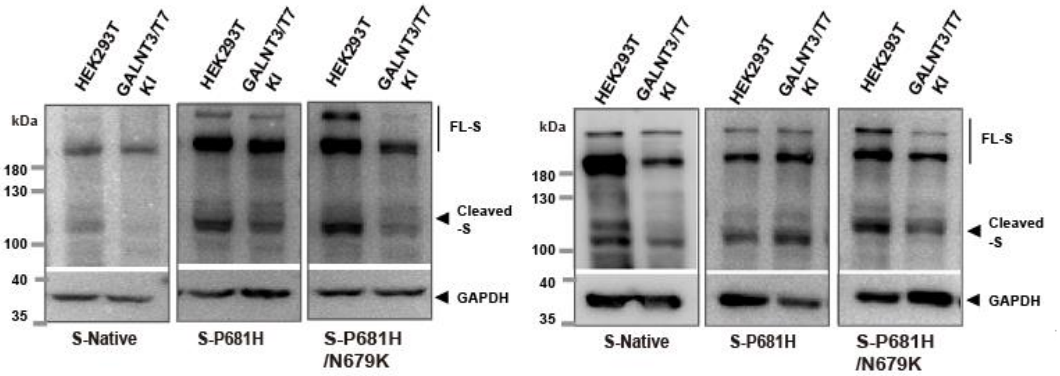

Supplementary Fig. 15 The replicate results of western blot analysis of the P681H and P681H/N679K spike protein processing in HEK293T WT and *GALNT*s KI cells. Results here are blots from independent experiments. Source data are provided as a Source Data file.

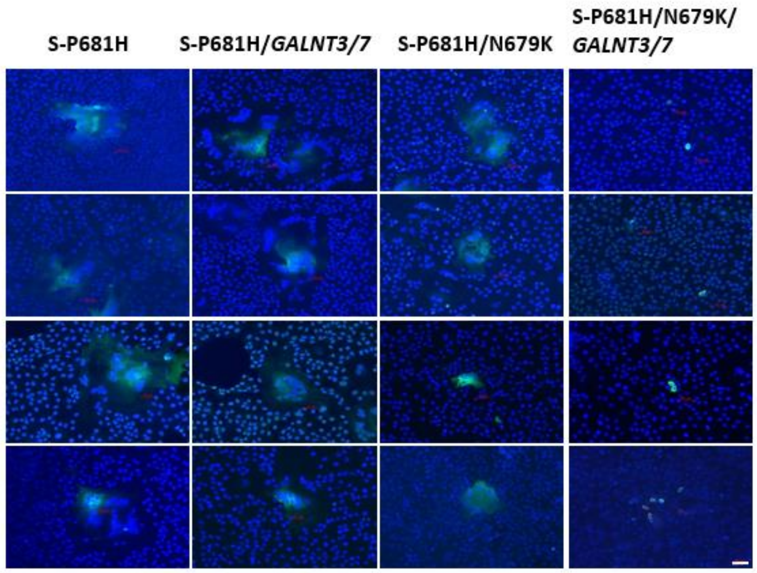

Supplementary Fig. 16 Full field view of syncytia formation assays. Syncytia were visualized as FITC positive cells containing multiple nuclei. Multiple full field-of-view images for each transfection are shown**.** Scale bar, 50 μm. Source data are provided as a Source Data file.

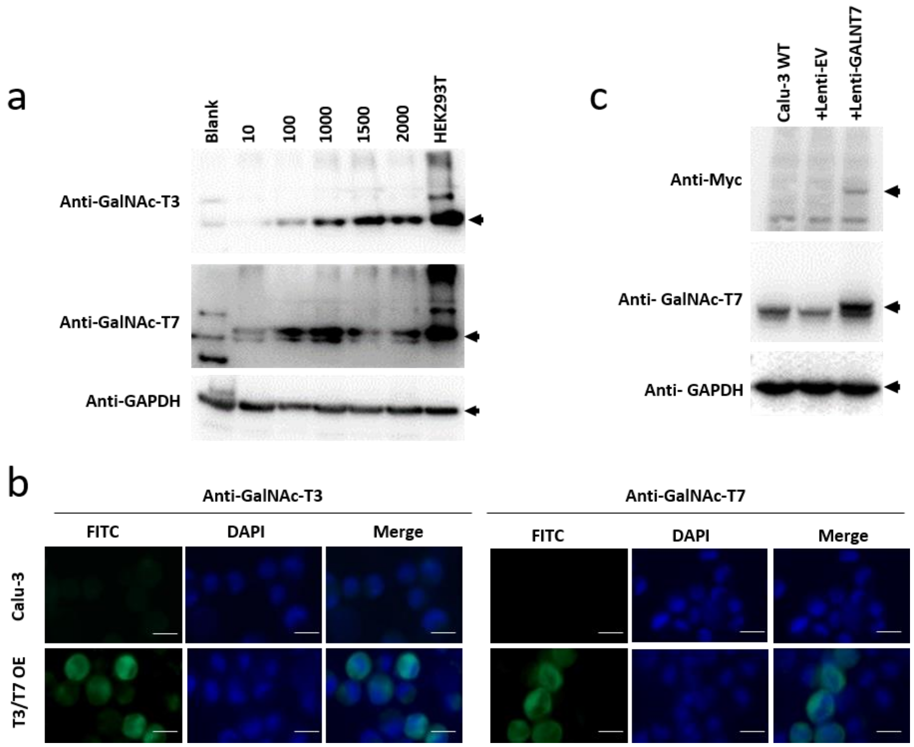

Supplementary Fig. 17 Validation of GALNT3 and/or GALNT7 overexpression in Calu-3 cells. a, Western blot analysis of GalNAc-T3 and GalNAc-T7 overexpression in Ad5-T3/T7 transduced Calu-3 cells with varying MOI. The Ad5-T3/T7 transduced HEK293T cells was used as a positive control. **b**, Immunofluorescence staining of GalNAc-T3 and GalNAc-T7 overexpressed in AdV-T3/T7 transduced Calu-3 cells. Calu-3 cells with or without transduction of Ad5-T3/T7 were stained with DAPI (blue) and immuno-stained with anti-GalNAc-T3 or GalNAc-T7 antibody (green).**c**, Western blot analysis of GalNAc-T7 overexpression in Lenti-GALNT7 transduced Calu-3 cells. Source data are provided as a Source Data file.

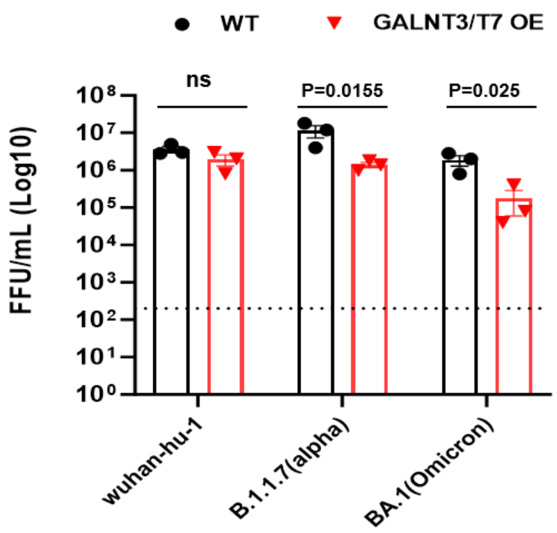

Supplementary Fig. 18 GalNAc-T3 and T7 Inhibit the Replication of SARS-CoV-2 in Calu-3 Cells. Viral titers from Calu-3 cells infected with SARS-CoV-2 wuhan-hu-1, alpha variant or omicron variant at an MOI of 0.01, with or without GalNAc-T3 and T7 overexpression. Data are presented as mean values ± SEM (n = 3 independent experiments) and two-tailed *P*-values are calculated by unpaired Student's t test. Source data are provided as a Source Data file.

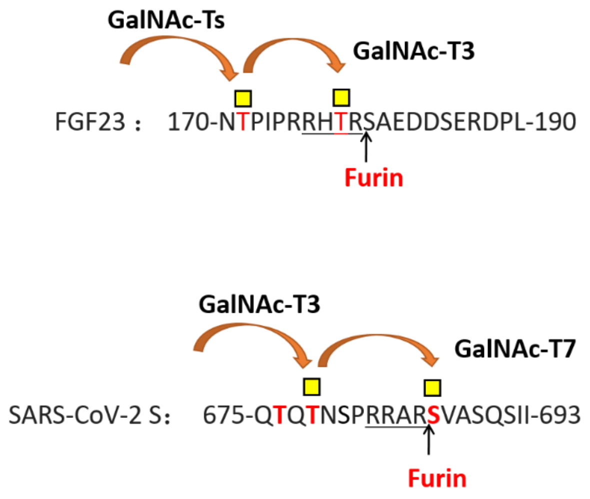

Supplementary Fig. 19 Sequential glycosylation near the furin site of FGF23 and SARS-CoV-2 spike protein. The glycosylation at T178 of FGF23 (within the furin recognition sequence) by GalNAc-T3 requires prior glycosylation at T171 by other GalNAc-Ts. The glycosylation at S686 of SARS-CoV-2 spike protein (right next to the furin site) by GalNAc-T7 requires prior glycosylation at T678 by GalNAc-T3.

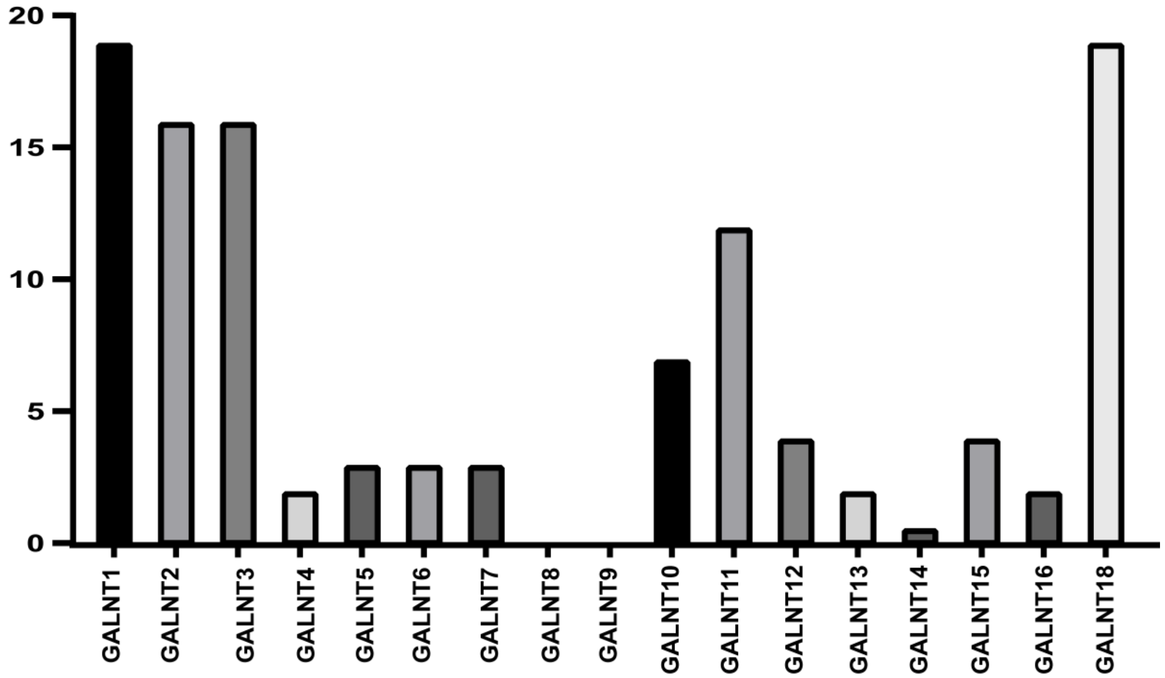

Supplementary Fig. 20 GalNAc-Ts expression in human lung. The data for GALNTs expression level in human lung was obtained from ArrayExpress under the accession no. E-MTAB-513 [http://www.ebi.ac.uk/biostudies/arrayexpress/studies/E-MTAB-513/].

Supplementary References：

1. Steentoft, C., Bennett, E.P. & Clausen, H. Glycoengineering of human cell lines using zinc finger nuclease gene targeting: SimpleCells with homogeneous GalNAc O-glycosylation allow isolation of the O-glycoproteome by one-step lectin affinity chromatography. *Methods Mol Biol* **1022**, 387-402 (2013).
2. Steentoft, C. et al. Precision mapping of the human O-GalNAc glycoproteome through SimpleCell technology. *EMBO J* **32**, 1478-1488 (2013).
3. Steentoft, C. et al. Mining the O-glycoproteome using zinc-finger nuclease-glycoengineered SimpleCell lines. *Nat Methods* **8**, 977-982 (2011).
4. Zhang, C. et al. Development and structural basis of a two-MAb cocktail for treating SARS-CoV-2 infections. *Nat Commun* **12**, 264 (2021).
5. Yang, Y. et al. An intellectual disability-related MED23 mutation dysregulates gene expression by altering chromatin conformation and enhancer activities. *Nucleic Acids Res* **51**, 2137-2150 (2023).
6. Zhuang, X. et al. The circadian clock component BMAL1 regulates SARS-CoV-2 entry and replication in lung epithelial cells. *iScience* **24**, 103144 (2021).
7. Cheng, K. et al. Genome-scale metabolic modeling reveals SARS-CoV-2-induced metabolic changes and antiviral targets. *Mol Syst Biol* **17**, e10260 (2021).
