# Supplementary material for: Sequential glycosylations at the multibasic cleavage site of SARS-CoV-2 spike protein regulate viral activity
Source: Nat Commun. 2024 May 16;15:4162. doi: 10.1038/s41467-024-48503-x (PMC11099032; doi:10.1038/s41467-024-48503-x)
Supplement: Supplementary file 3 — Description of Additional Supplementary Files [file 41467_2024_48503_MOESM3_ESM.pdf]

Description of Additional Supplementary Files:

Supplementary Data 1: Summary of identified O-glycosylation sites on SARS-CoV-2 spike.

Supplementary Data 2: Primers used in this study.
