# Supplementary material for: Sequential glycosylations at the multibasic cleavage site of SARS-CoV-2 spike protein regulate viral activity
Source: Nat Commun. 2024 May 16;15:4162. doi: 10.1038/s41467-024-48503-x (PMC11099032; doi:10.1038/s41467-024-48503-x)
Supplement: Supplementary file 6 — Reporting Summary [file 41467_2024_48503_MOESM6_ESM.pdf]

nature portfolio

Corresponding author(s): Yanqiu Yuan, Jincun Zhao, Yang Mao

Last updated by author(s): Apr 24, 2024

# Reporting Summary

Nature Portfolio wishes to improve the reproducibility of the work that we publish. This form provides structure for consistency and transparency in reporting. For further information on Nature Portfolio policies, see our Editorial Policies and the Editorial Policy Checklist.

## Statistics

For all statistical analyses, confirm that the following items are present in the figure legend, table legend, main text, or Methods section.

| n/a | Confirmed | |
|---|---|---|
| ☐ | ☒ | The exact sample size ($n$) for each experimental group/condition, given as a discrete number and unit of measurement |
| ☐ | ☒ | A statement on whether measurements were taken from distinct samples or whether the same sample was measured repeatedly |
| ☐ | ☒ | The statistical test(s) used AND whether they are one- or two-sided<br>*Only common tests should be described solely by name; describe more complex techniques in the Methods section.* |
| ☒ | ☐ | A description of all covariates tested |
| ☒ | ☐ | A description of any assumptions or corrections, such as tests of normality and adjustment for multiple comparisons |
| ☐ | ☒ | A full description of the statistical parameters including central tendency (e.g. means) or other basic estimates (e.g. regression coefficient) AND variation (e.g. standard deviation) or associated estimates of uncertainty (e.g. confidence intervals) |
| ☐ | ☒ | For null hypothesis testing, the test statistic (e.g. $F$, $t$, $r$) with confidence intervals, effect sizes, degrees of freedom and $P$ value noted<br>*Give P values as exact values whenever suitable.* |
| ☒ | ☐ | For Bayesian analysis, information on the choice of priors and Markov chain Monte Carlo settings |
| ☒ | ☐ | For hierarchical and complex designs, identification of the appropriate level for tests and full reporting of outcomes |
| ☐ | ☒ | Estimates of effect sizes (e.g. Cohen's $d$, Pearson's $r$), indicating how they were calculated |

*Our web collection on statistics for biologists contains articles on many of the points above.*

## Software and code

Policy information about availability of computer code

| | |
|---|---|
| Data collection | Zeissi Axio Scope. A1 Fluorscence microscope (Germany) was used to collect the fluorescence images; The fluorescence images were processed using ZEN Blue 2.3; Western blot figures were acquired by AllCap Software on TANON Imager 5200 multi; Bioluminescence signals were measured on a GloMax® Microplate Luminometer(Promega) with GloMax Navigator software; Flow cytometry data were acquired by CytoFLEX Software on flow cytometer (Beckman CytoFLEX); Mass Spectrometry datasets were collected using Orbitrap Fusion Tribrid Mass Spectrometer coupled with an Easy-nLC 1200 system (Thermo Fisher Scientific); The MS was operated using Xcalibur software (version 4.1); The foci were visualized by TrueBlue Peroxidase Substrate (KPL, Gaithersburg, MD); The foci were counted with an ELISPOT reader (Cellular Technology Ltd. Cleveland, OH); Maldi-TOF data were collected with ULTRAFLEXTREME mass spectrometer (Bruker, Leipzig, Germany). |
| Data analysis | GraphPad Prism 7 was used for quantitative data analysis ;Image J was used for densitometry analysis of western blot results; Broker Daltonis flexAnalysis was used to analyze MALDI-TOF spectra; FlowJo 10 was used analyze flow cytometry results; Proteome Discoverer 2.5 software was used for MS/MS database search; Pymol 2.5 was used to generate structural models for figure 6, Supplementary Figure 1a and Supplementary Figure 13a; Adobe Illustrator 2020 was used to create drawings in Figure 2a, Supplementary Figure 1a and Supplementary Figure 13a |

For manuscripts utilizing custom algorithms or software that are central to the research but not yet described in published literature, software must be made available to editors and reviewers. We strongly encourage code deposition in a community repository (e.g. GitHub). See the Nature Portfolio guidelines for submitting code & software for further information.



April 2023

## Data

Policy information about availability of data

All manuscripts must include a data availability statement. This statement should provide the following information, where applicable:

- Accession codes, unique identifiers, or web links for publicly available datasets
- A description of any restrictions on data availability
- For clinical datasets or third party data, please ensure that the statement adheres to our policy

The authors declare that all data supporting the findings of this study are available within the paper and its Supplementary Information. Source data are provided with this paper. The mass spectrometry proteomics data have been deposited to the ProteomeXchange Consortium via the PRIDE77 partner repository with the dataset identifier PXD049110 [http://proteomecentral.proteomexchange.org/cgi/GetDataset?ID=PXD049110]. The protein sequence of SARS-COV-2 Spike in UniProt database with ID P0DTC2 [https://covid19.uniprot.org/uniprotkb/P0DTC2] was used as a reference for mass spectrometry database search. The structural models of the Spike protein and Membrane protein were downloaded from Protein Data Bank with PDB ID 7DDD [https://doi.org/10.2210/pdb7DDD/pdb] and 7VGR [https://doi.org/10.2210/pdb7vgr/pdb]. The data for GALNTs expression level was obtained from Gene Expression Omnibus database with accession GSE216397 [https://www.ncbi.nlm.nih.gov/geo/query/acc.cgi?acc=GSE216397] for HEK293T cell, GSE176393 [https://www.ncbi.nlm.nih.gov/geo/query/acc.cgi?acc=GSE176393] for Calu-3 cell and GSE165955 [https://www.ncbi.nlm.nih.gov/geo/query/acc.cgi?acc=GSE165955] for Vero-E6. The data for GALNTs expression level in human lung was obtained from ArrayExpress with accession no. E-MTAB-513 [http://www.ebi.ac.uk/biostudies/arrayexpress/studies/E-MTAB-513/]. The sequences of Alpha and Omicron Spike can be found with accession no. QUV36347.1 [https://www.ncbi.nlm.nih.gov/protein/QUV36347] and UPX99225.1 [https://www.ncbi.nlm.nih.gov/protein/ UPX99225.1]. The sequences of S, M, N, E, GalNAc-T1, GalNAc-T3 and GalNAc-T7 proteins can be found with RefSeq no. YP_009724390.1 [https://www.ncbi.nlm.nih.gov/protein/YP_009724390.1] for S, RefSeq no. YP_009724393.1 [https://www.ncbi.nlm.nih.gov/protein/YP_009724393.1] for M, YP_ 009724397.2 [https://www.ncbi.nlm.nih.gov/protein/ YP_ 009724397.2] for N, YP_009724392.1 [https://www.ncbi.nlm.nih.gov/protein/ YP_009724392.1] for E, NP_001371368.1 [https://www.ncbi.nlm.nih.gov/protein/ NP_001371368.1] for GalNAc-T1, NP_004473.2 [https://www.ncbi.nlm.nih.gov/protein/NP_004473.2] for GalNAc-T3 and NP_001362529.1 [https://www.ncbi.nlm.nih.gov/protein/NP_001362529.1] for GalNAc-T7. The sequencing data for Wuhan-hu-1 strain of SARS-CoV-2 can be found in GenBank with accession no. MT123290 [https://www.ncbi.nlm.nih.gov/nuccore/MT123290]. The sequencing data for alpha strain (B.1.1.7) and omicron strain (BA.1) used in this study have been deposited to GenBase (https://ngdc.cncb.ac.cn/genbase/) with restricted access (accession code C_AA068118.1 and C_AA068119.1, respectively), and access to those sequences can be requested from the corresponding authors.

## Research involving human participants, their data, or biological material

Policy information about studies with human participants or human data. See also policy information about sex, gender (identity/presentation), and sexual orientation and race, ethnicity and racism.

| | |
|---|---|
| Reporting on sex and gender | No human data |
| Reporting on race, ethnicity, or other socially relevant groupings | No human data |
| Population characteristics | No human data |
| Recruitment | No human data |
| Ethics oversight | No human data |

Note that full information on the approval of the study protocol must also be provided in the manuscript.

# Field-specific reporting

Please select the one below that is the best fit for your research. If you are not sure, read the appropriate sections before making your selection.

☒ Life sciences ☐ Behavioural & social sciences ☐ Ecological, evolutionary & environmental sciences

For a reference copy of the document with all sections, see nature.com/documents/nr-reporting-summary-flat.pdf

# Life sciences study design

All studies must disclose on these points even when the disclosure is negative.

| | |
|---|---|
| Sample size | The exact sample size are specified in the figure legends. No sample-size calculations were performed to power each study. |
| Data exclusions | No data were excluded from the analyses |
| Replication | All experiments had at least 2 independent biological replicates and indicated in the figure legends. All replication attempts were successful. |
| Randomization | Not relevant to the study because there is no grouping in our experiments. All the cells were imaged or analyzed with the same way. |
| Blinding | Not relevant to the study because there is no grouping in our experiments. The key experiments were repeated independently by multiple |

| | |
|---|---|
| Blinding | members of the laboratory. All the results are quantitative and did not require subjective judgment or interpretation, so the blinding was not necessary. |

# Reporting for specific materials, systems and methods

We require information from authors about some types of materials, experimental systems and methods used in many studies. Here, indicate whether each material, system or method listed is relevant to your study. If you are not sure if a list item applies to your research, read the appropriate section before selecting a response.

## Materials & experimental systems

| n/a | Involved in the study | |
|---|---|---|
| ☐ | ☒ | Antibodies |
| ☐ | ☒ | Eukaryotic cell lines |
| ☒ | ☐ | Palaeontology and archaeology |
| ☒ | ☐ | Animals and other organisms |
| ☒ | ☐ | Clinical data |
| ☒ | ☐ | Dual use research of concern |
| ☒ | ☐ | Plants |

## Methods

| n/a | Involved in the study | |
|---|---|---|
| ☒ | ☐ | ChIP-seq |
| ☒ | ☐ | Flow cytometry |
| ☒ | ☐ | MRI-based neuroimaging |

## Antibodies

Antibodies used

1) Rabbit polyclonal anti-GAPDH protein Proteintech Cat# 10494-1-AP; RRID:AB_2263076 Lot: 00109152 (1:2500)
2) Rabbit polyclonal anti-GALNT1 Sigma-Aldrich Cat# HPA012628; RRID:AB_1849439 Lot:A115764 (1:1000)
3) Rabbit polyclonal anti-GALNT3 Sigma-Aldrich Cat# HPA007613; RRID:AB_1078941 Lot:A114649 (1:1000)
4) Rabbit polyclonal anti-GALNT7 Sigma-Aldrich Cat# HPA064243; RRID:AB_2685223 Lot: 000010086 (1:1000)
5) HA tag polyclonal antibody proteintech Cat# 51064-2-AP; RRID:AB_11042321 Lot: 00106228 (1:100)
6) Mouse polyclonal anti-Flag protein Sigma-Aldrich Cat# F1804; RRID:AB_262044 Lot: SLCD6338 (1:1000)
7) Mouse polyclonal anti-Myc protein Abcam Cat# Ab32; RRID:AB_303599 Lot:GR3272830-2 (1:2000)
8) Mouse/human monoclonal anti-SARS-CoV-2 S protein (S2-specific) Sino Biological Cat# 40590-D001; RRID: AB_2857932 Lot: HA14AP2901-B (1:1000)
9) Rabbit monoclonal anti-SARS-CoV-2 S1 protein, GeneTex Cat# GTX635654; RRID: AB_2888548; Lot:44515 (1:1000)
10) Mouse monoclonal anti- SARS-CoV-2 Nucleocapsid protein, GenScript Cat# A02050 Lot:20H002225(1:2000)
11) 40590-D001
12) Goat anti-mouse, FITC-linked antibody Invitrogen Cat# A16079, RRID:AB_2534753 Lot:38-119-022014(1:1000)
13) Rabbit anti-Human, HRP-linked antibody Abcam Cat# Ab6759, RRID:AB_955434 Lot: GR3347373-3(1:2000)
14) Goat anti-mouse, HRP-linked antibody Invitrogen Cat# 31430 RRID: AB_228307 Lot:XH363702(1:3000)
15) Goat anti- rabbit, HRP-linked antibody Cell Signaling Technology Cat# 7074,RRID:AB_2099233 Lot: 30(1:3000)
16) HRP Conjugated Mouse monoclonal anti-HA protein (6E2) Cell Signaling Technology Cat# 2999S, Lot: 5 (1:1000)
17) Anti-FLAG Magnetic Agarose Invitrogen Cat# A36797, Lot: WH328207

Validation

All the commercial primray antibodies were validated by the manufacturers and validation statements are available on the manufacturers's website. Some antibodies were also validated by relevant citations and/or the data provided in the manuscript.
1) Rabbit polyclonal anti-GAPDH protein(Proteintech, 10494-1-AP), Commercially validated by WB, IP,IHC, FC and ELISA. https://www.ptglab.com/Products/Pictures/pdf/10494-1-AP.pdf
2) Rabbit polyclonal anti-GALNT1 (Sigma HPA012628), Commercially validated by WB and IHC.(https://www.sigmaaldrich.cn/CN/en/product/sigma/hpa012628).
3) Rabbit polyclonal anti-GALNT3 (Sigma, HPA007613), Commercially validated by WB and IHC (https://www.sigmaaldrich.cn/CN/en/product/sigma/hpa007613).
4) Rabbit polyclonal anti-GALNT7 (Sigma, HPA064243), Commercially validated by WB and IHC. (https://www.sigmaaldrich.cn/CN/en/product/sigma/hpa064243).
5) Mouse polyclonal anti-HA protein (6E2) (Cell Signaling Technology, 2367S), Commercially validated by WB, IP,IHC and ChIP, et al. (https://www.cellsignal.cn/products/primary-antibodies/ha-tag-6e2-mouse-mab/2367.cellsignal.cn).
6) Mouse polyclonal anti-Flag protein (Sigma, F1804),Commercially validated by WB, IP,IHC, ChIP and EIA, et al. https://www.sigmaaldrich.cn/CN/en/product/sigma/f1804
7) Mouse polyclonal anti-Myc protein (Abcam, Ab32), Commercially validated by ICC/IF, Flow Cyt, WB, IP, ELISA, IHC-Fr and Purification.(https://www.abcam.com/products/primary-antibodies/myc-tag-antibody-9e10-ab32.html)
8) Mouse/human monoclonal anti-SARS-CoV-2 S protein (S2-specific)(Sino Biological, 40590-D001), Commercially validated by ELISA, FCM, ICC/IF and Neutralization.(https://cdn.sinobiological.com/antibodies/cov-spike-40150-d001); Validated by WB in Reference: Cell, 2020, 183(3):739-751.e8.
9)Rabbit monoclonal anti-SARS-CoV-2 S1 protein (GeneTex, GTX635654). Commercially validated by WB, ICC/IF, IHC-P,FACS,ELISA,Sandwich ELISA and IHC-P (cell pellet).(https://www.genetex.cn/PDF/Download?catno=GTX635654)
10) Mouse monoclonal anti- SARS-CoV-2 Nucleocapsid protein (GenScript, A02050). Commercially validated by WB and ELISA. (https://www.genscript.com/antibody/A02050-SARS_CoV_2_Nucleocapsid_Antibody_23F2_mAb_Mouse.html)
11) Mouse monoclonal anti- HIV1-P24 (sigma, ab9071), Commercially validated by WB, Radioimmunoprecipitation, ELISA, ICC/IF, and Sandwich ELISA.(https://www.abcam.com/products/primary-antibodies/hiv1-p24-antibody-3954a-ab9071.html)
12) Goat anti-mouse, FITC-linked antibody (Invitrogen, A16079), Commercially validated by Immunocytochemistry and Flow Cytometry. (https://www.thermofisher.com/antibody/product/Goat-anti-Mouse-IgG-H-L-Highly-Cross-Adsorbed-Secondary-Antibody-Polyclonal/A16079)
13) Rabbit anti-Human, HRP-linked antibody (Abcam, Ab6759), Commercially validated by Immunomicroscopy, WB, ICC and ELISA.

(https://www.abcam.com/products/secondary-antibodies/rabbit-human-igg-hl-hrp-ab6759.html)
14) Goat anti-mouse, HRP-linked antibody (Invitrogen, 31430), Commercially validated by WB, ELISA and IP. (https://www.thermofisher.com/antibody/product/Goat-anti-Mouse-IgG-H-L-Secondary-Antibody-Polyclonal/31430)
15) Goat anti- rabbit, HRP-linked antibody, (Cell Signaling Technology, 7074), Commercially validated by WB, IP and IHC. (https://www.cellsignal.com/products/secondary-antibodies/anti-rabbit-igg-hrp-linked-antibody/7074)
16) HRP Conjugated Mouse monoclonal anti-HA protein (6E2) (Cell Signaling Technology,2999S), Commercially validated by WB, IPJHC and ChIP. (https://www.cellsignal.com/products/antibody-conjugates/ha-tag-6e2-mouse-mab-hrp-conjugate/2999)
17) Anti-FLAG Magnetic Agarose (Invitrogen, A36797), Commercially validated by protein purification and immunoprecipitation. (https://www.thermofisher.com/order/catalog/product/A36797).

## Eukaryotic cell lines

Policy information about cell lines and Sex and Gender in Research

| | |
|---|---|
| Cell line source(s) | HEK293T (Cat# CC4003) and Calu-3 (Cat# CC0213) cells were purchased from Cellcook Biotech Co., Ltd. Vero E6 cell was obtained from ATCC (Cat# CRL1586). |
| Authentication | Commercially validated cell lines were obtained from ATCC and other sources and grew and performed as expected. Morphology of each cell line was assessed by microscopy. All three cell lines were authenticated by STR profiling. |
| Mycoplasma contamination | All cell lines are routinely tested each month and were negative for mycoplasm. |
| Commonly misidentified lines (See ICLAC register) | No commonly misidentified cell lines were used in this study. |
