# Supplementary material for: Sequential glycosylations at the multibasic cleavage site of SARS-CoV-2 spike protein regulate viral activity
Source: Nat Commun. 2024 May 16;15:4162. doi: 10.1038/s41467-024-48503-x (PMC11099032; doi:10.1038/s41467-024-48503-x)
Supplement: Supplementary file 2 — Peer Review File [file 41467_2024_48503_MOESM2_ESM.pdf]

nature portfolio

Peer Review File

Sequential Glycosylations at the Multibasic Cleavage Site of SARS-CoV-2 Spike Protein Regulate Viral Activity

Open Access This file is licensed under a Creative Commons Attribution 4.0 International License, which permits use, sharing, adaptation, distribution and reproduction in any medium or format, as long as you give appropriate credit to the original author(s) and the source, provide a link to the Creative Commons license, and indicate if changes were made. In the cases where the authors are anonymous, such as is the case for the reports of anonymous peer reviewers, author attribution should be to 'Anonymous Referee' followed by a clear attribution to the source work. The images or other third party material in this file are included in the article's Creative Commons license, unless indicated otherwise in a credit line to the material. If material is not included in the article's Creative Commons license and your intended use is not permitted by statutory regulation or exceeds the permitted use, you will need to obtain permission directly from the copyright holder. To view a copy of this license, visit http://creativecommons.org/licenses/by/4.0/.

Reviewer #1 (Remarks to the Author):

The paper by Wang et al describes impact of O-glycosylation on convertase-mediated cleavage of the SARS-CoV2-Spike protein and impact of the cleavage on the assembly of viral particles. The impact of O-glycosylation on the cleavage of the spike was previously described, as the authors acknowledge, but the impact on VLP assembly is novel and interesting. The need for a sequential activity of GalNAc T3/T7 is also novel. However, the presented analyses and data interpretation strategies have a number of limitations that make the results less convincing. The major weaknesses are summarized below, followed by additional points that need to be addressed.

Major concerns:

1. Most of the studies were done in HEK293 cells, a marginally relevant model.

2. A major shortcoming is the lack of any discussion of the endogenous expression of the GalNAcT enzymes. Which of the enzymes are expressed in the HEK or Vero cell lines and how does that affect the outcomes? The endogenous expression affects also many of the presented experimental results which needs to be clarified. For example, the biosensor experiments should be accompanied by KO of the T3 and T7 enzymes and subsequent compensation of the KO by inducible expression of the genes, as published in the original papers. The biosensor data (Figure 2b) or the spike cleavage data (Figure 2d and S Figure5) show that the endogenous activity is significant.

3. The Western blot analyses are confusing. First, spike protein is secreted. Why do you analyze the spike proteins (full length and cleaved) in cell lysates? Second, the bands presented in the paper are not interpretable. Please show in the main text tables of data with clear statistical evaluation or the graphs from supplemental figures with full description of the analyses.

4. The paper does not describe what statistical tests were used to compare the outcomes, besides t-test, which is inadequate. We want to know, for example, whether T3/T7 KI differs from T7 KI, in addition to whether it differs from the WT control. Please update the analyses and their descriptions; it is not clear what exactly was quantified (which bands, images) and how.

5. The connection between O-glycosylation (T3/T7 model) and convertase cleavage of the spike is, overall, weak. Double mutation of the glycosylated amino acids changes cleavage of the spike from 20 to 30%; knock in of both T3 and T7 changes the cleavage from about 25% to 18%. At the same time, the baseline cleavage in WT cells changes from 20% to 30% without any apparent reason (SF5 and 8). The observed changes (due to KO or KI) are the maximal impacts expected and are rather small. A major impact of O-glycosylation in vivo needs therefore more evidence. Please discuss the quantitative aspects in full detail to support your model that connects convertase cleavage, viral assembly, and glycosylation. All the pieces likely happen, to some extent, but their interdependence and their impact on the disease outcomes may need additional evidence.

6. The proposed model of the M-S interaction needs further validation by direct measurements of the binding.

Specific suggestions:

1. Abstract line 2: what is “increased infectivity”? It is essential for infectivity or it increases infectivity.

2. Page 5 describes reports on O-glycosylation of the spike (ref 22-33). The paper will be stronger if you include a table (perhaps in the supplement) which describes clearly what sites were described as O-glycosylated and which of the reports do you find conclusive; the references are of mixed quality. Do you find the analyses in HEK293 cells sufficient? Do the studies agree in their conclusions? Does the evidence conclusively document O-glycosylation under relevant in vivo conditions? Is the extent of the glycosylation suggestive of a major regulatory impact? This deserves a better discussion.

3. The occupancy of the O-glycosites is particularly puzzling; a major inhibitory effect on convertase processing does not seem plausible based on a modification observed on a small percentage of the spike molecules. Please discuss and provide more evidence (on page 5 and throughout the text).

4. Page 6 cites references describing that sialylation of the O-glycans is relevant. Your study did not look at the structures of the O-glycans at all. You simply report O-glycan yes/no using methods which can hardly quantify (MALDI or ETD MS). The type of glycans and the extent of glycosylation (occupancy) in the spike protein remain undefined which limits the value of the study. This needs to be at least discussed extensively as a limitation of the study.

5. Page 7 line 3: which harbors …

6. Page 7 last line: “we found that GalNAc-T3 demonstrated the most substantial activity”. What does that mean? Please define the numbers and show how much more active the T3 enzyme is than others. Is your readout quantitative? Are the enzymes compared under relevant conditions? Perhaps each of them requires a different set of conditions for optimal activity. Please discuss and document that your analysis is valid and presentable.

7. Show results of your in vitro quantifications please. The add-on activity of the T7 is interesting but your model does not really hold in cells (see below). It also does not agree with ref 37. Why is that? Please discuss the published results in comparison with yours.

8. The biosensor results (page 9) suggest that T678 and S686 mutants prevent inhibition (block glycosylation) in the HEK293 cells. You need to show such activity (glycosylation of the spike by the native HEK293 enzymes). That is a critical piece of information.

9. Information on the endogenous expression of the GalNAcTs is missing from your paper. Provide the quantities and activity. Do the HEK cell lysates glycosylate your peptide probes in vitro?

10. Is the furin-resistant mutant probe glycosylated (Figure 2)? Can you isolate that peptide from the cells and determine the glycosylation status? Please complete the GalNAcT KO and add compensatory KI with inducible constructs.

11. Why is overexpression of T3 and T7 needed for results in Figure 2c? Why not KO of the endogenous enzymes? Overexpression may lead to non-specific effects mediated by things other than the glycosylation of your probe.

12. “ …double mutation of these two potential glycosylation sites seemed to cause a slight increase in S2 production (Fig. 2d and Supplementary Fig. 5)”. Please show numbers instead of adjectives and discuss significance of your results. I find the presentation of bands insufficient - throughout the paper. The bands can go to the supplement and graphs or tabulated numbers into the paper.

13. “Because GalNAc-T7 displayed promiscuity towards the peptide substrate in vitro, we speculated that GalNAc-T7 may have other modification sites on the full length spike protein”. Please show that. The S689 activity is minor and only shown on an artificial substrate in vitro. You did not show that it happens on the full-length protein. I find impact of T7 on other proteins a more likely explanation. In addition, I do not understand what “promiscuous activity” means, please explain it or use clear terminology.

14. Why do you quantify the spike and its cleaved products in cell lysates? It is a secreted protein that should be measured in the media. The intracellular proteins may represent immature forms or degradation. The isolated viral particles should be compared to the secreted spike.

15. S Figure 5: KO of GalNT7 increases cleavage of the WT spike from 20 to 40%. Why are the mutants further cleaved to about 55% in the T7 KO cells? If the T7 makes the difference, there cannot be an additional impact of the mutants in the T7 KO cells. That suggests other activity of the T7.

16. S Figure 5: How can the cleavage of the T687/S686 spike differ between the WT and T7 KO cells when glycosylation of the responsible sites is eliminated?

17. “KI of GalNAc-T7 partially protected the spike protein from being processed, double KI of GALNT3 and GALNT7 almost completely blocked the production of S2 fragment from the overexpressed spike protein”. Why don’t you provide the numbers in the text? Your graphs do not show what you say – there is a marginal difference of maybe 25 vs 17 % for T7 vs dKI in SF8.

18. The syncytium results seem interesting but how was the quantification done? How many fields of view and cells were analyzed and how representative is the picture presented? Where is the image processing and statistical evaluation of the data described?

19. Why don’t you do the entire study on the Vero E6 cells as opposed to the HEK293 which is not really relevant?

20. Figure 3 compares cell pellets to the exported viral particles. Why cell pellets? Shouldn’t we look at secreted spike proteins? There is another layer of complexity related to what the cells secrete.

21. Figure 2f shows protection from the syncytium formation by T3/T7 double KI. What happens with T3 single KI or T7 single KI? Is the KI protective with spike mutants (T678 and/or S686)? It should not work when the target is missing.

22. “Interestingly, the double mutant did not produce more luminescence increment than S686A or T678A mutant, which is in line with the dependence of glycosylation at S686 on prior glycosylation at T678.” How do you reconcile the need for activity of both enzymes in the reporter assay (Figure 2b) with the result in Figure 2d where both mutations are required to observe an effect in the WT cells?

23. Why do you show GAPDH in Figure 2d or 2e? The ratio of intensities is important. Did you normalize by GAPDH? As I said before, please show the graphs and numbers instead of the bands. T-test is not adequate (page 41 legend).

24. What exactly do you quantify in Figure 3? Please describe it fully in the methods. There are double bands on top (FL-S) and in the cleaved product, sometimes. Are they all included in the analysis?

25. Why is the full-length protein in the VLP pellets in all the (WT and mutant) constructs with/out transfection of T3/T7 (Figure 3)? The glycosylation does not seem to make much difference. Provide tables of numbers and evaluate statistical differences please.

26. You suggest that cleaved spike binds to the extracellular domain of the M protein. Please provide direct evidence for their interaction (e.g. mutual co-IP experiments, SPR measurements, dependence of the binding on the EE).

27. Your assumption is that O-glycosylation is a major regulatory event of the S-M interaction but the evidence is mostly indirect. The KO and KI experiments represent extremes and can lead to undetermined effects (eg T7 can modify a number of other proteins, irrespective of the spike). Please document the degree of O-glycosylation of the spike and show that the genomic manipulations actually alter it, in line with the cleavage and interaction phenotypes.

Reviewer #2 (Remarks to the Author):

In this manuscript, the authors have investigated the roles of GalNAc-T3 and T7 glycosylation that supposedly inhibits furin processing of the S-protein and suppresses formation of virus like particles (VLPs), enabling a glycosylation-based protection by the host cells against the SARS-CoV-2. It is an interesting study however the manuscript is poorly developed and lacking clarity in many aspects which needs to be thoroughly addressed before considering for publication.

Introduction: It will be good to depict through a figure or text the furin cleavage sites/O-glycosylation sites on S-protein before delving into T678/R685 etc. The introduction is lacking citations – there are specific sentences with many references but there are several sentences that do not have any reference. Please check and modify. “As this manuscript is being prepared, an article by Benjamin Schumann” – please rephrase informal sentences like this in the manuscript. The aim part of the introduction needs to clearly mention what is unknown and what the study is aiming to do – the entire last paragraph explains the results straight away – were all variants of SARS-CoV-2 studied and why suddenly Omicron in human lung was addressed?

Method: Although there are several sub-headers of the different methods, it needs to be organized better. Cell culture/transfection/VLP production etc should be together and GalNac-studies should be clubbed together. LC-MS analysis should be a separate header. Why were different cell types used and were the cells grown in serum-free media at any point? How were the viral variants isolated from COVID19 patients and how were the variants confirmed? “Experiments related to authentic…” what kind of experiments? PCR amplification of spike DNA was of Wuhan? MS parameters: 150,000 seems unnecessary – any reason for that high resolution? It’s HCD type and the software is Proteome Discoverer. I am not sure if the Immunofluorescence microscopy and the results warrant for main text observations. Consider moving a few techniques to supplemental section (or partial descriptions) so readers can focus on the most important aspects of the study. Was the viral infection done by SARS-Cov-2 VLPs or isolated from COVID19 patients?

Results: The results/discussion section has a lot of haphazard information which is difficult to piece together. The sections need to be reorganized or at least the take home message should be

highlighted in some form – the manuscript might benefit from having a separate conclusion section that addresses the key points found in this study.

During GalNAcs in vitro testing, what observations were made for GalNAc-T7? The LC-MS method was EThCD I presume? What type of S-protein was used to test the furin inhibition by GalNAcs? HEK293-derived S-protein is not depiction of "biological" S-protein, was this hypothesis tested on isolated from patients? Again, for the mutation studies, was it the recombinant S-protein or isolated from patients? Interesting observation on overexpression of Galnac suppressing omicron – however, was this also seen in clinical samples that were used or only in the infected cell culture model?

Regarding the statement "O-glycosylation in spike protein are still obscure" – I disagree with this as there have been studies showing functional relevance of O-glycosylation – what is probably missing and addressed by this manuscript is its specific connection between GalNacT3/7 to furin inhibition.

Several typos in the manuscript – virion, theoretical etc. Please italicize N/O-glycosylation and m/z.

Reviewer #3 (Remarks to the Author):

Wang and colleagues investigated whether O-glycosylation near the furin cleavage site of the SARS-CoV-2 spike protein (S) impacts S protein cleavage, S protein-driven cell-cell fusion, S protein incorporation into particles and infection with authentic SARS-CoV-2. In brief, the authors demonstrate, using a peptide, that amino acid residues close to the cleavage site can be O-glycosylated and that GalNac-T3 and GalNac-T7 are responsible. Further, using surrogate systems for analysis of cleavage as well as directed S protein expression in cells, evidence is provided that mutation of certain O-glycosylation sites increases while overexpression of GalNac-T3/GalNac-T7 decreases cleavage and S protein-driven cell-cell fusion. In addition, overexpression of GalNac-T3/GalNac-T7 or mutation of an EE motive in the M protein are shown to decrease S protein incorporation into virus-like particles. Finally, the effect of overexpression of GalNac-T3/GalNac-T7 on S protein cleavage, S protein-driven cell-cell fusion and SARS-CoV-2 infection is shown to be modulated by naturally occurring mutations in the S protein. The concept proposed is of interest. However, it is insufficiently supported by data.

Major

It is important to demonstrate that O-glycosylation of the peptide studied here adequately mimics S protein O-glycosylation. Thus, the MS-based approach in figure 1 must be extended to WT and mutant S protein in infected cells.

Findings that suggest an impact of certain S protein residues on O-glycosylation, cleavage, and cell-cell fusion driven by overexpressed S protein must be confirmed with authentic SARS-CoV-2. This is essential, because expression levels and cellular trafficking of S protein in transfected and infected cells most likely differ. “Unfortunately, restricted by local law regulation, we cannot conduct reverse-engineering….” is not convincing.

The concept that S protein cleavage is important for virion incorporation is intriguing. However, it is well established that SARS-CoV-2 S protein quickly loses the furin cleavage site if cathepsin L is available for S protein activation in cell culture. This would not occur if loss of the furin cleavage site would result in poor particle incorporation of the S protein (unless particles are fully infectious despite incorporation of low amounts of S protein). Indeed, PMID: 33494095 demonstrated that loss of the furin cleavage site is fully compatible with efficient incorporation of spike into authentic SARS-CoV-2 particles.

It is proposed that an EE motive in the M protein interacts with the C-terminus of the S1 subunit. Please provide direct evidence for such an interaction. In the light of the published S protein structures – is this interaction physically possible given that the S1/S2 site might be significantly more membrane distal as compared to the EE motive?

Minor

The manuscript requires revision for grammar and style issues.

Reviewer: 1

The major concerns:

Query 1: Most of the studies were done in HEK293 cells, a marginally relevant model.

Response 1: We think this is a misunderstanding. The cell lines in this study are all commonly used cell lines for studying furin processing of Spike protein of SARS-CoV-2. As listed in references at the end of this rebuttal[1-7], different cell lines are used for different purposes. HEK293 is used to directly observe furin processing of overexpressed Spike because it does not express ACE2, which causes cell fusion upon Spike expression. Due to its high transfection efficiency, HEK293 is often the cell line of choice to assemble VLP and pseudovirus. Compared to HEK293, Vero-E6 has both ACE2 expression and relatively good transfection efficiency, thus it is a standard cell line to measure Spike-mediated syncytium formation. However, Vero-E6 is not a human lung cell line, therefore Calu-3 is often used for the final verification of authentic SARS-CoV-2. Our study used these different cell lines according to the textbook. No one directly does overexpression of Spike or assembly of VLP in Calu-3 because it has very poor transfection efficiency.

Query 2: A major shortcoming is the lack of any discussion of the endogenous expression of the GalNAcT enzymes. Which of the enzymes are expressed in the HEK or Vero cell lines and how does that affect the outcomes? The endogenous expression affects also many of the presented experimental results which needs to be clarified. For example, the biosensor experiments should be accompanied by KO of the T3 and T7 enzymes and subsequent compensation of the KO by inducible expression of the genes, as published in the original papers. The biosensor data (Figure 2b) or the spike cleavage data (Figure 2d and S Figure 5) show that the endogenous activity is significant.

Response 2: This is a good question regarding the endogenous expression of the GalNAc-T enzymes. However, there is a general assumption in studying O-glycosylation, which is that the occupancy of O-glycosylation is relatively low compared to N-glycosylation[8-19]. The usual way to do "addition" is to directly overexpress O-GalNAc transferases. In this way, we could observe the direct impact of increased T3 and T7 without worrying about endogenous enzymes. On the other hand, we chose to do "subtraction" by removing the glycosylation sites, which enabled functional studies of site-specific glycosylation. The reviewer's suggestion of KO of T3 and T7 and introducing back those enzymes is an alternative way to do "subtraction" and "addition" of glycosylations. But this comes with serious caveats: 1. KO of T3 and T7 will affect many other protein substrates in the cell than Spike; 2. KO of T3 and T7 will lead to compensational upregulation of other GalNAc-Ts in cells which may have non-specific activity toward the site being studied, thus complicating the results. Thus removing the glycosylation sites is the preferred way to do "subtraction".

To make our rationale visible to readers, we included the expression information for HEK293T, Vero-E6 and Calu-3 cells in the revised manuscript (Supplementary Fig. 5) and expanded the results sections regarding the endogenous activity of T3 and T7. Basically, all three cell lines we used in the study express T7 and the expression level for T3 is high in Calu-3, low in HEK293T, and very low in Vero-E6. Unfortunately, we cannot do reverse-engineering to mutate

the glycosylation sites in real viruses in our country, which is the only case we lack experiments with "subtraction" of T3 and T7 activity. We plan to carry out an authentic virus test in Calu-3 cells that lack of GalNAc-T7. We hope this incoming data will make our reviewer satisfied.

Query 3: The Western blot analyses are confusing. First, spike protein is secreted. Why do you analyze the spike proteins (full length and cleaved) in cell lysates? Second, the bands presented in the paper are not interpretable. Please show in the main text tables of data with clear statistical evaluation or the graphs from supplemental figures with full description of the analyses.
Response 3: First of all, there is a serious misunderstanding here. Spike protein is a single-pass type I membrane protein, not a secreted protein[20-22]. Therefore, we and the others [6, 23-29] analyzed Spike expression and cleavage in cell lysates.

Second, for a clearer presentation of our western blot results, the statistical analysis was in supplemental and now is moved to Figure 2. Results from biological replicates were included in Supplemental Fig. 7 and 9. We also added a detailed description of the analysis in the legend of Figure 2.

Query 4: The paper does not describe what statistical tests were used to compare the outcomes, besides t-test, which is inadequate. We want to know, for example, whether T3/T7 KI differs from T7 KI, in addition to whether it differs from the WT control. Please update the analyses and their descriptions; it is not clear what exactly was quantified (which bands, images) and how.
Response 4: We made changes to Figure 2 to include the statistical analysis for western blots and a detailed description of the analysis in the legend. Results from biological replicates were included in Supplemental Fig. 7 and 9. We used t-test for all the comparisons, which is standard for western blot analysis.

Query 5: The connection between O-glycosylation (T3/T7 model) and convertase cleavage of the spike is, overall, weak. Double mutation of the glycosylated amino acids changes cleavage of the spike from 20 to 30%; knock in of both T3 and T7 changes the cleavage from about 25% to 18%. At the same time, the baseline cleavage in WT cells changes from 20% to 30% without any apparent reason (SF5 and 8). The observed changes (due to KO or KI) are the maximal impacts expected and are rather small. A major impact of O-glycosylation in vivo needs therefore more evidence. Please discuss the quantitative aspects in full detail to support your model that connects convertase cleavage, viral assembly, and glycosylation. All the pieces likely happen, to some extent, but their interdependence and their impact on the disease outcomes may need additional evidence.
Response 5: First of all, we disagree that the connection between O-glycosylation and convertase cleavage is weak just because the baseline cleavage of Spike varies from batch to batch. The multibasic cleavage site of the Spike is evolved to be unstable. Therefore, the baseline cleavage of Spike cannot be directly compared between experiments carried out at different times or in different labs. For this reason, the baseline cleavage of the Spike can vary from 10% to 60% in literatures[6, 23-29].

Therefore, our data needs to be interpreted between the experimental groups carried out at the same time, where the conditions for cell harvest and protein extraction are strictly controlled between WT, T3 and/or T7 KI. In this way, the results are highly reproducible and we included

the replicate results in Supplementary Fig. 9 to support our claim.

On the other hand, we do agree that the impact of GalNAc-T3 and T7 on "overexpressed" Spike are rather small and cannot accurately reflect their effect on authentic viruses. That is why we tested the impact of GalNAc-T3 and T7 on the assembly of VLP as well as on the infection of authentic viruses. It is this discovery that distinguishes our work from previous studies that focus on "overexpressed" Spike.

Query 6: The proposed model of the M-S interaction needs further validation by direct measurements of the binding.

Response 6: This is a very good suggestion. After receiving the reviewer's comments, we performed co-IP experiment with M protein and full-length Spike to show the direct binding between these two proteins. The result below clearly demonstrated that M protein specifically interacted with S protein. In addition, the interaction was more prominent with cleaved S, in line with our proposed model of cleavage-dependent M-S interaction model. We are on the way to do more thorough studies with different M and S mutations and will include those data in the revised manuscript.

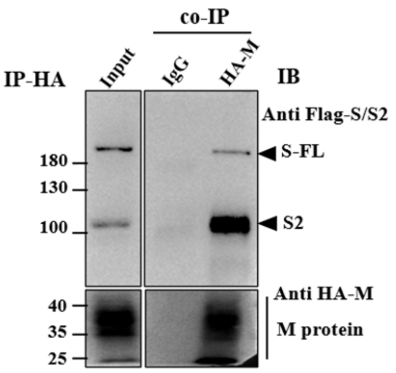

Figure 1. Co-IP experiment between M protein and full-length Spike protein. HEK293T cells were transfected with plasmids expressing full-length Spike protein (Flag-tagged) and M protein (HA-tagged). Cells were collected 48 hours post transfection and immunoprecipitation was performed from cell lysate using anti-HA antibody. Immunoblotting using anti-Flag antibody revealed co-precipitation of Spike protein and more cleaved Spike than full-length Spike was pulled down with M protein. Arrows indicate Spike proteins. Rabbit IgG served as a negative control.

Specific suggestions:

Query 1: Abstract line 2: what is "increased infectivity"? It is essential for infectivity or it increases infectivity.

Response 1: The sentence is changed to "plays an important role in viral infection" for clarity.

Query 2: Page 5 describes reports on O-glycosylation of the spike (ref22-33). The paper will be

stronger if you include a table (perhaps in the supplement) which describes clearly what sites were described as O-glycosylated and which of the reports do you find conclusive; the references are of mixed quality. Do you find the analyses in HEK293 cells sufficient? Do the studies agree in their conclusions? Does the evidence conclusively document O-glycosylation under relevant in vivo conditions? Is the extent of the glycosylation suggestive of a major regulatory impact? This deserves a better discussion.

Response 2: In previous reports, a number of *O*-glycosylation sites were identified from recombinant spike proteins and authentic viruses. T676 and T678 near the multibasic furin site were identified in several reports, while S686 was identified by Dr. Cummings, R. D [11]. We also performed glycol-profiling on recombinant Spike from engineered HEK293F (simple cell) and identified *O*-glycosylation on T678, S686 and S689 near the furin site. We didn't include our result in the original manuscript because we didn't want to present it as a profiling report and distract the audience. However, we did realize that the addition of our data actually made our manuscript stronger. S686, being right next to the arginine where furin cleaves, whose glycosylation and roles in viral regulation were demonstrated in our manuscript, was identified by both Prof Cummings and our study, making our story more compelling. In addition, we identified a novel glycosite, S689, which we also observed in *in vitro* glycosylation reaction by GalNAc-T7. Its glycosylation may well explain the difference we observed in the processing of Spike mutants in WT and T7-KO cells (see our response 22 to reviewer 1). So in the revised manuscript, we added a table that describes the O-glycosites reported in the literature (Supplementary Table 1) and identified in our study (Supplementary Fig. 1).

Query 3: The occupancy of the O-glycosites is particularly puzzling; a major inhibitory effect on convertase processing does not seem plausible based on a modification observed on a small percentage of the spike molecules. Please discuss and provide more evidence (on page 5 and throughout the text).

Response 3: It is a good question. In the literature, the occupancy of the identified O-glycosites on Spike is indeed low in general[8-19]. However, it should be noted that those observations were made on either the "overexpressed" extracellular domain of Spike protein or the "overexpressed" RBD domain, which cannot accurately reflect the occupancy on authentic viruses. The limited amount of Spike protein from authentic viruses makes reliable analysis of occupancy challenging. Because the virions of coronaviruses are assembled in the ER-Golgi intermediate compartment (ERGIC), the Spike protein accumulated in ERGIC can very much have a higher occupancy of the O-glycosites than on the secreted Spike fragments. That is exactly why our major discovery was that the O-glycosylation affects the assembly of VLP and authentic virus, which is a yes-or-no phenotype and much more significant than the inhibitory effect on "overexpressed" Spike proteins.

Finally, even for those low occupancy O-glycosites on "overexpressed" Spike proteins, two high-impact publications have already proved that they could modulate the proteolysis of SARS-CoV-2 Spike protein[24, 30]. So we think the inhibitory effect of O-glycosites on furin is plausible, just not so big on "overexpressed" proteins.

Query 4: Page 6 cites references describing that sialylation of the O-glycans is relevant. Your study did not look at the structures of the O-glycans at all. You simply report O-glycan yes/no

using methods which can hardly quantify (MALDI or ETD MS). The type of glycans and the extent of glycosylation (occupancy) in the spike protein remain undefined which limits the value of the study. This needs to be at least discussed extensively as a limitation of the study.
Response 4: O-glycan carrying proteins produced in HEK293T mainly contain ST or STn type O-glycan[31, 32], which means sialylation of the O-glycans is not the rate-limiting step. So there is an assumption in our study that O-glycans are mostly sialylated. We included a discussion session regarding the role of sialylation in O-glycan-mediated regulation of Spike processing by the convertase.

Query 5: Page 7 line 3: which harbors …
Response 5: We made changes in the introduction of the revised manuscript and deleted this sentence.

Query 6: Page 7 last line: "we found that GalNAc-T3 demonstrated the most substantial activity". What does that mean? Please define the numbers and show how much more active the T3 enzyme is than others. Is your readout quantitative? Are the enzymes compared under relevant conditions? Perhaps each of them requires a different set of conditions for optimal activity. Please discuss and document that your analysis is valid and presentable.
Response 6: We apologize for not using precise numbers. However, as we described in the methods section, all *in vitro* enzymatic reactions were carried out under the standard condition [33-36]. We calculated the conversion rate for T3 and T1 during *in vitro* reactions, which is 100% and 40.9% respectively, and added the numbers to the text accordingly.

Query 7: Show results of your in vitro quantifications please. The addon activity of the T7 is interesting but your model does not really hold in cells (see below). It also does not agree with ref 37. Why is that? Please discuss the published results in comparison with yours.
Response 7: Again, we apologize for not using precise quantification numbers and we calculated conversion rate of *in vitro* reactions and modified the text accordingly. However, we checked ref. 37 (Nat Med 26, 450-452 (2020)) and could not find any description of T7 in it. It is a Correspondence published right after the outbreak of Covid-19, in which the author envisioned potential O-glycosylation near furin site could in theory affect viral activation. We cited this reference as a rationale to look for essential glycosyltransferases in making the modifications.

In our analysis, the activity of T7 plays a major role in the glycosylation-mediated inhibition of furin processing as shown from Spike cleavage in overexpressed protein and VLPs. Our result does differ significantly from previous findings, which suggests the activity of T1 inhibits the furin processing of "overexpressed" Spike *in vitro*[24]. We could speculate two reasons for the difference. First, as the reviewer pointed out earlier, endogenous T7 could facilitate T1 in suppressing furin processing of Spike in the previous study because our results show they work in tandem. Secondly, as we emphasized above, we are not trying to argue which GalNAc-T can suppress the cleavage of "overexpressed" Spike more because overexpressed Spike cannot accurately model authentic viruses. One of our major novelties is the discovery of the impact of glycosyltranferases on the assembly of VLP, which mimics the encountering of GalNAc-T with virion in ERGIC. In this regard, overexpressing GalNAc-T1 has no effect on the assembly of VLP in our analysis. Per the reviewers' quest, we added a more detailed discussion on this in our main

text.

Query 8: The biosensor results (page 9) suggest that T678 and S686 mutants prevent inhibition (block glycosylation) in the HEK293 cells. You need to show such activity (glycosylation of the spike by the native HEK293 enzymes). That is a critical piece of information.
Response 8: As we already responded above to Query 2 of "Specific questions", those sites were identified as occupied O-glycosylation sites in literatures, which used Spike proteins overexpressed in HEK293 cells. In the revised manuscript, we also included our O-glycosites data on Spike produced in engineered HEK293F (Supplementary Fig.1), which confirms those sites are indeed modified. We also added a table describing O-glycosites reported in literature and found in our study (Supplementary Table 1).

Query 9: Information on the endogenous expression of the GalNAcTs is missing from your paper. Provide the quantities and activity. Do the HEK cell lysates glycosylate your peptide probes in vitro?
Response 9: This is the same as the Query 2 of "Major concerns". We already responded to it by providing expression profiles of GalNAc-Ts for HEK293T, Vero-E6 and Calu-3 cells in the revised manuscript (Supplementary Fig. 5). However, it is not feasible to perform the suggested in vitro glycosylation experiment with cell lysates, because the furin-site containing peptide is going to be digested by proteases in cell lysates before it meets the glycosyltransferases. As a matter of fact, due to the unstable nature of the furin-site of Spike, no one has ever tried a similar experiment in literature.

Query 10: Is the furin-resistant mutant probe glycosylated (Figure 2)? Can you isolate that peptide from the cells and determine the glycosylation status? Please complete the GalNAcT KO and add compensatory KI with inducible constructs.
Response 10: First of all, the furin-site containing biosensor probes were designed based on the sequence of the Spike protein and our synthetic peptide substrates, both of which have evidence for occupied glycosylation sites. We can try to purify the biosensors in the presence of T3 and T7 and show those sites can also be glycosylated similar to Spike and synthetic peptides. However, we do not see the purpose of the inducible expression of GalNAc-T, which, to our best knowledge, is used to avoid the toxicity of overexpressing oncogene and has only been applied to glycosyltransferases when the degree of glycosylation needs to be controlled [37, 38].

Query 11: Why is overexpression of T3 and T7 needed for results in Figure 2c? Why not KO of the endogenous enzymes? Overexpression may lead to non-specific effects mediated by things other than the glycosylation of your probe.
Response 11: Again, this is the same as Query 2 of "Major concerns". As we already explained above, "the occupancy of O-glycosylation is usually low in comparison to N-glycosylation". This is why we were able to observe the direct impact of overexpressed O-GalNAc transferases without too much worrying about endogenous enzymes. Overexpression may lead to non-specific effects, but that is the only way to increase the glycosylation level in cells. By the same token, KO of endogenous enzymes will also lead to "non-specific effects" stated by the reviewer. That is why we choose to mutate the glycosylation sites, which is more specific way to remove glycosylation

on the biosensors. Nevertheless, if the reviewer insists, we can perform the biosensor assay on T7 KO cell line and compare the results with that from the glycosites-mutated biosensors.

Query 12. " …double mutation of these two potential glycosylation sites seemed to cause a slight increase in S2 production (Fig. 2d and Supplementary Fig. 5)". Please show numbers instead of adjectives and discuss the significance of your results. I find the presentation of bands insufficient - throughout the paper. The bands can go to the supplement and graphs or tabulated numbers into the paper.
Response 12: We do have quantitative results from western blot analysis in the supplemental figures, and per the reviewer's quest we moved them to the main figures in the revised manuscript (Fig.2).

Query 13: "Because GalNAc-T7 displayed promiscuity towards the peptide substrate in vitro, we speculated that GalNAc-T7 may have other modification sites on the full length spike protein". Please show that. The S689 activity is minor and only shown on an artificial substrate in vitro. You did not show that it happens on the full-length protein. I find impact of T7 on other proteins a more likely explanation. In addition, I do not understand what "promiscuous activity" means, please explain it or use clear terminology.
Response 13: In addition to *in vitro* activity with the peptide, we did observe S689 glycosylation in purified full-length Spike and we've included the data in Supplementary Fig.1. We also added further discussion in the main text.

Query 14: Why do you quantify the spike and its cleaved products in cell lysates? It is a secreted protein that should be measured in the media. The intracellular proteins may represent immature forms or degradation. The isolated viral particles should be compared to the secreted spike.
Response 14: This is a repeat of Query 3 of "Major concerns". Please see the detailed responses above. Again, this reviewer seems to misunderstand that the Spike protein is a secreted protein. As a matter of fact, Spike protein of SARS-CoV-2 (and other enveloped viruses) is a single pass membrane protein, which stays on host cell membrane. Only when Spike protein is packed into virus particles (viral budding), it will be found in media.

Query 15: S Figure 5: KO of GalNT7 increases cleavage of the WT spike from 20 to 40%. Why are the mutants further cleaved to about 55% in the T7 KO cells? If the T7 makes the difference, there cannot be an additional impact of the mutants in the T7 KO cells. That suggests other activity of the T7.
Response 15: We explained our reasoning around this point in Discussion. This actually exemplify the downside of using KO of GalNAc-T to decrease the glycosylation. Because there are 20 members in GalNAc-T family, we speculate that KO of T7 will likely cause compensatory upregulation of other (less specific) GalNAc-Ts. That is why removing glycosites can further increase the Spike processing. As we stated above in the Query 2 of "Major concerns", mutating glycosites is a cleaner way than KO of glycosyltransferases. This is an inherent methodology limitation in studying O-Glycobiology.

Query 16: S Figure 5: How can the cleavage of the T687/S686 spike differ between the WT and

T7 KO cells when glycosylation of the responsible sites is eliminated?
Response 16: The same question as above.

Query 17: "KI of GalNAc-T7 partially protected the spike protein from being processed, double KI of GALNT3 and GALNT7 almost completely blocked the production of S2 fragment from the overexpressed spike protein". Why don't you provide the numbers in the text? Your graphs do not show what you say –there is a marginal difference of maybe 25 vs 17 % for T7 vs dKI in SF8.
Response 17: Again, we do have quantitative results from western blot analysis in the supplemental figures, and per the reviewer's quest we moved them to main figures in the revised manuscript. Secondly, we respectfully disagree that a statistically significant change from 27% to 17% is a "marginal difference".

Query 18: The syncytium results seem interesting but how was the quantification done? How many fields of view and cells were analyzed and how representative is the picture presented? Where is the image processing and statistical evaluation of the data described?
Response 18: We randomly chose between 9-12 syncytia for analysis and the quantitation was shown as the number of nuclei per syncytium. The pictures shown are representative views of each condition. We included the image processing and statistical evaluation in the legend for Figure 2.

Query 19: Why don't you do the entire study on the Vero E6 cells as opposed to the HEK293 which is not really relevant?
Response 19: Please see the response to Query 1 of the "Main concerns" for a more detailed explanation. The reviewer needs to understand that different cell lines have different characteristics to meet the different purposes of experiments. Vero E6 is frequently used for entry analysis of pseudovirus and syncytium analysis due to the expression of ACE2, but for the same reason it cannot be used to study the processing of overexpressed Spike, neither the production of VLP. In addition, although Vero E6 is frequently used to "produce" authentic virus, it is a kidney cell line from African green monkey and cannot be used to model biological effects on human lung cells.

Query 20: Figure 3 compares cell pellets to the exported viral particles. Why cell pellets? Shouldn't we look at secreted spike proteins? There is another layer of complexity related to what the cells secrete.
Response 20: Again, this is a repeat of Query 3 of "Major concerns", as well as Query 14 of "Specific questions". The reviewer needs to understand that Spike protein is a membrane protein, not a secreted protein.

Query 21: Figure 2f shows protection from the syncytium formation by T3/T7 double KI. What happens with T3 single KI or T7 single KI? Is the KI protective with spike mutants (T678 and/or S686)? It should not work when the target is missing.
Response 21: We added the data to Fig. 2 in the revised manuscript.

Query 22: "Interestingly, the double mutant did not produce more luminescence increment than

S686A or T678A mutant, which is in line with the dependence of glycosylation at S686 on prior glycosylation at T678." How do you reconcile the need for activity of both enzymes in the reporter assay (Figure 2b) with the result in Figure 2d where both mutations are required to observe an effect in the WT cells?

Response 22: The reporter is a simplified system where other glycosylation sites in Spike are not considered. However, with full-length Spike, other glycosylation sites in the protein may also provide some protection against cleavage, making the effect of a single mutation less obvious. In the T7-KO background, the effect becomes more significant. We speculate that the additional glycosylation on S689 we observed in the T7 reaction might be partially responsible for it.

Query 23: Why do you show GAPDH in Figure 2d or 2e? The ratio of intensities is important. Did you normalize by GAPDH? As I said before, please show the graphs and numbers instead of the bands. T-test is not adequate (page 41 legend).

Response 23: We use GAPDH as the loading control for western blot analysis.

Query 24: What exactly do you quantify in Figure 3? Please describe it fully in the methods. There are double bands on top (FL-S) and in the cleaved product, sometimes. Are they all included in the analysis?

Response 24: The different bands on top are Spike monomer and oligomers. The cleaved product is S2 and S2'. Comparisons were made between cleaved S (S2 and S2') and FL-S (monomer and oligomers). We added details for the analysis in the legend for Figure 2.

Query 25: Why is the full-length protein in the VLP pellets in all the (WT and mutant) constructs with/out transfection of T3/T7 (Figure 3)? The glycosylation does not seem to make much difference. Provide tables of numbers and evaluate statistical differences please.

Response 25: We and others[1, 2, 39 40]do observe full-length Spike in the VLP pellets. We have to say that glycosylation affects VLP assembly as we show but it is not the only regulatory mechanism and the question is outside the scope of this study. That is why in the main text we keep using the wording such as: affects, facilitates,…etc. Basically, we cannot look at Biology in such a mechanic way.

Query 26: You suggest that the cleaved spike binds to the extracellular domain of the M protein. Please provide direct evidence for their interaction (e.g. mutual co-IP experiments, SPR measurements, dependence of the binding on the EE).

Response 26: This is a repeat of Query 6 of "Major concerns". Please see our response there for our coIP results.

Query 27: Your assumption is that O-glycosylation is a major regulatory event of the S-M interaction but the evidence is mostly indirect. The KO and KI experiments represent extremes and can lead to undetermined effects (eg T7 can modify a number of other proteins, irrespective of the spike). Please document the degree of O-glycosylation of the spike and show that the genomic manipulations actually alter it, in line with the cleavage and interaction phenotypes.

Response 27: Please see the response to Specific Suggestions Query 8.

Reviewer: 2

Query 1: It will be good to depict through a figure or text the furin cleavage sites/O-glycosylation sites on S-protein before delving into T678/R685 etc.
Response 1: It's a good suggestion and we have moved such a figure from supplemental to Figure 1a.

Query 2: The introduction is lacking citations – there are specific sentences with many references but there are several sentences that do not have any reference.
Response 2: We made corrections to the citations so that they appear where mentioned.

Query 3: Please check and modify. "As this manuscript is being prepared, an article by Benjamin Schumann" – please rephrase informal sentences like this in the manuscript.
Response 3: We changed the sentence to: "A recent study using a chemical probe strategy demonstrated that furin processing is affected by sialyation of the extended O-glycan at T678.

Query 4: The aim part of the introduction needs to clearly mention what is unknown and what the study is aiming to do – the entire last paragraph explains the results straight away – were all variants of SARS CoV-2 studied and why suddenly Omicron in human lung was addressed?
Response 4: We have rewritten the aim part of the introduction.

Query 5: Although there are several sub-headers of the different methods, it needs to be organized better. Cell culture/ transfection/VLP production etc should be together and GalNac-studies should be clubbed together. LC-MS analysis should be a separate header.
Response 5: We have added LC-MS analysis as a separate header in methods.

Query 6: Why were different cell types used and were the cells grown in serum-free media at any point?
Response 6: Please see response to Query 1 from Reviewer 1. In the original manuscript, all the cells were grown in the presence of serum, however, we added glyco-analysis on Spike purified from FreeStyle™ 293-F, which was grown in serum-free media. This information is included in the method part of the revised manuscript.

Query 7: How were the viral variants isolated from COVID19 patients and how were the variants confirmed? "Experiments related to authentic…" what kind of experiments? PCR amplification of spike DNA was of Wuhan?
Response 7: In our manuscript, the SARS-CoV-2 variants, including WT, alpha (B.1.1.7), and Omicron (BA.1) were isolated from COVID-19 patients. All viruses were purified by plaque assay and confirmed by next-generation sequencing. We have modified the Method section in the revised manuscript.
Query 8: MS parameters: 150,000 seems unnecessary – any reason for that high resolution? It's HCD type and the software is Proteome Discoverer.
Response 8: We apologize for the typo. It should be 50,000 rather than 150,000. We've corrected

the typo. Since we added the MS analysis result of Spike in the main text, we made corresponding changes in the Method section.

Query 9: I am not sure if the Immunofluorescence microscopy and the results warrant for main text observations.
Response 9: We are not sure what the reviewer's concern is. But the IF images in Figure 2F were representative views from each condition. For quantitative analysis, we randomly chose between 9-12 syncytia for analysis and the quantitation was shown as the number of nuclei per syncytium. The picture showed a representative view. We included image processing and statistical evaluation in the figure legend.

Query 10: Consider moving a few techniques to supplemental section (or partial descriptions) so readers can focus on the most important aspects of the study.
Response 10: We have carefully gone through the entire manuscript to make the key point clearer and the logic flows better.

Query 11: Was the viral infection done by SARS-Cov-2 VLPs or isolated from COVID19 patients?
Response 11: Viral infections were performed with authentic viruses isolated from COVID19 patients.

Query 12: The results/discussion section has a lot of haphazard information which is difficult to piece together.
Response 12: We have carefully gone through the entire manuscript to make the key point clearer and the logic flows better.

Query 13: The sections need to be reorganized or at least the take home message should be highlighted in some form – the manuscript might benefit from having a separate conclusion section that addresses the key points found in this study.
Response 13: It's a good suggestion. We have added a separate conclusion section that addresses the key points found in our study

Query 14: During GalNAcs in vitro testing, what observations were made for GalNAc-T7?
Response 14: T7 only glycosylated the peptide with prior glycosylation at S678.

Query 15: The LC-MS method was EThCD I presume?
Response 15: It is ETciD. We added a separate subleader for LC-MS in the methods.

Query 16: What type of S-protein was used to test the furin inhibition by GalNAcs? HEK293-derived S-protein is not depiction of "biological" S-protein, was this hypothesis tested on isolated from patients? Again, for the mutation studies, was it the recombinant S-protein or isolated from patients?
Response 16: The S protein, both native and mutants, is recombinant and the encoding DNA sequence is derived from Wuhan-1 SARS-CoV-2.

Query 17: Interesting observation on overexpression of Galnac suppressing omicron – however, was this also seen in clinical samples that were used or only in the infected cell culture model?
Response 17: The study is carried out in Calu-3, which is a lung cell model. The clinical study is beyond the scope of this manuscript.

Query 18: Regarding the statement "O-glycosylation in spike protein are still obscure" – I disagree with this as there have been studies showing functional relevance of O-glycosylation – what is probably missing and addressed by this manuscript is its specific connection between GalNacT3/7 to furin inhibition.
Response 18: To make our point clearer, we changed the sentence to "the relevance of Spike O-glycosylation in viral infection is still not clear". Previous studies have revealed sites of O-glycosylation in Spike and its potential regulation in furin processing, however, we for the first time showed the connection between O-glycosylation and viral infection.

Query 19: Several typos in the manuscript – virion, theoretical etc.
Response 19: We've corrected the typos in the revised manuscript.

Query 20: Please italicize N/O-glycosylation and m/z.
Response 20: We've made corresponding corrections in the revised manuscript.

Reviewer: 3

The major comments:

Query 1: It is important to demonstrate that O-glycosylation of the peptide studied here adequately mimics S protein O-glycosylation. Thus, the MS-based approach in figure 1 must be extended to WT and mutant S protein in infected cells.
Response 1: We have added glyco-analysis data on S protein purified from HEK293, which confirmed our in vitro studies with the peptide. However, it is technically challenging to isolate S protein from authentic viruses to do O-glycosylation analysis. We will carry out this analysis on Spike from VLP to further address this concern.

Query 2: Findings that suggest an impact of certain S protein residues on O-glycosylation, cleavage, and cell-cell fusion driven by overexpressed S protein must be confirmed with authentic SARS-CoV-2. This is essential, because expression levels and cellular trafficking of S protein in transfected and infected cells most likely differ. "Unfortunately, restricted by local law regulation, we cannot conduct reverse-engineering…." is not convincing.
Response 2: The most convincing way to confirm the impact of site-specific O-glycosylation on viral infection is to do reverse engineering to mutate the O-glycosylation site in authentic virus, however, this kind of experiment is not prohibited by law in China. Therefore, we have demonstrated this connection in VLP with glycosite mutations, which maximally mimics cellular

trafficking of S protein during viral infection without disobeying local law. In addition, We plan to carry out an authentic virus test in Calu-3 cells that lack of GalNAc-T7. We hope this incoming data will make our reviewer satisfied.

Query 3: The concept that S protein cleavage is important for virion incorporation is intriguing. However, it is well established that SARS-CoV-2 S protein quickly loses the furin cleavage site if cathepsin L is available for S protein activation in cell culture. This would not occur if loss of the furin cleavage site would result in poor particle incorporation of the S protein (unless particles are fully infectious despite incorporation of low amounts of S protein). Indeed, PMID: 33494095 demonstrated that loss of the furin cleavage site is fully compatible with efficient incorporation of spike into authentic SARS-CoV-2 particles.

Response 3: It is a very good point. As a matter of fact, PMID: 33494095 demonstrated that loss of furin cleavage site attenuates SARS-CoV-2 pathogenesis as the ΔPRRA mutant had ~10-fold reductions in viral titer and less spike incorporation in Calu-3 (see below). This is very much consistent with our results showing VLP assembly is dependent on furin cleavage and GalNAc-Ts provide protection against viral infection. However, the authors of this paper also stated: "the results are complicated by augmented replication and fitness in Vero E6 cells driven by the absence of TMPRSS2 expression". Our explanation for this discrepancy is that furin cleavage is one of the many pathways that eventually lead to Spike activation and it may play more critical roles in certain cells and less in others. So our work and PMID: 33494095 proved the regulatory function of furin and glycosylation in human lung cell Calu-3. However, it requires more work to understand the different pathways that could lead to Spike activation in various cell types.

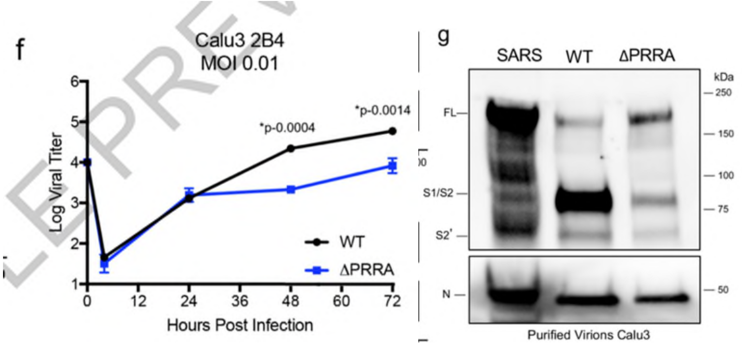

Query 4: It is proposed that an EE motive in the M protein interacts with the C-terminus of the S1 subunit. Please provide direct evidence for such an interaction. In the light of the published S protein structures – is this interaction physically possible given that the S1/S2 site might be significantly more membrane distal as compared to the EE motive?

Response 4: This is a similar concern as reviewer 1 pointed out. I believe our IP result as shown in our response to Query 6 of reviewer 1 can partially address this concern. Structurally, it is difficult to predict how the interaction exactly happens because Spike undergoes substantial conformational changes upon furin cleavage and even the prefusion isoform adopts various conformations. Nevertheless, we plan to further validate the interaction between S and M by co-IP using different mutants to address this concern.

The minor comments:

Query1: The manuscript requires revision for grammar and style issues.
Response 1: Thank you. We have revised the whole manuscript carefully and tried to avoid any grammar or syntax errors. We believe that the language is now acceptable for the review process.

References:

1. Yurkovetskiy, L. et al. Structural and Functional Analysis of the D614G SARS-CoV-2 Spike Protein Variant. *Cell* **183**, 739-751 e738 (2020).
2. Syed, A.M. et al. Rapid assessment of SARS-CoV-2-evolved variants using virus-like particles. *Science* **374**, 1626-1632 (2021).
3. Hoffmann, M., Kleine-Weber, H. & Pohlmann, S. A Multibasic Cleavage Site in the Spike Protein of SARS-CoV-2 Is Essential for Infection of Human Lung Cells. *Mol Cell* **78**, 779-784 e775 (2020).
4. Yang, Q. et al. Inhibition of SARS-CoV-2 viral entry upon blocking N- and O-glycan elaboration. *Elife* **9** (2020).
5. Papa, G. et al. Furin cleavage of SARS-CoV-2 Spike promotes but is not essential for infection and cell-cell fusion. *PLoS Pathog* **17**, e1009246 (2021).
6. Xia, S. et al. The role of furin cleavage site in SARS-CoV-2 spike protein-mediated membrane fusion in the presence or absence of trypsin. *Signal Transduct Target Ther* **5**, 92 (2020).
7. Ou, X. et al. Characterization of spike glycoprotein of SARS-CoV-2 on virus entry and its immune cross-reactivity with SARS-CoV. *Nat Commun* **11**, 1620 (2020).
8. Shajahan, A., Supekar, N.T., Gleinich, A.S. & Azadi, P. Deducing the N- and O-glycosylation profile of the spike protein of novel coronavirus SARS-CoV-2. *Glycobiology* **30**, 981-988 (2020).
9. Shajahan, A., Pepi, L.E., Kumar, B., Murray, N.B. & Azadi, P. Site specific N- and O-glycosylation mapping of the spike proteins of SARS-CoV-2 variants of concern. *Sci Rep* **13**, 10053 (2023).
10. Sanda, M., Morrison, L. & Goldman, R. N- and O-Glycosylation of the SARS-CoV-2 Spike Protein. *Anal Chem* **93**, 2003-2009 (2021).
11. Gao, C. et al. SARS-CoV-2 Spike Protein Interacts with Multiple Innate Immune Receptors. *bioRxiv* (2020).
12. Zhao, P. et al. Virus-Receptor Interactions of Glycosylated SARS-CoV-2 Spike and Human ACE2 Receptor. *Cell Host Microbe* **28**, 586-601 e586 (2020).
13. Wang, D. et al. Comprehensive Analysis of the Glycan Complement of SARS-CoV-2 Spike Proteins Using Signature Ions-Triggered Electron-Transfer/Higher-Energy Collisional Dissociation (EThcD) Mass Spectrometry. *Anal Chem* **92**, 14730-14739 (2020).
14. Bagdonaite, I. et al. Site-Specific O-Glycosylation Analysis of SARS-CoV-2 Spike Protein Produced in Insect and Human Cells. *Viruses* **13** (2021).
15. Tian, Y., Parsons, L.M., Jankowska, E. & Cipollo, J.F. Site-Specific Glycosylation Patterns of the SARS-CoV-2 Spike Protein Derived From Recombinant Protein and Viral WA1 and

D614G Strains. *Front Chem* **9**, 767448 (2021).
16. Zhang, Y. et al. O-Glycosylation Landscapes of SARS-CoV-2 Spike Proteins. *Front Chem* **9**, 689521 (2021).
17. Tian, W. et al. O-glycosylation pattern of the SARS-CoV-2 spike protein reveals an "O-Follow-N" rule. *Cell Res* **31**, 1123-1125 (2021).
18. Brun, J. et al. Assessing Antigen Structural Integrity through Glycosylation Analysis of the SARS-CoV-2 Viral Spike. *ACS Cent Sci* **7**, 586-593 (2021).
19. Roberts, D.S. et al. Distinct core glycan and O-glycoform utilization of SARS-CoV-2 Omicron variant Spike protein RBD revealed by top-down mass spectrometry. *Chem Sci* **13**, 10944-10949 (2022).
20. Yao, H. et al. Molecular Architecture of the SARS-CoV-2 Virus. *Cell* **183**, 730-738 e713 (2020).
21. Cattin-Ortola, J. et al. Sequences in the cytoplasmic tail of SARS-CoV-2 Spike facilitate expression at the cell surface and syncytia formation. *Nat Commun* **12**, 5333 (2021).
22. Walls, A.C. et al. Structure, Function, and Antigenicity of the SARS-CoV-2 Spike Glycoprotein. *Cell* **181**, 281-292 e286 (2020).
23. Tanikawa, T. et al. Degradative Effect of Nattokinase on Spike Protein of SARS-CoV-2. *Molecules* **27** (2022).
24. Zhang, L. et al. Furin cleavage of the SARS-CoV-2 spike is modulated by O-glycosylation. *Proc Natl Acad Sci U S A* **118** (2021).
25. Lavie, M., Dubuisson, J. & Belouzard, S. SARS-CoV-2 Spike Furin Cleavage Site and S2' Basic Residues Modulate the Entry Process in a Host Cell-Dependent Manner. *J Virol* **96**, e0047422 (2022).
26. Saito, A. et al. Enhanced fusogenicity and pathogenicity of SARS-CoV-2 Delta P681R mutation. *Nature* **602**, 300-306 (2022).
27. Zhang, L. et al. SARS-CoV-2 spike-protein D614G mutation increases virion spike density and infectivity. *Nat Commun* **11**, 6013 (2020).
28. Bestle, D. et al. TMPRSS2 and furin are both essential for proteolytic activation of SARS-CoV-2 in human airway cells. *Life Sci Alliance* **3** (2020).
29. Hoffmann, M. et al. SARS-CoV-2 Cell Entry Depends on ACE2 and TMPRSS2 and Is Blocked by a Clinically Proven Protease Inhibitor. *Cell* **181**, 271-280 e278 (2020).
30. Gonzalez-Rodriguez, E. et al. O-Linked Sialoglycans Modulate the Proteolysis of SARS-CoV-2 Spike and Likely Contribute to the Mutational Trajectory in Variants of Concern. *ACS Central Science* (2023).
31. Kudelka, M.R. et al. Cellular O-Glycome Reporter/Amplification to explore O-glycans of living cells. *Nat Methods* **13**, 81-86 (2016).
32. Fujitani, N. et al. Total cellular glycomics allows characterizing cells and streamlining the discovery process for cellular biomarkers. *Proc Natl Acad Sci U S A* **110**, 2105-2110 (2013).
33. Ten Hagen, K.G. et al. Characterization of a UDP-GalNAc:polypeptide N-acetylgalactosaminyltransferase that displays glycopeptide N-acetylgalactosaminyltransferase activity. *J Biol Chem* **274**, 27867-27874 (1999).
34. Bennett, E.P. et al. Control of mucin-type O-glycosylation: a classification of the polypeptide GalNAc-transferase gene family. *Glycobiology* **22**, 736-756 (2012).

35. Kong, Y. et al. Probing polypeptide GalNAc-transferase isoform substrate specificities by in vitro analysis. *Glycobiology* **25**, 55-65 (2015).
36. de Las Rivas, M. et al. The interdomain flexible linker of the polypeptide GalNAc transferases dictates their long-range glycosylation preferences. *Nat Commun* **8**, 1959 (2017).
37. Chang, M.M. et al. Small-molecule control of antibody N-glycosylation in engineered mammalian cells. *Nat Chem Biol* **15**, 730-736 (2019).
38. Hintze, J. et al. Probing the contribution of individual polypeptide GalNAc-transferase isoforms to the O-glycoproteome by inducible expression in isogenic cell lines. *J Biol Chem* **293**, 19064-19077 (2018).
39. Sullivan, E. et al. SARS-CoV-2 Virus-like Particles Produced by a Single Recombinant Baculovirus Generate Anti-S Antibody and Protect against Variant Challenge. *Viruses* **14** (2022).
40. Xu, R., Shi, M., Li, J., Song, P. & Li, N. Construction of SARS-CoV-2 Virus-Like Particles by Mammalian Expression System. *Front Bioeng Biotechnol* **8**, 862 (2020).

Reviewer #1 (Remarks to the Author):

The authors answered my questions satisfactorily. I do not have any further comments.

Reviewer #2 (Remarks to the Author):

The authors have addressed my concerns appropriately.

Reviewer #3 (Remarks to the Author):

The authors have partially addressed the questions raised by this reviewer:

The demonstration of O-glycoslyation in the context of spike (and not only peptides) is important and increases the impact of the manuscript.

The absence of mutational analysis of potential sites for O-glycosylation in the context of virus is a significant weakness and must be clearly stated.

This reviewer disagrees with the argument of the authors that PMID: 33494095 supports their findings. PMID: 33494095 shows that the furin recognition sites needs to be intact for lung cell infection, most likely because it is needed for S protein activation. However the study also shows that cleavage is at least partially dispensable for particle incorporation of spike. Another milestone study in the field - PMID: 33907312 - reaches a similar conclusion: The furin recognition site is essential for robust lung cell infection but fully dispensable for particle incorporation of spike, see figure 1D, which demonstrates that uncleaved spike is incorporated into authentic SARS-CoV-2 with high efficiency - very much in contrast to the findings reported in the present study with a surrogate system. Statements like " However, it is why the contribution of furin processing to virion assembly remained undiscovered until now. One plausible explanation is that previous studies almost exclusively used pseudotyped virions to simulate the protease-dependent activation process of SARS-CoV-2." are thus incorrect and must be removed. In fact, the discrepancy between findings made with authentic virus (published work) and the surrogate system (present study) regarding the relevance of cleavage for particle incorporation of S protein should be discussed in detail.

The demonstration of M interactions with S is important and appreciated.

Reviewer #1 (Remarks to the Author):

The authors answered my questions satisfactorily. I do not have any further comments.

Reviewer #2 (Remarks to the Author):

The authors have addressed my concerns appropriately.

Reviewer #3 (Remarks to the Author):

The authors have partially addressed the questions raised by this reviewer:

The demonstration of O-glycosylation in the context of spike (and not only peptides) is important and increases the impact of the manuscript.

Response: The authors thank the reviewer for the previous suggestion.

The absence of mutational analysis of potential sites for O-glycosylation in the context of virus is a significant weakness and must be clearly stated.

Response: Thank you again for your suggestion. We agree that reverse-engineering SARS-CoV-2 with potential O-glycosites mutated and testing its susceptibility to GalNAc-T3 and T7 would significantly strengthen our conclusion. However, as previously mentioned, there are legal restrictions on conducting mutational studies with authentic SARS-CoV-2 in our country. Nevertheless, we concur that demonstrating the impact of O-glycosylation at specific sites in a pertinent system would enhance the manuscript's robustness. Hence, we assessed the effect of T678/S686 mutations on S protein incorporation into VLPs. As shown in the Figure below, S protein assembly into virions was greatly reduced by T3 and T7, however, the T678/S686 double mutant was not affected. This data strongly indicates the involvement of T678 and S686, most likely through O-glycosylation, in S protein cleavage and its assembly into virions. We have added this result to Figure 3c of the revised manuscript (line 283-288).

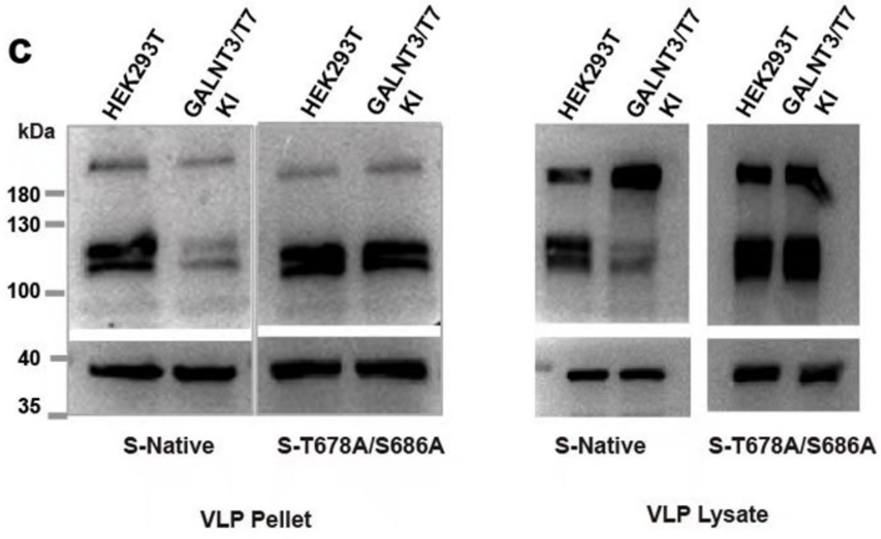

In addition, we amended the Conclusion and Discussion with the following sentences to clearly state the limitation of our study (line 534 to 538):

"Due to local legal regulations, we are unable to perform reverse engineering through point mutations on SARS-CoV-2 to study the impact of site-specific glycosylations. As a result, we could not rule out the possibility that the inhibition of authentic SARS-CoV-2 viruses by GalNAc-T3 and T7 is partly influenced by glycosylation sites other than those adjacent to furin processing sites."

This reviewer disagrees with the argument of the authors that PMID: 33494095 supports their findings. PMID: 33494095 shows that the furin recognition sites needs to be intact for lung cell infection, most likely because it is needed for S protein activation. However the study also shows that cleavage is at least partially dispensable for particle incorporation of spike. Another milestone study in the field - PMID: 33907312 - reaches a similar conclusion: The furin recognition site is essential for robust lung cell infection but fully dispensable for particle incorporation of spike, see figure 1D, which demonstrates that uncleaved spike is incorporated into authentic SARS-CoV-2 with high efficiency - very much in contrast to the findings reported in the present study with a surrogate system. Statements like " However, it is why the contribution of furin processing to virion assembly remained undiscovered until now. One plausible explanation is that previous studies almost exclusively used pseudotyped virions to simulate the protease-dependent activation process of SARS-CoV-2." are thus incorrect and must be removed. In fact, the discrepancy between findings made with authentic virus (published work) and the surrogate system (present study) regarding the relevance of cleavage for particle incorporation of S protein should be discussed in detail.

Response: The authors thank the reviewer for highlighting the issue concerning the necessity of furin processing for incorporating the S protein into authentic SARS-CoV-2. Indeed, both the literature (PMID: 33494095 and PMID: 33907312) show that removing furin-processing site only partially inhibits the particle incorporation of Spike in Calu-3 and does not impact the quantity of Spike incorporated in Vero cells. The disparity between our VLP result and these authentic viruses may stem from the ability of the authentic virus to replicate. In other words, live viruses might be able to compensate for the inefficient particle incorporation of uncleaved S protein by upregulating protein expression and other factors. The ability of live virus to cope with uncleaved S protein could also be host cell dependent as the incorporation rate of furin site-removed S protein is less efficient in Calu-3 than in Vero.

We acknowledge that VLP represents a simplified system, isolating viral assembly from other steps in the virus life cycle. Furthermore, we have observed a small amount of uncleaved Spike in VLP, along with the interaction of the M protein with uncleaved S protein (albeit to a lesser extent than processed S protein). Therefore, we are cautious about using terms like "necessary" or "required," and instead describe the outcome as "facilitating the incorporation of Spike into VLP."

Per the reviewer's suggestion, we have now deleted the incorrect statement mentioned (line 293-296) and have included a discussion section on the dependence of S protein assembly on furin activation in our VLP system and compared it with previously published results (line 499 to 507).

The demonstration of M interactions with S is important and appreciated.

Response: Thank you.

Reviewer #3 (Remarks to the Author):

The authors have adequately addressed my questions.
